# Equitable access to COVID-19 vaccines makes a life-saving difference to all countries

**DOI:** 10.1038/s41562-022-01289-8

**Published:** 2022-01-31

**Authors:** Yang Ye, Qingpeng Zhang, Xuan Wei, Zhidong Cao, Hsiang-Yu Yuan, Daniel Dajun Zeng

**Affiliations:** 1 School of Data Science, City University of Hong Kong, Hong Kong, China; 2 Antai College of Economics and Management, Shanghai Jiao Tong University, Shanghai, China; 3 The State Key Laboratory of Management and Control for Complex Systems, Institute of Automation, Chinese Academy of Sciences, Beijing, China; 4 School of Artificial Intelligence, University of Chinese Academy of Sciences, Beijing, China; 5 Department of Biomedical Sciences, Jockey Club College of Veterinary Medicine and Life Sciences, City University of Hong Kong, Hong Kong, China; 6 Centre for Applied One Health Research and Policy Advice, Jockey Club College of Veterinary Medicine and Life Sciences, City University of Hong Kong, Hong Kong, China

**Keywords:** Complex networks, Complex networks

## Abstract

Despite broad agreement on the negative consequences of vaccine inequity, the distribution of COVID-19 vaccines is imbalanced. Access to vaccines in high-income countries (HICs) is far greater than in low- and middle-income countries (LMICs). As a result, there continue to be high rates of COVID-19 infections and deaths in LMICs. In addition, recent mutant COVID-19 outbreaks may counteract advances in epidemic control and economic recovery in HICs. To explore the consequences of vaccine (in)equity in the face of evolving COVID-19 strains, we examine vaccine allocation strategies using a multistrain metapopulation model. Our results show that vaccine inequity provides only limited and short-term benefits to HICs. Sharper disparities in vaccine allocation between HICs and LMICs lead to earlier and larger outbreaks of new waves. Equitable vaccine allocation strategies, in contrast, substantially curb the spread of new strains. For HICs, making immediate and generous vaccine donations to LMICs is a practical pathway to protect everyone.

## Abstract (web summary)

Using data-driven mathematical modelling that combines viral evolution with epidemiological dynamics, Ye et al. show that COVID-19 vaccine inequity leads to the emergence of new variants and new waves of the pandemic, while equitable allocation of vaccine doses reduces case counts and fatalities in all countries.

## Main

Coronavirus disease 2019 (COVID-19) has had devastating impacts on economies, education, health care and social activities in an increasingly globalized world^1,2^. Non-pharmaceutical public health measures, such as border restrictions, social distancing and school closures, have been shown to be insufficient to fully contain the pandemic. Vaccines for COVID-19 are much needed to stop the spread and mutation of the disease and to reopen the world. Because of globalization, there will always be the risk of new outbreaks if vaccines are not widely available. As of 31 December 2021, 31 different vaccines had been approved by at least one country (https://covid19.trackvaccines.org/). More than 331 vaccine candidates are in development, of which 137 are in the clinical development phase^3^.

COVID-19 vaccination campaigns were underway worldwide in 2021. As of 31 December 2021, more than nine billion COVID-19 vaccination doses had been administered worldwide (approximately 116 doses per 100 people)^4^. However, country-level and regional vaccination rates are unbalanced. Over 70% of people in high-income countries (HICs) are fully vaccinated against COVID-19; in low-income countries, that number is 4%. Although no country has reported a fully vaccinated population, HICs seem to have access to enough vaccines to vaccinate their populations several times over, leaving many low- and middle-income countries (LMICs) struggling to obtain vaccine supplies to vaccinate their population even once (https://launchandscalefaster.org/COVID-19).

Many researchers and public health experts have warned of the negative consequences of global vaccine inequity^5–10^. Pandemics know no borders, and the public health and economic costs of inequitable vaccine allocation will be borne by all countries in the end. It has been shown in the context of influenza that cross-border vaccination subsidies could provide substantial indirect protection to countries donating vaccines^11,12^. Offering influenza vaccines to neighbouring countries can substantially reduce infections and deaths in both donating and receiving countries. Although many HICs have participated in the COVAX Facility (a joint fund backed by the World Health Organization to ensure equitable distribution of COVID-19 vaccines^13,14^), there remains an acute imbalance in the distribution of COVID-19 vaccines worldwide. Global vaccine inequity is twofold: first, due to the voluntary nature of COVAX, HICs continue to prioritize bilateral deals with vaccine suppliers, leaving scarce vaccine supplies for COVAX^15,16^; second, HICs seem to have underestimated the threat of new strains^17–21^ and thus are racing to vaccinate their entire populations and expand booster-shot programmes^22–25^ rather than donate vaccines to LMICs to suppress the emergence of new strains. The emergence of the Delta strain^26,27^ and the Omicron strain^21^ highlights the importance of (1) obtaining a better data-driven understanding of the role of global vaccine equity in preventing the emergence and spread of new strains and (2) identifying a practical pathway to global vaccine equity that reduces morbidity and mortality in both HICs and LMICs. The objective is to transform the global vaccine distribution from a “zero-sum game”^9^ to a “cooperative game”^28^. Such a game-theoretic approach has been applied to the control of other pathogens^29–34^. Numerical and analytical results based on hypothetical networks show that the optimal drug/vaccine coordination can reduce epidemic size and overall financial burden of infection for all countries. However, data-driven research on global vaccine coordination in real-world human mobility networks is rare, particularly in the context of the COVID-19 pandemic with viral mutations.

**Fig. 1:**
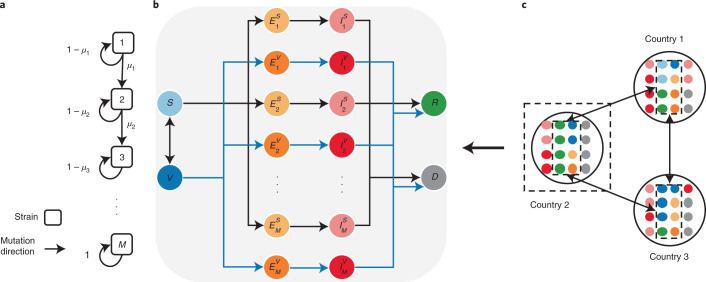
Illustration of the integrated mathematical model. **a**, The multistrain model. A linear strain space and local movement by a one-direction stepwise mutation are considered. *M* denotes the number of possible strains; *μ*_*m*_ denotes the mutation probability per infection. **b**, The SVEIRD model. Susceptible individuals (*S*) become vaccinated (*V*) at a vaccination rate determined by the global vaccine allocation strategy. Vaccinated individuals become susceptible after losing vaccinal immunity. Exposed individuals (${E}_{m}^{S}$ and ${E}_{m}^{V}$) are those infected by strain *m* and are divided into two classes, either with or without vaccinal immunity. Exposed individuals first become infectious (${I}_{m}^{S}$ and ${I}_{m}^{V}$) and then transition to either the recovered state (*R*) or the deceased state (*D*). For simplicity, we assume that co-infection is not possible and recovered individuals are immune to the disease. **c**, The SVEIRD-based metapopulation model. Due to travel restrictions, infectious and deceased individuals do not move between countries.

To address these challenges, we propose a multistrain metapopulation model to examine how the pandemic trajectory unfolds under different global vaccine allocation strategies. Harnessing real-world air traffic data, we construct a global mobility network to model human movement across countries. To model the emergence of new strains, we integrate the data-driven global metapopulation model with a multistrain model^35,36^, which captures the viral evolutionary dynamics and its effects on vaccine efficacy. By modelling the disparities in vaccination rates between HICs and LMICs, our model enables the analysis of the effectiveness of different global vaccine allocation strategies. We investigate the impact of viral evolution on epidemic dynamics in HICs and LMICs under equitable and inequitable global vaccine allocation strategies. We further evaluate the effects of different vaccine donation strategies to explore a practical pathway to global vaccine equity. We illustrate the model structure in Fig. 1. A detailed description of the model and the parameter settings is provided in the Methods.

## Results

### Vaccine inequity provides little benefit to HICs

**Fig. 2:**
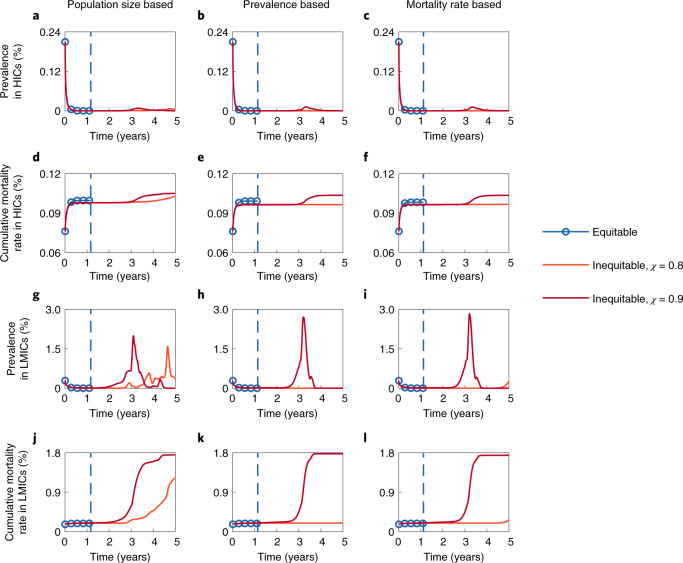
Impacts of equitable and inequitable vaccine allocation strategies on epidemic dynamics. **a**–**f**, Time series of the prevalence (**a**–**c**) and the cumulative mortality rate (**d**–**f**) in HICs under different global vaccine allocation strategies. **g**–**l**, Time series of the prevalence (**g**–**i**) and the cumulative mortality rate (**j**–**l**) in LMICs under different global vaccine allocation strategies. Three prioritization criteria for allocation are adopted: the population size (left), prevalence (middle) and mortality rate (right). The dashed lines in each panel indicate the time when the pandemic ends if the corresponding prioritization criterion is adopted (time exceeding five years is not presented). The dashed line and the solid line referring to the same global vaccine allocation strategy are represented by the same colour. The transmissibility and severity of each strain and the vaccine efficacy against each strain are shown in Supplementary Fig. 2. Parameter values: *M* = 5, *μ*_1_ = 5.6 × 10^−3^, *θ* = 0.2 and *λ* = 5 × 10^2^ (*λ* is the decrease rate of the probability of emergence of new and more dangerous strains). Source data

Figure 2 presents the time series of the prevalence (the fraction of infectious individuals in the population at each time step) and the cumulative mortality rate (the fraction of the cumulative count of deceased individuals in the population at each time step) under different global vaccine allocation strategies. Countries are classified as HICs or LMICs. Specifically, HICs include all high-income countries defined by the World Bank, plus China and Russia, because of their capability for mass production of COVID-19 vaccines. Under equitable global vaccine allocation strategies, available vaccine supplies will be equally allocated to all countries on the basis of the prioritization criteria, regardless of their wealth. Four prioritization criteria are considered: the population size, prevalence, mortality rate, and incidence (please refer to the Methods for the details). In inequitable global vaccine allocation strategies, however, at least a portion *χ* of daily available vaccines are purchased by HICs, and the remaining vaccines are allocated to LMICs (for both, the prioritization criteria are adopted).

We assume that (1) all current active cases are caused by strain 1 (calibrated by the epidemic data as of 15 June 2021), (2) the cumulative global vaccine supply will first increase exponentially until the maximum daily production capacity is reached in six months and then grow gradually at the maximum daily production capacity until the end of the pandemic, and (3) the cumulative global vaccine supply could fully vaccinate half of the world population in six months. Assumptions 2 and 3 are based on the prediction by Airfinity^37^, a health intelligence and analytics company. The results based on the prioritization criterion of incidence are similar to those based on prevalence and are shown in Supplementary Fig. 3.

We find that, in the first year, inequitable vaccine allocation strategies lead to a faster decline in incidence in HICs and a slower decline in LMICs, compared with the declines under equitable vaccine allocation strategies. The delay in vaccinations in LMICs not only leads to more infections in LMICs but also extends the duration of the pandemic globally. Under inequitable vaccine allocation strategies, we observe a rebound of cases in LMICs after the first year. Despite the short-term benefits, HICs are also vulnerable to reinfection, as the prevalence in HICs is shown to climb again in the subsequent waves (Supplementary Fig. 4). The onset of new waves of the disease in HICs is mainly caused by the higher probability of emerging new strains in LMICs. In the first four years, LMICs account for the majority of cases (Supplementary Fig. 5). Since each infection represents a chance of viral mutation, the probability of emerging new strains in LMICs is much higher than that in HICs. Importantly, a larger share of the global vaccine supply in inequitable allocation strategies (larger *χ*) results in little difference in mortality in HICs but results in a noticeable increase in LMICs. We observe similar results with various viral mutation parameters (Supplementary Figs. 6–14). Note that if an extremely transmissible strain evolves, vaccine inequity provides no benefits to HICs at all (for example, Supplementary Fig. 6).

### Prioritization criteria for global vaccine allocation

We compare the impacts of four prioritization criteria for global vaccine allocation. Although the four criteria lead to similar pandemic durations under equitable vaccine allocation strategies, there is a slight increase in prevalence and mortality worldwide when countries with larger population sizes are prioritized (Supplementary Fig. 15). Similarly, under inequitable vaccine allocation strategies, prioritizing countries with larger population sizes leads to earlier onset of new waves. However, it is worth noting that in the scenarios where an extremely transmissible strain emerges (for example, *M* = 6 and *θ* = 0.26, where θ is the increase in transmissibility of each new strain, in Supplementary Fig. 16), prioritizing countries with larger population sizes can reduce the overall prevalence in the long run. There is a notable trade-off between protecting the infected countries first and building up immunity in the larger, more susceptible populations. Our results indicate that the transmissibility of the virus plays the key role here. In most cases, we should prioritize vaccination in countries with higher incidence, prevalence or mortality by trying to contain the virus within these countries; however, in the rare case where the new strain is extremely transmissible, vaccination should be prioritized in densely populated countries to prepare for the global outbreak of the new strain (Supplementary Figs. 15–17).

### Vaccine inequity leads to the emergence of new strains

**Fig. 3:**
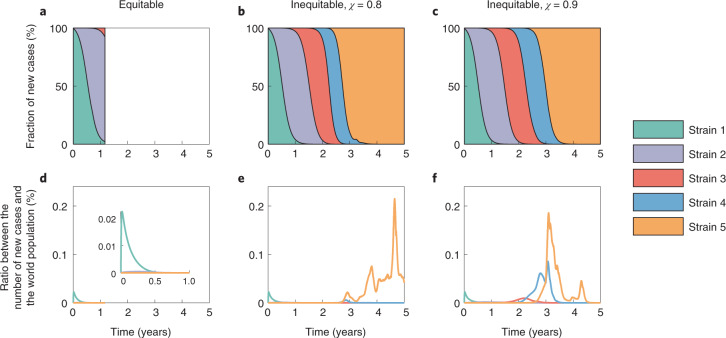
Emergence of new strains under equitable and inequitable vaccine allocation strategies. **a**–**c**, Area plots of the fraction of daily new cases produced by different strains. **d**–**f**, The ratio between the number of new cases produced by different strains and the world population. The plots are based on the equitable (left), inequitable and *χ* = 0.8 (middle), and inequitable and *χ* = 0.9 (right) vaccine allocation strategies. All results are based on the prioritization criterion of population size. The inset in **d** is a zoomed-in version of **d**. Parameter values: *M* = 5, *μ*_1_ = 5.6 × 10^−3^, *θ* = 0.2 and *λ* = 5 × 10^2^. Source data

We further investigate the fractions of new cases resulting from different strains under equitable and inequitable vaccine allocation strategies (Fig. 3). As an example, we present the results based on the prioritization criterion of population size. We find that equitable vaccine allocation strategies substantially curb the spread of new strains. Due to viral mutations, new strains with higher transmissibility take over the world and are responsible for the majority of new cases in both HICs and LMICs. A slight increase in the incidence of the previous strains elevates the risk of outbreak of new strains. In addition, a larger share of the global vaccine supply for HICs (larger *χ*) results in earlier peaks for all emerged strains. Except for strain 1, the fraction of new cases produced by other strains in LMICs is much higher than that in HICs (Supplementary Figs. 18 and 19). These results further highlight that the short-term benefits to HICs under inequitable vaccine allocation are limited and come at the sacrifice of running a much higher risk of new strains’ outbreaks, eventually leading to unnecessary deaths in not only LMICs but also HICs. Note that, although equitable vaccine allocation strategies result in fewer infections and deaths globally, this may not always be the optimal decision for HICs. If the virus reaches its peak fitness sooner and stops mutating into more transmissible strains, HICs continue to benefit slightly more from vaccine inequity in the short term. However, the local epidemic cannot be fully ended due to continuous imported cases. This particular scenario is in line with a recent game-theoretical analysis^9^. However, we illustrate in the following that HICs can further improve their benefits by donating excess vaccines to LMICs when the local epidemic in HICs is under control.

### Vaccine donation is a practical pathway to vaccine equity

**Fig. 4:**
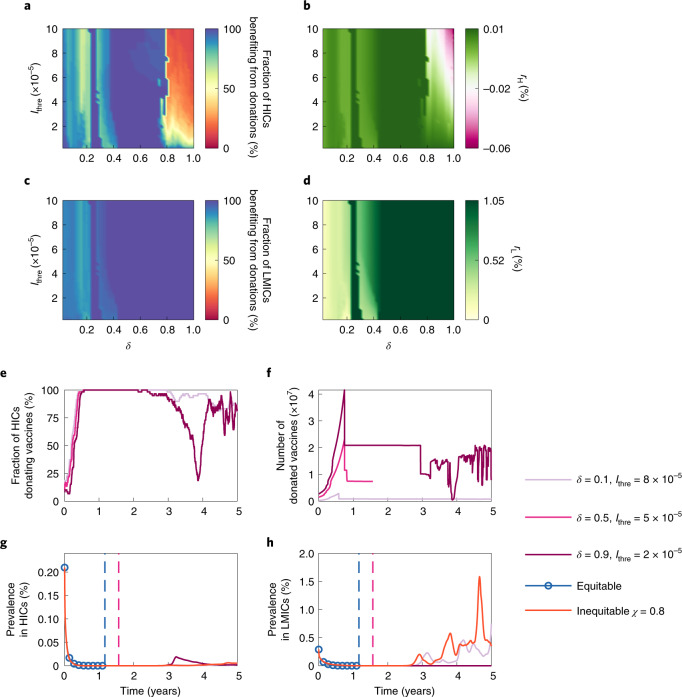
Impacts of different allow-donation vaccine allocation strategies on epidemic dynamics. **a**,**c**, Fraction of HICs (**a**) and LMICs (**c**) benefiting from donations. **b**,**d**, Average lives saved by vaccine donations as the share of the national population in HICs (*r*_*H*_, **b**) and LMICs (*r*_*L*_, **d**). Please refer to the Methods for the details of *r*_*H*_ and *r*_*L*_. **e**, Fraction of HICs donating vaccines. **f**, Total number of donated vaccines. **g**,**h**, Prevalence in HICs (**g**) and LMICs (**h**) under different vaccine allocation strategies. The dashed lines indicate the time when the pandemic ends. Countries with larger population sizes are prioritized for vaccination. Parameter values: *M* = 5, *μ*_1_ = 5.6 × 10^−3^, *θ* = 0.2 and *λ* = 5 × 10^2^. Source data

We assume that an HIC adopts an allow-donation vaccine allocation strategy as follows: it will denote a certain portion (denoted by *δ*) of its vaccine supplies to international facilities, such as COVAX, as long as the prevalence in its population is less than a certain threshold (denoted by *I*_thre_). These vaccines will be equitably allocated to all LMICs on the basis of the prioritization criteria. We explore different allow-donation vaccine allocation strategies (that is, different combinations of *δ* and *I*_thre_) to examine their impact on epidemic dynamics in Fig. 4. Here we focus on the scenario where *χ* = 0.8 and countries with larger population sizes are prioritized for vaccination (the prioritization criterion currently adopted by COVAX). Countries benefiting from donations are those with a lower mortality through adopting the allow-donation vaccine allocation strategies.

Unsurprisingly, almost all LMICs benefit from vaccine donations regardless of when and how many vaccines are donated by HICs (Fig. 4c). More vaccines donated by HICs result in a larger reduction in the cumulative mortality in LMICs (Fig. 4d). The reduction in cumulative mortality in LMICs is more sensitive to the number of vaccines donated by HICs than to when HICs start donations. A small increase in vaccine donations results in a larger decrease in cumulative mortality in LMICs. Such decreases become significant only when the portion of vaccines denoted by HICs reaches a certain level (around 46%). For HICs, donating more vaccines brings higher benefits before a certain portion (80%) is reached (as shown in Fig. 4a,b). These results indicate that vaccine donations by HICs could protect both HICs and LMICs. It is in HICs’ rational self-interests to share vaccines with LMICs before vaccinating their entire population. The results with various viral mutation parameters (Supplementary Figs. 20–24) are consistent with those in Fig. 4.

Figure 4e–h illustrates the impacts of three representative allow-donation vaccine allocation strategies on epidemic dynamics. The values *δ* = 0.1 and *I*_thre_ = 8 × 10^−5^ represent a scenario where HICs donate a small portion of vaccines to LMICs although the fraction of infected cases in their own countries is relatively high. The values *δ* = 0.9 and *I*_thre_ = 2 × 10^−5^ represent a scenario where HICs donate a large portion of vaccines to LMICs only when the fraction of infected cases in their own countries is low. The values *δ* = 0.5 and *I*_thre_ = 5 × 10^−5^ represent a scenario where HICs donate a moderate portion of vaccines to LMICs only when the fraction of infected cases in their own countries is relatively low. For the first year, the difference in the fraction of HICs donating vaccines is small under the three allow-donation vaccine allocation strategies (Fig. 4e). The difference in the count of vaccines donated to LMICs (Fig. 4f) is more obvious, which explains why the reduction in cumulative mortality in LMICs is more sensitive to the number of vaccines donated by HICs than to when HICs start donations. Among the three strategies, the one where *δ* = 0.5 and *I*_thre_ = 5 × 10^−5^ leads to the best pandemic outcome, where the pandemic ends the earliest (only second to the fully equitable vaccine allocation strategy, which is hard to achieve). Compared with the scenario where *δ* = 0.9 and *I*_thre_ = 2 × 10^−5^, the new wave that appears in HICs two years later has a much smaller size than that in the scenario where *δ* = 0.1 and *I*_thre_ = 8 × 10^−5^. This indicates that HICs should donate a small portion of vaccines to LMICs even when the number of infected cases is high locally, rather than waiting for the local epidemic to be fully controlled.

### Donating vaccines only to neighbours has limited effects

**Fig. 5:**
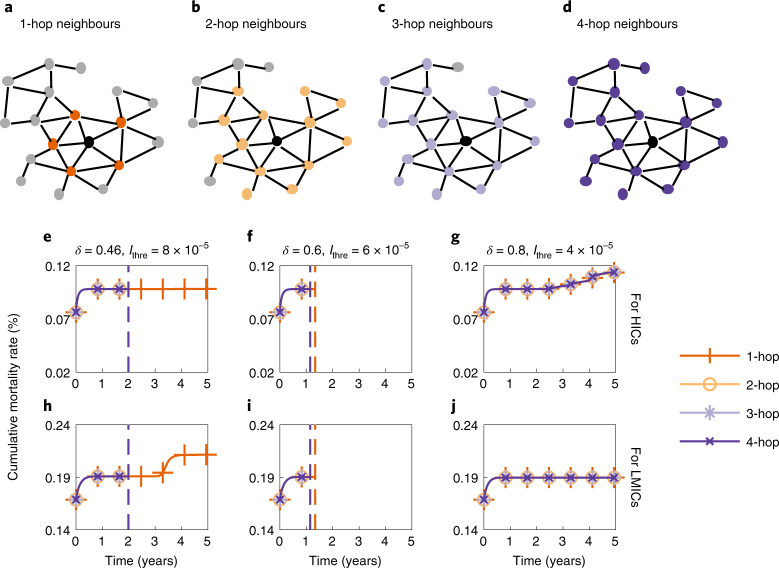
Impact of donating vaccines to neighbouring LMICs. **a**–**d**, A country (the black node) and its 1-hop (**a**), 2-hop (**b**), 3-hop (**c**) and 4-hop (**d**) neighbours on the global mobility network (Supplementary Fig. 27) constructed on the basis of the air traffic data. **e**–**j**, Cumulative mortality rate in HICs (**e**–**g**) and LMICs (**h**–**j**) over time if HICs donate vaccines to only their 1-hop, 2-hop, 3-hop and 4-hop LMIC neighbours under scenarios where *δ* = 0.46 and *I*_thre_ = 8 × 10^−5^ (**e** and **h**), *δ* = 0.6 and *I*_thre_ = 6 × 10^−5^ (**f** and **i**), and *δ* = 0.8 and *I*_thre_ = 4 × 10^−5^ (**g** and **j**). The dashed lines indicate the time when the pandemic ends. Countries with larger population sizes are prioritized for vaccination. Parameter values: *M* = 5, *μ*_1_ = 5.6 × 10^−3^, *θ* = 0.2 and *λ* = 5 × 10^2^. Source data

We further investigate the effects of vaccine donations if HICs donate vaccines to only their neighbouring LMICs (in the hope of reducing the risk of infected cases arriving from neighbouring LMICs) in Fig. 5. We consider three vaccine donation scenarios benefiting both HICs and LMICs based on the results in Fig. 4: *δ* = 0.46 and *I*_thre_ = 8 × 10^−5^, *δ* = 0.6 and *I*_thre_ = 6 × 10^−5^, and *δ* = 0.8 and *I*_thre_ = 4 × 10^−5^. ‘Neighbours’ of a country are defined on the basis of the global mobility network derived from the real-world air traffic data from the Official Aviation Guide (https://www.oag.com/). A node represents a country or region. The existence of an edge between two countries or regions represents the existence of direct flights between them. We define four kinds of neighbours of a node: 1-hop, 2-hop, 3-hop and 4-hop neighbours. Here, *k*-hop neighbours of a target node refer to nodes that are at most *k* hops away from the target node^38^. Thus, 1-hop neighbours (generally called neighbouring countries) are countries that are reachable via direct flights; 4-hop neighbours contain all LMICs. Graphic illustrations of *k*-hop neighbours of a target country are provided in Fig. 5a–d. As Fig. 5h–j indicates, there is little difference in the cumulative mortality in LMICs under the 2-hop, 3-hop and 4-hop scenarios. However, if HICs donate vaccines to only their 1-hop (immediate) neighbours, there may be a noticeable increase in the cumulative mortality in LMICs (Fig. 5h). Although there is little difference in the cumulative mortality in HICs under different scenarios (Fig. 5e–g), donating vaccines to a larger proportion of LMICs rather than only neighbouring LMICs can lead to an earlier end of the pandemic.

## Discussion

Knowing the impact of global COVID-19 vaccine allocation on both LMICs and HICs is crucial for controlling the COVID-19 pandemic. We propose a multistrain metapopulation model to investigate the short-term and long-term impacts of vaccine equity, accounting for viral mutations and global human mobility. The results show that vaccine inequity provides only limited and short-term benefits to HICs, whereas it leads to moderate increases in infections and deaths in LMICs. However, such increases may result in elevated risk of future waves (caused by new strains) affecting not only LMICs but also HICs. A sharper disparity in vaccine allocation between HICs and LMICs leads to earlier and larger peaks in pandemic size in future waves.

However, we found that, as vaccines are still limited currently, if HICs donate a certain portion of vaccine supplies to LMICs instead of vaccinating their entire population as the top priority, enormous public health benefits can be seen for both HICs and LMICs. Furthermore, for HICs, donating a small portion of vaccines to LMICs could better lower the risk of future waves than waiting for the epidemic to be controlled in their own countries. Additionally, donating vaccines to more LMICs rather than only directly neighbouring LMICs is more efficient in curbing the spread of the virus.

We further investigate the distribution of cases caused by different strains under equitable and inequitable global vaccine allocation strategies. The results show that equitable global vaccine allocation strategies substantially curb the spread of new strains globally. Assuming that fully vaccinated individuals can be vaccinated again after losing vaccine-induced immunity, we found that prioritizing vaccination for countries with higher prevalence, higher incidence or higher mortality rates results in similar pandemic outcomes less severe than those under the population-based proportional allocation strategies. This is probably because smaller outbreaks lead to fewer immune-escape variants, under our simulation. Many developing countries, which are currently facing high prevalence or incidence, still lack vaccines. Having the third dose certainly improves the effectiveness of the vaccine, but the improvement is less than that of the first vaccination, especially for those countries that need vaccines to lower their high prevalence, incidence or mortality rates.

Our research has limitations. First, although we derive a country-specific severity matrix to model the heterogeneous age structures across different countries, the model is not stratified by age within each country. The difference in age structures results in heterogeneous infection fatality rates and heterogeneous susceptibility to infection^39–42^. Due to limited data on the susceptibility to infection among different age groups and the lack of age-mixing patterns for different countries, we do not parameterize an age-stratified model for each country. The model can be easily calibrated if such data are available. Second, according to the finding that reinfections are uncommon in the general population^43–46^, we investigate the lifelong and different short-lived natural immunity settings. Sensitivity analysis (Supplementary Figs. 25 and 26) shows that, if natural immunity is short-lived, global vaccine inequity provides even smaller benefits to HICs. Future research should incorporate more realistic natural immunity duration data for different strains (if available). Third, we characterize the viral evolutionary dynamics as a simple multistrain model with a linear strain space, where the severity of new strains always increases. However, selection against severe strains would happen as the virus evolves^47^. Expanding this model to include the selection of strains in a multidimensional antigenic space is an important topic for further exploration when more data about different strains become available.

In general, our results provide data-driven numerical evidence that it is short-sighted for HICs to hoard global vaccine supplies and leave LMICs behind. The long-term impact of vaccination is often neglected relative to short-term and immediate convincing consequences. However, global human movement speeds up the spread of new variants around the world. We have seen how highly contagious strains were generated in a population within a single epidemic and then spread to the rest of the world^17–21^. Long-term disease-protection benefits can be obtained through global cooperation. Our results thus highlight the importance of assessing the long-term effect of vaccination on the emergence of new strains and making better global allocation strategies. Currently, many HICs have secured sufficient vaccine doses to vaccinate their entire population several times. Making immediate and more generous donations to LMICs is a practical pathway to achieving global equity because it offers a big win–win for both HICs and LMICs.

## Methods

### Multistrain model

The SARS-CoV-2 virus is mutating over time, resulting in genetic variation over the course of the pandemic. On the basis of virology studies^48^, we assume that SARS-CoV-2 mutates rapidly, but most of the mutations are neutral. Neutral mutations have little impact on the virus’s ability to cause infections. Some mutations at critical locations of the genetic sequence may change the virus’s transmissibility and severity. Here we extend the multiple-strain model^35,36,41^ to characterize viral evolutionary dynamics. For country *i*, such dynamics are captured by the transmissibility matrix ${{{\mathcal{T}}}}$, the severity matrix ${{{{\mathcal{F}}}}}_{i}$ and the mutation matrix ${{{\mathcal{U}}}}$, all with dimensions *M* × *M*. *M* ≥ 2 represents the number of possible strains. The transmissibility matrix ${{{\mathcal{T}}}}$ and the severity matrix ${{{{\mathcal{F}}}}}_{i}$ are both diagonal matrices. ${{{{\mathcal{T}}}}}_{m}$ and ${{{{\mathcal{F}}}}}_{i,m}$ represent the transmissibility (the probability of disease transmission in a single contact multiplied by the average number of contacts per person per unit time; determined by the basic reproduction number and the infectious period) and the severity (the infection fatality rate) of strain *m* ∈ {1, 2, . . . , *M*}, respectively. We set a country-specific severity matrix to account for the heterogeneity in the health-care burden of COVID-19 and the age structure in different countries (Supplementary Note 3)^41,49,50^. We assume a linear strain space and local movement by a one-direction stepwise mutation in the model^47^. Each point in the strain space represents a single strain. More similar strains are closer in the strain space. The virus in strain *m* either remains as strain *m* with probability 1 − *μ*_*m*_ or mutates to strain *m* + 1 (one-direction stepwise mutation) with probability *μ*_*m*_ while adapting to a new host (please refer to Supplementary Note 4 for the details of the spreading process). Thus, we construct ${{{\mathcal{U}}}}$ as

1 $${{{\mathcal{U}}}}=\left(\begin{array}{lllll}1-{\mu }_{1}&{\mu }_{1}&0&\cdots \ &0\\ 0&1-{\mu }_{2}&{\mu }_{2}&\cdots \ &0\\ \vdots &\vdots &\vdots &\ddots &\vdots \\ 0&0&0&\cdots \ &1\end{array}\right).$$

The virus cannot evolve indefinitely, primarily because each nucleotide can only mutate to three others (for example, adenine can only mutate to thymine or guanine or cytosine), and the genome of SARS-CoV-2 has a limited number of nucleotides^51,52^. As the virus evolves in the strain space, the probability of major new changes per infection decreases because fewer possible genome sequences remain. Thus, we assume that *μ*_*m*+1_ = *μ*_*m*_/*λ*. Since detected cases of reinfection are much rarer than postvaccination cases^53^, we assume that (1) hosts recovered from either strain are immune to all other strains, and (2) hosts vaccinated for one strain exhibit a certain degree of cross-protection from other strains. The cross-immunity between the vaccine strain *m* and a mutant strain *n* is given by ${{\mathrm{e}}}^{-{(\frac{m-n}{d})}^{2}}$, the chance that the immunity induced by vaccines for strain *m* will provide immunity to strain *n*^47,54^. Here, ∣*m* − *n*∣ represents the antigenic distance, and *d* is a fixed value representing the distance of antigenicity between the vaccine strain and a mutant strain when the cross-immunity is reduced to 1/e. This expression is based on the assumption that vaccine-induced immunity is less cross-reactive as the antigenic distance between the vaccine strain and a mutant strain increases. Suppose vaccines designed for strain *m* demonstrate an efficacy of *η*_*mn*_ and *ϵ*_*mn*_ against infection and death from strain *n*, respectively. Then, we define

2 $$\begin{array}{l}{\eta }_{mn}={\eta }_{mm}{{\mathrm{e}}}^{-{(\frac{m-n}{d})}^{2}},\\ {\epsilon }_{mn}={\epsilon }_{mm}{{\mathrm{e}}}^{-{(\frac{m-n}{d})}^{2}}.\end{array}$$

Here we assume that all current cases are caused by strain 1, and only vaccines designed for strain 1 are provided. Thus, by taking *m* = 1, equation (2) can be simplified as

3 $$\begin{array}{l}{\eta }_{n}={\eta }_{1}{{\mathrm{e}}}^{-{(\frac{n-1}{d})}^{2}},\\ {\epsilon }_{n}={\epsilon }_{1}{{\mathrm{e}}}^{-{(\frac{n-1}{d})}^{2}},\end{array}$$

where *η*_*n*_ and *ϵ*_*n*_ represent vaccine efficacy against infection and death from strain *n*, respectively. We have already seen the emergence of more infectious and more harmful strains in the real world^17–20^. For example, the hazard of death associated with the B.1.1.7 (Alpha) strain is estimated as 61% higher than with pre-existing variants, and the basic reproduction number of B.1.617.2 (Delta) is estimated at about 5–8, which is much higher than that of the original strain (2.79; ref. ^55^). We thus incorporate these facts in our model by assuming that ${{{{\mathcal{T}}}}}_{1} < {{{{\mathcal{T}}}}}_{2} < ... < {{{{\mathcal{T}}}}}_{M}$, since the stability of the virus increases (specifically, we assume that ${{{{\mathcal{T}}}}}_{n+1}=(1+\theta ){{{{\mathcal{T}}}}}_{n}$); and that ${{{{\mathcal{F}}}}}_{i,n+1}$ is correlated with ${{{{\mathcal{T}}}}}_{n+1}(1-{\eta }_{n+1})$, which is proportional to the viral load. Thus, ${{{{\mathcal{F}}}}}_{i,1} < {{{{\mathcal{F}}}}}_{i,2} < ... < {{{{\mathcal{F}}}}}_{i,M}$.

### SVEIRD-based metapopulation model

The proposed SVEIRD-based metapopulation model extends the classic susceptible–exposed–infectious–recovered model to account for the effects of viral mutations, vaccine updates, non-pharmaceutical interventions (NPIs) and restricted international mobility. Individuals in country *i* (with population size *N*_*i*_) are divided into the following classes: susceptible individuals (*S*_*i*_), vaccinated individuals (*V*_*i*_), individuals exposed to strain *m* (not yet infectious) without vaccinal immunity (${E}_{i,m}^{S}$), individuals exposed to strain *m* (not yet infectious) with vaccinal immunity (${E}_{i,m}^{V}$), infectious individuals caused by strain *m* without vaccinal immunity (${I}_{i,m}^{S}$), infectious individuals caused by strain *m* with vaccinal immunity (${I}_{i,m}^{V}$), recovered individuals (*R*_*i*_) and deceased individuals (*D*_*i*_). Susceptible individuals are vaccinated at the vaccination rate *ϕ*_*i*_(*t*) according to the global vaccine allocation strategy. We assume that vaccinated individuals gradually lose vaccinal immunity and become fully susceptible again at the rate *ε*. We demonstrate the contacts between susceptible individuals (vaccinated individuals) and infectious individuals as a diagonal matrix ${{{{\mathcal{C}}}}}_{i}^{S}(t)$ (${{{{\mathcal{C}}}}}_{i}^{V}(t)$) with dimensions *M* × *M* for country *i* at time *t*. The term ${{{{\mathcal{C}}}}}_{i}^{S}{(t)}_{m}$ represents the number of contacts between susceptible individuals and infectious individuals caused by strain *m* for country *i* at time *t*:

4 $${{{{\mathcal{C}}}}}_{i}^{S}{(t)}_{m}=(1-{c}_{i})\frac{{I}_{i,m}^{S}(t)+{I}_{i,m}^{V}(t)}{{N}_{i}}{S}_{i}(t),$$

where *c*_*i*_ ∈ [0, 1] quantifies the effectiveness of NPIs for country *i*. Similarly:

5 $${{{{\mathcal{C}}}}}_{i}^{V}{(t)}_{m}=(1-{c}_{i})\frac{{I}_{i,m}^{S}(t)+{I}_{i,m}^{V}(t)}{{N}_{i}}{V}_{i}(t).$$

Thus, the transition rate from *S*_*i*_ to ${E}_{i,m}^{S}$ is ${\sum }_{n}{[{{{{\mathcal{C}}}}}_{i}^{S}(t){{{\mathcal{T}}}}{{{\mathcal{U}}}}]}_{n,m}$. Similarly, the transition rate from *V*_*i*_ to ${E}_{i,m}^{V}$ is ${\sum }_{n}(1-{\eta }_{n}){[{{{{\mathcal{C}}}}}_{i}^{V}(t){{{\mathcal{T}}}}{{{\mathcal{U}}}}]}_{n,m}$. Exposed individuals become infectious with transition rate *σ*; thus, the incubation period is 1/*σ*.

To model how countries introduce and relax NPIs during the COVID-19 pandemic, we consider the reproduction-number-based adaptive policy adoption strategy^56–58^, where more stringent NPIs are triggered when the local effective reproduction number exceeds a certain threshold, and NPIs are relaxed to less stringent when it falls below the threshold. Note that each country introduces or relaxes NPIs on the basis of the local effective reproduction number within each country, instead of the effective reproduction number for the metapopulation network^59–61^. The details of the adaptive policy adoption strategy can be found in Supplementary Note 2.

Infectious individuals become either recovered or deceased at transition rate *α*; thus, the infectious period is 1/*α*. For individuals without vaccinal immunity, the transition rates from infectious (caused by strain *m*) to recovered and deceased are $(1-{{{{\mathcal{F}}}}}_{i,m})\alpha$ and ${{{{\mathcal{F}}}}}_{i,m}\alpha$, respectively; for individuals with vaccinal immunity, the transition rates from infectious (caused by strain *m*) to recovered and deceased are $[1-(1-{\epsilon }_{m}){{{{\mathcal{F}}}}}_{i,m}]\alpha$ and $(1-{\epsilon }_{m}){{{{\mathcal{F}}}}}_{i,m}\alpha$, respectively. Individuals recovered from either strain are assumed to be immune to all other strains. We assume that infectious and deceased individuals do not travel across countries. *G*_*ij*_(*t*) denotes the number of individuals travelling from country *i* to country *j* at time *t*. For simplicity, we assume a dynamic and undirected international mobility network in this model and thus define *G*_*ij*_(*t*) as

6 $${G}_{ij}(t)={G}_{ji}(t)=\gamma [{A}_{i}(t){P}_{ij}+{A}_{j}(t){P}_{ji}].$$

Here, *γ* is the average mobility rate, ${A}_{i}(t)={N}_{i}-{D}_{i}(t)-{\sum }_{m}[{I}_{i,m}^{S}(t)+{I}_{i,m}^{V}(t)]$ is the number of individuals allowed to travel from country *i* to other countries at time *t*, and *P*_*ij*_ is the fraction of individuals travelling from country *i* to country *j* and is assumed to be constant. ∑_*j*_*P*_*ij*_ = 1, and *P*_*ii*_ = 0. We denote *F*_*ij*_ as the number of passengers travelling from country *i* to country *j* per day, and *F*_*i*_ = ∑_*j*_*F*_*ij*_. Then, *P*_*ij*_ = *F*_*ij*_/*F*_*i*_. We obtain *F*_*ij*_ by averaging the aggregated number of seats on scheduled commercial flights between countries per day in the year 2020 (Supplementary Note 6). All possible state transitions within each country are shown in Fig. 1. The disease transmission dynamics is described by the following equations:

7 $$\begin{array}{rcl}{\partial }_{t}{S}_{i}(t)&=&-{\phi }_{i}(t)-\mathop{\sum}\limits_{m}\mathop{\sum}\limits_{n}{\left[{{{{\mathcal{C}}}}}_{i}^{S}(t){{{\mathcal{T}}}}{{{\mathcal{U}}}}\right]}_{n,m}+\varepsilon {V}_{i}(t)\\&&+\mathop{\sum}\limits_{j}{G}_{ij}(t)\left[\frac{{S}_{j}(t)}{{A}_{j}(t)}-\frac{{S}_{i}(t)}{{A}_{i}(t)}\right],\\ {\partial }_{t}{V}_{i}(t)&=&{\phi }_{i}(t)-\mathop{\sum}\limits_{m}\mathop{\sum}\limits_{n}(1-{\eta }_{n}){\left[{{{{\mathcal{C}}}}}_{i}^{V}(t){{{\mathcal{T}}}}{{{\mathcal{U}}}}\right]}_{n,m}-\varepsilon {V}_{i}(t)\\&&+\mathop{\sum}\limits_{j}{G}_{ij}(t)\left[\frac{{V}_{j}(t)}{{A}_{j}(t)}-\frac{{V}_{i}(t)}{{A}_{i}(t)}\right],\\ {\partial }_{t}{E}_{i,m}^{S}(t)&=&\mathop{\sum}\limits_{n}{\left[{{{{\mathcal{C}}}}}_{i}^{S}(t){{{\mathcal{T}}}}{{{\mathcal{U}}}}\right]}_{n,m}-\sigma {E}_{i,m}^{S}(t)\\&&+\mathop{\sum}\limits_{j}{G}_{ij}(t)\left[\frac{{E}_{j,m}^{S}(t)}{{A}_{j}(t)}-\frac{{E}_{i,m}^{S}(t)}{{A}_{i}(t)}\right],\\ {\partial }_{t}{E}_{i,m}^{V}(t)&=&\mathop{\sum}\limits_{n}(1-{\eta }_{n}){\left[{{{{\mathcal{C}}}}}_{i}^{V}(t){{{\mathcal{T}}}}{{{\mathcal{U}}}}\right]}_{n,m}-\sigma {E}_{i,m}^{V}(t)\\&&+\mathop{\sum}\limits_{j}{G}_{ij}(t)\left[\frac{{E}_{j,m}^{V}(t)}{{A}_{j}(t)}-\frac{{E}_{i,m}^{V}(t)}{{A}_{i}(t)}\right],\\ {\partial }_{t}{I}_{i,m}^{S}(t)&=&\sigma {E}_{i,m}^{S}(t)-\alpha {I}_{i,m}^{S}(t), {\partial }_{t}{I}_{i,m}^{V}(t)=\sigma {E}_{i,m}^{V}(t)-\alpha {I}_{i,m}^{V}(t),\\ {\partial }_{t}{R}_{i}(t)&=&\mathop{\sum}\limits_{m}(1-{{{{\mathcal{F}}}}}_{i,m})\alpha {I}_{i,m}^{S}(t)+\mathop{\sum}\limits_{m}\left[1-(1-{\epsilon }_{m}){{{{\mathcal{F}}}}}_{i,m}\right]\alpha {I}_{i,m}^{V}(t)\\&&+\mathop{\sum}\limits_{j}{G}_{ij}(t)\left[\frac{{R}_{j}(t)}{{A}_{j}(t)}-\frac{{R}_{i}(t)}{{A}_{i}(t)}\right],\\ {\partial }_{t}{D}_{i}(t)&=&\mathop{\sum}\limits_{m}{{{{\mathcal{F}}}}}_{i,m}\alpha {I}_{i,m}^{S}(t)+\mathop{\sum}\limits_{m}(1-{\epsilon }_{m}){{{{\mathcal{F}}}}}_{i,m}\alpha {I}_{i,m}^{V}(t).\end{array}$$

### Global vaccine allocation model

We denote the cumulative global vaccine supply at time *t* as *φ*(*t*). On the basis of the predicted exponential growth in the global vaccine supply in the coming months^37^, we assume that *φ*(*t*) first increases exponentially until the maximum daily production capacity is reached at time *τ* and then grows gradually at the maximum daily production capacity *φ*(*τ*) − *φ*(*τ* − 1) until the end of the epidemic:

8 $$\varphi (t)=\left\{\begin{array}{ll}\varphi (0){(1+v)}^{t}&t\le \tau ,\\ \varphi (\tau )+(t-\tau )\times [\varphi (\tau )-\varphi (\tau -1)]&\,{{\mbox{otherwise}}}\,.\end{array}\right.$$

The parameter *v* quantifies how fast the manufacturers worldwide are ramping up the production of vaccines. The global supply of vaccines at time *t* is *φ*(*t* + 1) − *φ*(*t*). The number of vaccines allocated to each country depends on the global supply of vaccines, the global vaccine allocation strategy and the demand for vaccines for each country. Here we model two sets of global vaccine allocation strategies: equitable and inequitable ones. Under equitable vaccine allocation strategies, available vaccines at each time will be allocated to each country on the basis of the prioritization criteria. In inequitable vaccine allocation strategies, a minimum fraction *χ* of vaccines available at each time are purchased by HICs, and the remaining vaccines are allocated to LMICs. Then, in both the HIC and LMIC groups, vaccines are allocated to each country on the basis of the prioritization criteria. Countries are classified into HICs or LMICs according to their incomes and their capability for mass production of COVID-19 vaccines. The basic income classification is based on the gross national income per capita (calculated using the World Bank Atlas method in US dollars). We summarize the basic income classification results in Table 1. Specifically, HICs in our model include high-income countries in Table 1 plus China and Russia, because of their capability for mass production of COVID-19 vaccines. The remaining countries are defined as LMICs. We consider four prioritization criteria in both equitable and inequitable vaccine allocation strategies:

- Population size: Priority to countries with larger population sizes.
- Prevalence: Priority to countries with a higher number of active cases (currently infectious cases) per capita.
- Mortality rate: Priority to countries with a higher number of new deaths during the past two weeks as a share of the total population.
- Incidence: Priority to countries with a higher number of new cases during the past two weeks as a share of the total population.

All prioritization criteria are being updated dynamically on the basis of the epidemic evolution within each country (Supplementary Note 5). Currently, some vaccines require two doses to provide broader and longer-lasting immunity against the virus, such as Pfizer-BioNTech and Moderna^62^, while some vaccines need only one dose, such as Johnson & Johnson’s vaccines. For simplicity, we assume that (1) all vaccines are administered with a two-dose schedule, (2) two doses are administered simultaneously, (3) the body can build full vaccinal immunity immediately after vaccination and (4) the upper bounds of daily vaccination rates for HICs and LMICs are the maximum daily vaccination rates achieved by HICs and LMICs from 1 December 2020 to 15 June 2021 (*t* = 0).

**Table 1:** Basic income classification based on the gross national income per capita (calculated using the World Bank Atlas method in US dollars)

Class	Number of countries	Total population
High-income countries	57 (31.8%)	1,238,673,312 (16.2%)
Upper-middle-income countries	48 (26.8%)	2,846,563,032 (37.2%)
Lower-middle-income countries	50 (27.9%)	2,984,937,312 (39.0%)
Low-income countries	24 (13.4%)	590,387,508 (7.7%)

### Calculation of the average lives saved by vaccine donations

We denote ${\overline{D}}_{i,{\mathrm{ineq}}}$ and ${\overline{D}}_{i,{\mathrm{don}}}$ as the cumulative mortality for country *i* at the end of the simulation under inequitable and allow-donation vaccine allocation strategies, respectively. The average lives saved by vaccine donations (the difference between the cumulative mortality under the allow-donation allocation strategy and that under the inequitable allocation strategy) as the share of the national population in HICs and LMICs are denoted by *r*_*H*_ and *r*_*L*_, respectively:

9 $$\begin{array}{l}{r}_{H}=\frac{1}{| H| }\mathop{\sum}\limits_{i\in H}\frac{{\overline{D}}_{i,{\mathrm{ineq}}}-{\overline{D}}_{i,{\mathrm{don}}}}{{N}_{i}},\\ {r}_{L}=\frac{1}{| L| }\mathop{\sum}\limits_{i\in L}\frac{{\overline{D}}_{i,{\mathrm{ineq}}}-{\overline{D}}_{i,{\mathrm{don}}}}{{N}_{i}}.\end{array}$$

Here, *H* and *L* denote the set of HICs and LMICs, respectively; and ∣*H*∣ and ∣*L*∣ denote the number of HICs and LMICs, respectively.

### Model initialization

In all simulations, *t* = 0 corresponds to 15 June 2021. We roughly take ${E}_{i,m}^{S}(0)=0$, ${E}_{i,m}^{V}(0)=0$, ${I}_{i,m}^{V}(0)=0$ and ${I}_{i,m}^{S}(0)=0$ (*m* ≠ 1). *V*_*i*_(0) is estimated by the number of individuals that are fully vaccinated at *t* = 0. ${I}_{i,1}^{S}(0)$ is estimated by the number of currently active cases at *t* = 0. *R*_*i*_(0) and *D*_*i*_(0) are estimated by the cumulative numbers of recovered and decreased individuals at *t* = 0. Thus:

10 $$\begin{array}{l}{S}_{i}(0)={N}_{i}-{V}_{i}(0)-{I}_{i,1}^{S}(0)-{R}_{i}(0)-{D}_{i}(0),\\ \varphi (0)=2\mathop{\sum}\limits_{i}{V}_{i}(0).\end{array}$$

Due to severe under-reporting worldwide, especially in LMICs with low testing rates, we adopt the probabilistic bias analysis^63,64^ to correct the numbers of infections, recoveries, deaths and active cases for all countries. Please refer to Supplementary Note 1 for a detailed description of the data correction.

### Reporting Summary

Further information on research design is available in the Nature Research Reporting Summary linked to this article.

## Supplementary information

Supplementary Information Supplementary Notes 1–6, Figs. 1–32, Tables 1–5 and Equations 1–6.

Reporting Summary

Peer Review Information

## Data Availability

The country-level epidemiological data were collected from publicly available repositories operated by the Johns Hopkins University Center for Systems Science and Engineering (https://github.com/CSSEGISandData/COVID-19) and Our World in Data (https://github.com/owid/covid-19-data). The country-level income data were obtained from the World Bank (https://data.worldbank.org). The population data were obtained from the United Nations World Population Prospects national estimates (https://population.un.org/wpp/). The global mobility data are commercially available from the Official Aviation Guide (https://www.oag.com/) and are used under licence for the current study. Due to restrictions in the licensing agreement with the Official Aviation Guide, these data are not publicly available. The other data we used are documented in the main text. The processed data have been deposited in Zenodo (10.5281/zenodo.5810400). Source data are provided with this paper.

## Source data

Source Data Fig. 2 Statistical source data.
Source Data Fig. 3 Statistical source data.
Source Data Fig. 4 Statistical source data.
Source Data Fig. 5 Statistical source data.

## Code availability

All code used to generate the findings of this study has been deposited in Zenodo (10.5281/zenodo.5810400).
