# Supplementary material for: Equitable access to COVID-19 vaccines makes a life-saving difference to all countries
Source: Nat Hum Behav. 2022 Jan 31;6(2):207–16. doi: 10.1038/s41562-022-01289-8 (PMC8873023; doi:10.1038/s41562-022-01289-8)
Supplement: Supplementary file 1 — Supplementary Notes 1–6, Figs. 1–32, Tables 1–5 and Equations 1–6. [file 41562_2022_1289_MOESM1_ESM.pdf]

nature portfolio

https://doi.org/10.1038/s41562-022-01289-8

Supplementary information

Equitable access to COVID-19 vaccines makes a life-saving difference to all countries

In the format provided by the authors and unedited

Supplementary Information for

Equitable access to COVID-19 vaccines makes a life-saving difference to all countries

Yang Ye[1], Qingpeng Zhang[1, *], Xuan Wei[2], Zhidong Cao[3, 4], Hsiang-Yu Yuan[5, 6], and Daniel Dajun Zeng[3, 4, *]

[1]School of Data Science, City University of Hong Kong, Hong Kong SAR, China.
[2]Antai College of Economics and Management, Shanghai Jiao Tong University, Shanghai, China.
[3]The State Key Laboratory of Management and Control for Complex Systems, Institute of Automation, Chinese Academy of Sciences, Beijing, China.
[4]School of Artificial Intelligence, University of Chinese Academy of Sciences, Beijing, China.
[5]Department of Biomedical Sciences, Jockey Club College of Veterinary Medicine and Life Sciences, City University of Hong Kong, Hong Kong SAR, China.
[6]Centre for Applied One Health Research and Policy Advice, Jockey Club College of Veterinary Medicine and Life Sciences, City University of Hong Kong, Hong Kong SAR, China.
[*]qingpeng.zhang@cityu.edu.hk; dajun.zeng@ia.ac.cn

Supplementary Note 1. Details of the probabilistic bias analysis in model initialization

We adopt the probabilistic bias analysis [1, 2] to correct the numbers of infections, recoveries, deaths, and active cases in all countries.

Denote N_i, $N_{i,te}(t)$, and $N_{i,unt}(t)$ as the population size, the cumulative number of tested individuals, and the number of untested individuals by time t for country i, respectively. $N_i = N_{i,te}(t) + N_{i,unt}(t)$. For simplicity, we drop (t) in the following description. Denote $N^+_{i,te}$ and $N^+_{i,unt}$ as the number of confirmed cases among tested individuals and the estimated number of confirmed cases among untested individuals in country i. $N^+_i = N^+_{i,te} + N^+_{i,unt}$ represents the estimated number of confirmed cases if country i tests its entire population. Due to imperfect tests, the actual number of infected cases in country i, denoted by N^*_i, is

$$N^*_i = \frac{N^+_i - (1-p)N_i}{e+p-1},$$

where e and p represent the test sensitivity (the ability to identify patients with a disease correctly) and test specificity (the ability to identify patients without a disease correctly). Denote $N^+_{i,unt,MS}$ ($N^+_{i,unt,MN}$) as the estimated number of confirmed cases among untested individuals who have moderate or severe (mild or no) symptoms if tested. Thus,

$$\begin{aligned}
N^+_{i,unt} &= N^+_{i,unt,MS} + N^+_{i,unt,MN},\\
N^+_{i,unt,MS} &= N_{i,unt}\mathscr{P}_i(MS|unt)\mathscr{P}_i(test+|unt,MS),\\
N^+_{i,unt,MN} &= N_{i,unt}[1-\mathscr{P}(MS|unt)]\mathscr{P}_i(test+|unt,MN).
\end{aligned}$$

Here, $\mathscr{P}_i(MS|unt)$ represents the fraction of individuals with moderate or severe symptoms among untested individuals in country i; $\mathscr{P}_i(test+|unt,MS)$ ($\mathscr{P}_i(test+|unt,MN)$) presents the test positivity rate of untested individuals with moderate or severe (mild or no) symptoms for country i. Denote

$$\begin{aligned}
q_{MS} &= \frac{\mathscr{P}_i(test+|unt,MS)}{\mathscr{P}_i(test+|t)},\\
q_{MN} &= \frac{\mathscr{P}_i(test+|unt,MN)}{\mathscr{P}_i(test+|t)}.
\end{aligned}$$

Here $\mathscr{P}_i(test+|t)$ is the positive rate among tested individuals in country i, which is estimated by the $N^+_{i,te}/N_{i,te}$. Therefore,

$$\begin{aligned}
N^*_i &= \frac{N^+_{i,te} + N^+_{i,unt} - (1-p)N_i}{e+p-1}\\
&= \frac{N^+_{i,te} + N^+_{i,unt,MS} + N^+_{i,unt,MN} - (1-p)N_i}{e+p-1}\\
&= \frac{N^+_{i,te} + N_{i,unt}\frac{N^+_{i,te}}{N_{i,te}}[(q_{MS}-q_{MN})\mathscr{P}_i(MS|unt) + q_{MN}] - (1-p)N_i}{e+p-1}.
\end{aligned} \tag{S1}$$

Denote the actual number of infected cases among tested individuals as $N^*_{i,te}$ for country i, then,

$$N^*_{i,te} = \frac{N^+_{i,te} - (1-p)N_{i,te}}{e+p-1}.$$

We assume that (1) deaths are accurately reported among individuals who are tested positive; (2) COVID-19 accounts for all reported deaths among individuals who tested positive; and (3) the case fatality rate among tested individuals and untested individuals are the same. Thus, the actual case fatality rate (CFR) for country i is

$$CFR_i^* = \frac{D_{i,te}^+}{eN_{i,te}^*},$$

where $D_{i,te}^+$ is the reported deaths in country i. Then, the actual number of deaths in country i is

$$D_i^* = CFR_i^* * N_i^*. \tag{S2}$$

Due to limited healthcare resources (e.g., manpower, hospital beds, etc.) for patient monitoring, the health status for some positive cases with mild symptoms is never counted in the official data. Assuming that (1) only $r_{i,de}$ fraction of recovered cases who tested positive are detected in country i and (2) the fraction of recovered individuals among false positive cases is ν_i in country i, then the actual number of recovered individuals who tested cases is

$$R_{i,te}^* = \frac{R_{i,te}^+}{r_{i,de}} - \nu_i(N_{i,te}^+ - eN_{i,te}^*),$$

where $R_{i,te}^+$ is the reported number of recovered individuals for country i. See the table below for better understanding.

	Total number	Total deaths	Total recoveries
Tested positive	$N_{i,te}^+$	$D_{i,te}^+$	$R_{i,te}^+/r_{i,de}$
True positive	$eN_{i,te}^*$	$D_{i,te}^+$	$\frac{R_{i,te}^+}{r_{i,de}} - \nu_i(N_{i,te}^+ - eN_{i,te}^*)$
False positive	$N_{i,te}^+ - eN_{i,te}^*$	0	$\nu_i(N_{i,te}^+ - eN_{i,te}^*)$

Note that,

$$N_{i,te}^+ \geq R_{i,te}^+/r_{i,de} + D_{i,te}^+, \tag{S3}$$

thus, $1 \geq r_{i,de} \geq R_{i,te}^+/(N_{i,te}^+ - D_{i,te}^+)$;

$$R_{i,te}^+/r_{i,de} \geq \nu_i(N_{i,te}^+ - eN_{i,te}^*) \geq R_{i,te}^+/r_{i,de} - (eN_{i,te}^* - D_{i,te}^+), \tag{S4}$$

thus, $[R_{i,te}^+/r_{i,de} - (eN_{i,te}^* - D_{i,te}^+)]/(N_{i,te}^+ - eN_{i,te}^*) \leq \nu_i \leq R_{i,te}^+/[r_{i,de}(N_{i,te}^+ - eN_{i,te}^*)]$. The actual number of recovered individuals in country i is

$$R_i^* = \frac{R_{i,te}^*}{eN_{i,te}^*} * N_i^*. \tag{S5}$$

Thus, the actual number of active cases (i.e., currently infected cases) in country i is

$$I_i^* = N_i^* - R_i^* - D_i^*. \tag{S6}$$

We summarize all denotations in probabilistic bias analysis in Supplementary Table 1. Following [1], we define e, p, q_{MS}, q_{MN}, $\mathscr{P}_i(MS|unt)$, $r_{i,de}$, and ν_i as truncated beta distributions. We specify the ranges and median values of these parameters as follows. Currently, the real time reverse transcription–polymerase chain reaction (RT-PCR) test is the most commonly used

method worldwide for diagnosing COVID-19 [3], so we take its test performance as the reference in the paper. We set e and p range from 65% to 100% (mean: 80%) [4] and 99.98% to 100% (mean: 99.99%) [5], respectively.

According to the case-finding guidelines in most countries [6, 7, 8, 9], individuals with symptoms of COVID-19 or acute respiratory infection or asymptomatic individuals who are close contacts of confirmed cases should be tested. Thus, the test positivity rate of tested individuals should be higher among tested individuals. Besides, the chance of getting COVID-19 is higher among individuals with symptoms than those without symptoms, we assume that $\mathscr{P}_i(test+|unt, MN) < \mathscr{P}_i(test+|unt, MS) < \mathscr{P}_i(test+|t)$. We set the ranges of q_{MS} and q_{MN} as 85% to 100% (mean: 90%) and 20% to 40% (mean: 25%), respectively, based on the studies in [1, 10, 11] .

We set the range of $\mathscr{P}_i(MS|unt)$ as 10% to 50% (mean: 30%), which is a reasonable estimate based on the following studies. A study collecting data from a large-scale surveillance study between May and October 2020 in India reported that, 37% of tested individuals are with symptoms potentially associated with COVID-19 [10]. In this study, a majority of tests are administrated to individuals in risk groups. Another study collecting data among people who completed their quarantine between August and October 2020 in Singapore reported that 20% of quarantined people have symptomatic illness [12]. Two studies focusing on health care workers (HCWs) across nine countries (India, South Africa, UK, Romania, etc.) reported that the fraction of HCWs had symptoms compatible with COVID-19 ranges from 0.81% to 56.8% based on data between March to mid-July 2020 [13, 14]. These figures help inform the prior distribution of $\mathscr{P}_i(MS|unt)$ in the general population although they may not be the actual values because of the differences in epidemic prevalence and risk exposure levels. There is no evidence to inform the the prior distributions of $r_{i,de}$, and ν_i. We roughly define the prior distribution of $r_{i,de}$ and ν_i as truncated beta distributions based on the constrains in Supplementary Eqs. (S3) and (S4). $N_{i,te}$ is estimated by the number of tests performed in country i (not exceeding the population size), which can be obtained from Our World in Data by the University of Oxford and the Global Change Data Lab [15]. $N^+_{i,te}$ is the official number of total confirmed cases for country i, which can be obtained from the Johns Hopkins Coronavirus Resource Center [16].

We run the estimation process for 10^5 iterations. For each iteration, we randomly sample the values of bias parameters e, p, q_{MS},q_{MN}, $\mathscr{P}_i(MS|unt)$, $r_{i,de}$, and ν_i from their prior distributions. Then, we apply these values to generate a single bias-adjusted estimate of N^*_i, D^*_i, R^*_i, and I^*_i based on Supplementary Eqs. (S1), (S2), (S5), and (S6). After 10^5 iterations, we can obtain the distributions of bias-adjusted estimates of N^*_i, D^*_i, R^*_i, and I^*_i for each country. We correct the numbers of total infections, recoveries, deaths, active cases as the median values of all estimates. Supplementary Figure 1 shows the estimated numbers vs. reported numbers of cumulative infections, cumulative recoveries, cumulative deaths, and active cases on June 15, 2021, for ten countries with the largest number of estimated cumulative infections.

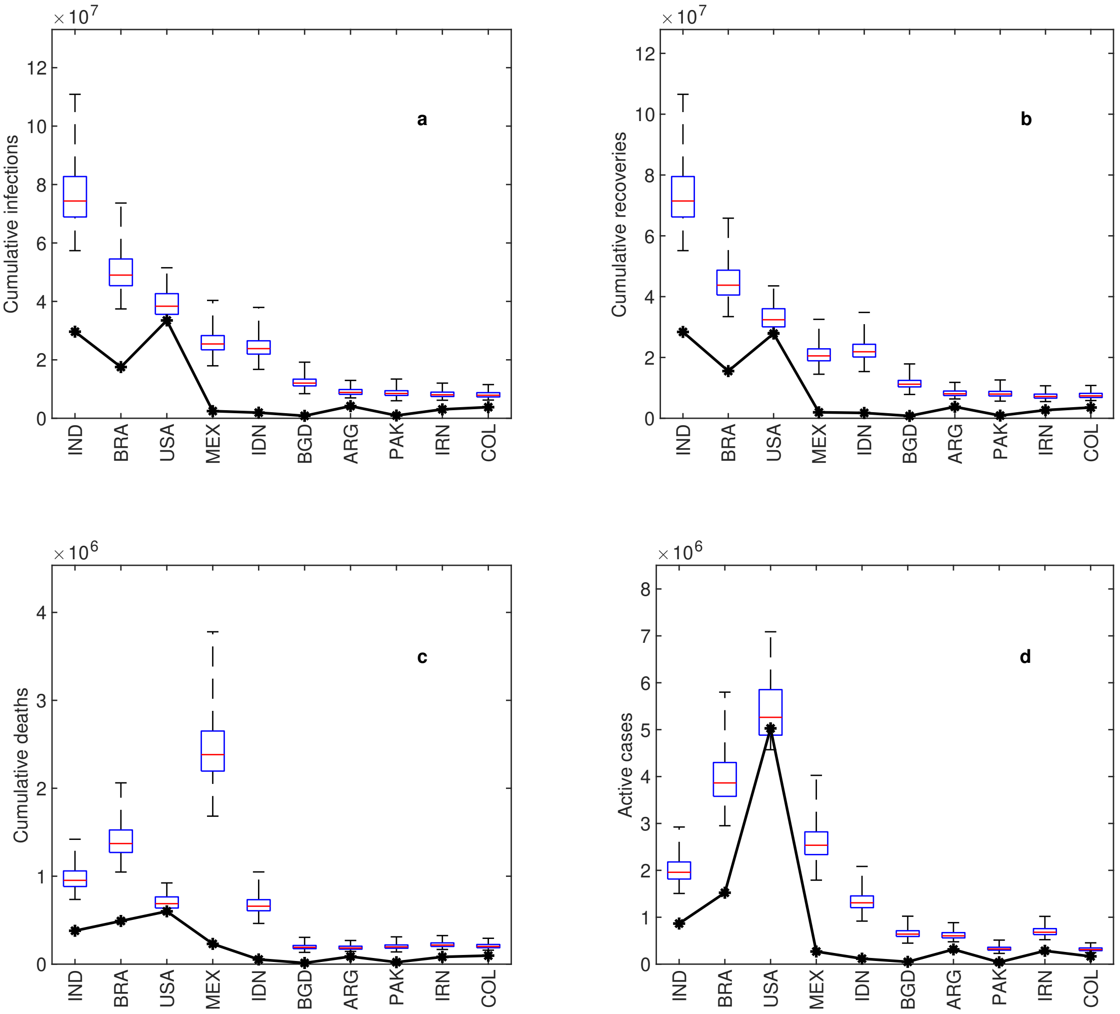

Supplementary Figure 1: Estimated numbers vs. reported numbers of cumulative infections, cumulative recoveries, cumulative deaths, and active cases on June 15, 2021, for ten countries with the largest numbers of estimated cumulative infections. **a**, Cumulative infections. **b** Cumulative recoveries. **c**, Cumulative deaths. **d**, Active cases. Box plots show the distribution of estimated numbers, which were obtained via 10^5 Monte Carlo samples from the distribution of estimated numbers. The central mark (the red line) of each box indicates the median. The bottom and top edges of each box indicate the 25th and 75th percentiles. The whiskers extend to the minimum and maximum values of estimated numbers. Black asterisks (*) indicate the reported numbers for each country. Only 3-letter ISO codes for countries are presented for a clear illustration. See country codes list in Supplementary Table 2.

Supplementary Table 1: Denotations in probabilistic bias analysis

Denotation	Definition
N_i	Population size of country i
$N_{i,te}$	The cumulative number of tested population in country i
$N_{i,unt}$	The number of untested population in country i
N_i^+	The estimated number of confirmed cases if country i tests its entire population
$N_{i,te}^+$	The number of confirmed cases among tested individuals in country i
$N_{i,unt}^+$	The estimated number of confirmed cases among untested individuals
e	Test sensitivity
p	Test specificity
$N_{i,unt,MS}^+$	The estimated number of confirmed cases among untested individuals who have moderate or severe symptoms if tested for country i
$N_{i,unt,MN}^+$	The estimated number of confirmed cases among untested individuals who have mild or no symptoms if tested for country i
$\mathscr{P}_i(MS\|unt)$	The fraction of individuals with moderate or severe symptoms among untested individuals in country i
$\mathscr{P}_i(test+\|unt, MS)$	The test positivity rate of untested individuals with moderate or severe symptoms for country i
$\mathscr{P}_i(test+\|unt, MN)$	The test positivity rate of untested individuals with mild or no symptoms for country i
$\mathscr{P}_i(test+\|t)$	The test positivity rate of tested individuals in country i
q_{MS}	$\frac{\mathscr{P}_i(test+\|unt,MS)}{\mathscr{P}_i(test+\|t)}$
q_{MN}	$\frac{\mathscr{P}_i(test+\|unt,MN)}{\mathscr{P}_i(test+\|t)}$
N_i^*	The actual number of infected cases in country i
$N_{i,te}^*$	The actual number of infected cases among tested individuals in country i
CFR_i^*	The actual case fatality rate for country i
$D_{i,te}^+$	The reported deaths in country i
D_i^*	The actual number of deaths in country i
$R_{i,te}^+$	The reported recoveries in country i
$r_{i,de}$	The fraction of detected recovered individuals among recovered individuals who are tested positive for country i
ν_i	The fraction of recovered individuals among false positive cases for country i
$R_{i,te}^*$	The actual number of recoveries among tested individuals in country i
R_i^*	The actual number of recovered individuals in country i
I_i^*	The actual number of active cases in country i

Supplementary Table 2: Country Alpha 3 ISO codes list

Country name	Alpha 3 ISO code
India	IND
Brazil	BRA
United States of America	USA
Mexico	MEX
Indonesia	IDN
Bangladesh	BGD
Argentina	ARG
Pakistan	PAK
Iran	IRN
Colombia	COL

Supplementary Note 2. Details of the adaptive policy adoption strategy

Following [17], we integrate the impact of NPIs through a reduction in the value of the basic reproduction number and consider two levels of NPI intensity: stringent and mild NPIs. Denote the effectiveness of stringent and mild NPIs as $c_{stringent}$ and c_{mild}, respectively. Then, the effectiveness of NPIs at time t for country i is represented by

$$c_i(t) = \begin{cases} c_{stringent}, & R_{e,i}(t) \geq 1, \\ c_{mild}, & R_{e,i}(t) < 1. \end{cases}$$

$R_{e,i}(t)$ is the local effective reproduction number for country i at time t. According to the assessment of different control measures based on the real-world data [18, 19], we set the effectiveness of stringent and mild NPIs as 80% and 40%, respectively. $R_{e,i}(t)$ can be computed as the dominant eigenvalue of the Next Generation Matrix (NGM) [20] associated with the dynamical system considered. The local disease transmission dynamics within country i (when it is isolated from the metapopulation network) is represented by the following equations:

$$\begin{aligned}
\partial_t S_i(t) &= -\phi_i(t) - \sum_m \sum_n [\mathcal{C}_i^S(t)\mathcal{T}\mathcal{U}]_{n,m} + \varepsilon V_i(t), \\
\partial_t V_i(t) &= \phi_i(t) - \sum_m \sum_n (1-\eta_n)[\mathcal{C}_i^V(t)\mathcal{T}\mathcal{U}]_{n,m} - \varepsilon V_i(t), \\
\partial_t E_{i,m}^S(t) &= \sum_n [\mathcal{C}_i^S(t)\mathcal{T}\mathcal{U}]_{n,m} - \sigma E_{i,m}^S(t), \\
\partial_t E_{i,m}^V(t) &= \sum_n (1-\eta_n)[\mathcal{C}_i^V(t)\mathcal{T}\mathcal{U}]_{n,m} - \sigma E_{i,m}^V(t), \\
\partial_t I_{i,m}^S(t) &= \sigma E_{i,m}^S(t) - \alpha I_{i,m}^S(t), \\
\partial_t I_{i,m}^V(t) &= \sigma E_{i,m}^V(t) - \alpha I_{i,m}^V(t), \\
\partial_t R_i(t) &= \sum_m (1-\mathcal{F}_{i,m})\alpha I_{i,m}^S(t) + \sum_m [1-(1-\epsilon_m)\mathcal{F}_{i,m}]\alpha I_{i,m}^V(t), \\
\partial_t D_i(t) &= \sum_m \mathcal{F}_{i,m}\alpha I_{i,m}^S(t) + \sum_m (1-\epsilon_m)\mathcal{F}_{i,m}\alpha I_{i,m}^V(t).
\end{aligned}$$

This system has $4M$ infected states ($E_{i,m}^S$, $E_{i,m}^V$, $I_{i,m}^S$, and $I_{i,m}^V$ for strain m). The infected subsystem is

$$\begin{aligned}
\partial_t E_{i,m}^S(t) &= \sum_n [\mathcal{C}_i^S(t)\mathcal{T}\mathcal{U}]_{n,m} - \sigma E_{i,m}^S(t), \\
\partial_t E_{i,m}^V(t) &= \sum_n (1-\eta_n)[\mathcal{C}_i^V(t)\mathcal{T}\mathcal{U}]_{n,m} - \sigma E_{i,m}^V(t), \\
\partial_t I_{i,m}^S(t) &= \sigma E_{i,m}^S(t) - \alpha I_{i,m}^S(t), \\
\partial_t I_{i,m}^V(t) &= \sigma E_{i,m}^V(t) - \alpha I_{i,m}^V(t).
\end{aligned}$$

The infected subsystem is composed by the state variable $x = [E_{i,1}^S, \ldots, E_{i,M}^S, E_{i,1}^V, \ldots, E_{i,M}^V, I_{i,1}^S, \ldots, I_{i,M}^S, I_{i,1}^V, \ldots, I_{i,M}^V]$. Then, we can construct the transmission term $\mathbf{V_{F,i}}$

and the transition term $\mathbf{V_{F,i}}$ as follows.

$$\mathbf{V_{F,i}} = \begin{bmatrix} \sum_n [\mathcal{C}_i^S \mathcal{T}\mathcal{U}]_{n,1} \\ \vdots \\ \sum_n [\mathcal{C}_i^S \mathcal{T}\mathcal{U}]_{n,M} \\ \sum_n (1-\eta_n)[\mathcal{C}_i^V \mathcal{T}\mathcal{U}]_{n,1} \\ \vdots \\ \sum_n (1-\eta_n)[\mathcal{C}_i^V \mathcal{T}\mathcal{U}]_{n,M} \\ 0 \\ \vdots \\ 0 \\ 0 \\ \vdots \\ 0 \end{bmatrix},$$

and

$$\mathbf{V_{V,i}} = \begin{bmatrix} \sigma E_{i,1}^S \\ \vdots \\ \sigma E_{i,M}^S \\ \sigma E_{i,1}^V \\ \vdots \\ \sigma E_{i,M}^V \\ \alpha I_{i,1}^S - \sigma E_{i,1}^S \\ \vdots \\ \alpha I_{i,M}^S - \sigma E_{i,M}^S \\ \alpha I_{i,1}^V - \sigma E_{i,1}^V \\ \vdots \\ \alpha I_{i,M}^V - \sigma E_{i,M}^V \end{bmatrix}.$$

Hence, the transmission matrix $\mathbf{M_{F,i}}$ and the transition matrix $\mathbf{M_{V,i}}$ become

$$\mathbf{M_{F,i}} = \begin{bmatrix} \mathbf{0} & \mathbf{0} & (1-c_i)S_i/N_i(\mathcal{T}\mathcal{U})^T & (1-c_i)S_i/N_i(\mathcal{T}\mathcal{U})^T \\ \mathbf{0} & \mathbf{0} & (1-c_i)V_i/N_i(M_\eta \mathcal{T}\mathcal{U})^T & (1-c_i)V_i/N_i(M_\eta \mathcal{T}\mathcal{U})^T \\ \mathbf{0} & \mathbf{0} & \mathbf{0} & \mathbf{0} \\ \mathbf{0} & \mathbf{0} & \mathbf{0} & \mathbf{0} \end{bmatrix},$$

and

$$\mathbf{M_{V,i}} = \begin{bmatrix} \sigma \mathbb{1}_{2M*2M} & \mathbf{0} \\ -\sigma \mathbb{1}_{2M*2M} & \alpha \mathbb{1}_{2M*2M} \end{bmatrix},$$

where $M_\eta = diag(1-\eta_1, \ldots, 1-\eta_M)$. Therefore, the next generation matrix for country i is given by

$$
\begin{aligned}
NGF_i &= \mathbf{M_{F,i}}\mathbf{M_{V,i}}^{-1} \\
&= \begin{bmatrix} \mathbf{0} & \mathbf{0} & (1-c_i)S_i/N_i(\mathcal{T}\mathcal{U})^T & (1-c_i)S_i/N_i(\mathcal{T}\mathcal{U})^T \\ \mathbf{0} & \mathbf{0} & (1-c_i)V_i/N_i(M_\eta\mathcal{T}\mathcal{U})^T & (1-c_i)V_i/N_i(M_\eta\mathcal{T}\mathcal{U})^T \\ \mathbf{0} & \mathbf{0} & \mathbf{0} & \mathbf{0} \\ \mathbf{0} & \mathbf{0} & \mathbf{0} & \mathbf{0} \end{bmatrix} \begin{bmatrix} \frac{1}{\sigma}\mathbb{1} & \mathbf{0} \\ \frac{1}{\alpha}\mathbb{1} & \frac{1}{\alpha}\mathbb{1} \end{bmatrix}.
\end{aligned}
$$

The local effective reproduction number of country i is the dominant eigenvalue of NGF_i. In the main text, we assume that the threshold of the local effective reproduction number leading to stringent NPIs is 1. Sensitivity analysis results (Supplementary Figures 28-32) show that, relaxation of NPIs before the pandemic is well-contained substantially extends the duration of the pandemic and leads to more deaths globally. With a higher threshold leading to stringent NPIs, HICs need to donate more vaccines to protect themselves.

Supplementary Note 3. Estimation of country-level severity matrices

To account for the heterogeneity in the healthcare burden of COVID-19 and the age structure in different countries, we set a country-specific severity matrix in our model. The severity of strain m for country i ($\mathcal{F}_{i,m}$) corresponds to the infection fatality rate of strain m for country i. Generally, the infection fatality rate is crudely estimated by the case fatality rate, i.e., the proportion of deaths among identified confirmed cases. Denote the infection fatality rate for country i as IFR_i, then,

$$\begin{aligned}\mathcal{F}_{i,1} = IFR_i &= \sum_a \mathscr{P}_i(deceased|infected, a)\frac{\mathscr{P}_i(infected|a)\mathscr{P}_i(a)}{\mathscr{P}_i(infected)} \\ &\approx \sum_a CFR_{i,a}\frac{\mathscr{P}_i(infected|a)\mathscr{P}_i(a)}{\mathscr{P}_i(infected)}\end{aligned}$$

where a denotes a specific age group, $\mathscr{P}_i(deceased|infected, a)$ represents the probability of dying from the disease for infected individuals at age group a, $CFR_{i,a}$ denotes the age-specific case fatality rate for age group a in country i, $\mathscr{P}_i(infected|a)$ represents the probability of getting infected for individuals in age group a in country i, $\mathscr{P}_i(infected)$ denotes the probability of getting infected in country i, and $\mathscr{P}_i(a)$ represents the proportion of individuals in age group a, as a share of the whole population in country i. Due to limited data for age-specific case morbidity and fatality rates for a specific age group in each country, we set the same values of $CFR_{i,a}$ and $\mathscr{P}_i(infected|a)/\mathscr{P}_i(infected)$ among HICs and LMICs, i.e.,

$$CFR_{i,a} = \begin{cases} CFR_{H,a}, & i \in H, \\ CFR_{L,a}, & i \in L, \end{cases}$$

and

$$\frac{\mathscr{P}_i(infected|a)}{\mathscr{P}_i(infected)} = \begin{cases} \frac{\mathscr{P}_H(infected|a)}{\mathscr{P}_H(infected)}, & i \in H, \\ \frac{\mathscr{P}_L(infected|a)}{\mathscr{P}_L(infected)}, & i \in L. \end{cases}$$

The values of $CFR_{H,a}$, $CFR_{L,a}$, $\frac{\mathscr{P}_H(infected|a)}{\mathscr{P}_H(infected)}$, and $\frac{\mathscr{P}_L(infected|a)}{\mathscr{P}_L(infected)}$ are computed based on data from India and the United States [21]. Specific values are shown in Supplementary Tables 3 and 4. $\mathscr{P}_i(a)$ is adopted from the latest version of World Population Prospects (`https://population.un.org/wpp/`). Overall, the average case fatality rates among HICs and LMICs are 0.03 and 0.01, respectively.

Supplementary Table 3: Values of $CFR_{H,a}$ and $\mathscr{P}_H(infected|a)/\mathscr{P}_H(infected)$ for each age group. All values are computed based on the data collected in the United States in [21].

Age group	$CFR_{H,a}$	$\mathscr{P}_H(infected\|a)/\mathscr{P}_H(infected)$
0-4 years	0.000469	0.272094
5-17 years	0.000178	0.374405
18-29 years	0.000743	1.345548
30-39 years	0.002493	1.277579
40-49 years	0.006584	1.299682
50-64 years	0.023755	1.115650
65-74 years	0.087082	0.806557
75-84 years	0.187557	0.955430
$\geq$ 85 years	0.295713	1.912334

Supplementary Table 4: Values of $CFR_{L,a}$ and $\mathscr{P}_L(infected|a)/\mathscr{P}_L(infected)$ for each age group. All values are computed based on the data collected in India in [21].

Age group	$CFR_{H,a}$	$\mathscr{P}_H(infected\|a)/\mathscr{P}_H(infected)$
0-4 years	0.000743	0.186034
5-17 years	0.000446	0.372069
18-29 years	0.000855	1.150155
30-39 years	0.002845	1.330793
40-49 years	0.008077	1.214549
50-64 years	0.023537	1.270905
65-74 years	0.058491	1.140730
75-84 years	0.096675	1.0287501
$\geq$ 85 years	0.130241	0.758821

Supplementary Note 4. Details of the multi-strain model

An infected individual with strain m infects each of its contacts independently with probability $\mathcal{T}_m$, then, recovers or deceases with probability $[1-(1-\epsilon_m)\mathcal{F}_{i,m}]\alpha$ and $(1-\epsilon_m)\mathcal{F}_{i,m}\alpha$, respectively. The virus mutates when adapting to a new host before subsequent infections. The virus in strain m can either remains as strain m with probability $1 - \mu_m$ or mutates to strain $m + 1$ (one-direction stepwise mutation) with probability μ_m. In other words, a susceptible individual receiving infection from an infected individual with strain m can either become an infected individual with strain m (with probability $1 - \mu_m$) or one with strain $m + 1$ (with probability μ_m).

Supplementary Note 5. Details of global vaccine allocation strategies

Available vaccines will be allocated to each country based on the prioritization criteria and the vaccine demand for each country. Denote $\Omega_i(t)$ as the number of vaccines allocated to country i at time t. $\Omega_i(t)$ depends on the global supply of vaccines, the global vaccine allocation strategy, and the demand of vaccines for country i at time t. Denote $vs_i(t)$ as the vaccine stock held by country i at time t, then the demand of vaccines for country i at time t is $dem_i(t) = max\{2S_i(t) - vs_i(t), 0\}$, which means each country orders vaccines that can vaccinate the entire susceptible population. Specifically, each global vaccine allocation strategy determines the value of $\Omega_i(t)$ in two steps. First, available vaccines will be allocated to each country based on the prioritization criteria. Second, the remaining vaccines will be allocated to each country based on the demand unmet in the first step. Four prioritization criteria are considered: the population size, prevalence, incidence, and the mortality rate. The number of vaccines allocated to country i at time t for each step is $\Omega_i^1(t)$ and $\Omega_i^2(t)$, $\Omega_i(t) = \Omega_i^1(t) + \Omega_i^2(t)$. We describe the details of each strategy as follows.

- **Equitable population size-based allocation.** In the first step, available daily vaccines will be allocated to all countries proportional to the population size, i.e.,

$$\Omega_i^1(t) = \min \{dem_i(t), [\varphi(t+1) - \varphi(t)]\frac{N_i}{\sum_j N_j}\}.$$

- **Equitable prevalence-based allocation.** In the first step, available daily vaccines will be allocated to all countries according to the prevalence, which is defined as the number of active cases (currently infectious cases) per capita, i.e.,

$$\Omega_i^1(t) = \min \{dem_i(t), [\varphi(t+1) - \varphi(t)]\frac{I_i(t)/N_i}{\sum_j I_j(t)/N_j}\},$$

- **Equitable incidence-based allocation.** In the first step, available daily vaccines will be allocated to all countries according to the incidence, which is defined as the number of new cases during two weeks as a share of the total population, i.e.,

$$\Omega_i^1(t) = \min \{dem_i(t), [\varphi(t+1) - \varphi(t)]\frac{I_i^{cum}(t) - I_i^{cum}(t-14)}{\sum_j I_j^{cum}(t) - I_j^{cum}(t-14)}\},$$

  where $I_i^{cum}(t)$ denotes the cumulative number of infectious cases as a share of the total population for country i at time t.

- **Equitable mortality rate-based allocation.** In the first step, available daily vaccines will be allocated to all countries according to the mortality rate, which is defined as the number of new deaths during two weeks as a share of the total population, i.e.,

$$\Omega_i^1(t) = \min \{dem_i(t), [\varphi(t+1) - \varphi(t)]\frac{D_i(t)/N_i - D_i(t-14)/N_i}{\sum_j D_j(t)/N_j - D_j(t-14)/N_j}\},$$

  where $D_i(t)$ denotes the cumulative number of deaths for country i at time t.

For all equitable strategies, in the second step, remaining vaccines will be allocated to each country proportional to the demand unmet in the first step, i.e.,

$$\Omega_i^2(t) = \min\ \{dem_i(t) - \Omega_i^1(t), [\varphi(t+1) - \varphi(t) - \sum_j \Omega_j^1(t)] \frac{dem_i(t) - \Omega_i^1(t)}{\sum_j dem_j(t) - \Omega_j^1(t)}\}.$$

For all inequitable strategies, a minimum fraction χ of vaccines available at time t are purchased to high-income countries (HICs), remaining vaccines are allocated to low- and middle-income countries (LMICs). Denote H and L as the sets of HICs and LMICs, respectively. Among each of the income groups, vaccines are allocated equitably according to one of the prioritization criteria. HICs are assumed to be allocated with vaccines no less than in the equitable strategies. Denote the actual fraction of global vaccine supply allocated to HICs at time t as $X(t)$ and $X(t) \geq \chi$. We specify the values of $X(t)$, $\Omega_i^1(t)$, and $\Omega_i^2(t)$ in each inequitable strategy as follows.

- **Inequitable population size-based allocation.**

$$X(t) = \max\ \{\chi, \frac{\sum_{j\in H} N_j}{\sum_j N_j}\}.$$

Thus,

$$\Omega_i^1(t) = \begin{cases} \min \left\{dem_i(t), \dfrac{N_i X(t)[\varphi(t+1) - \varphi(t)]}{\sum_{j\in H} N_j(t)}\right\} & i \in H, \\ \min \left\{dem_i(t), \dfrac{N_i[1 - X(t)][\varphi(t+1) - \varphi(t)]}{\sum_{j\in L} N_j(t)}\right\} & i \in L. \end{cases}$$

- **Inequitable prevalence-based allocation.**

$$X(t) = \max\ \{\chi, \frac{\sum_{j\in H} I_j(t)/N_j}{\sum_j I_j(t)/N_j}\}.$$

Thus,

$$\Omega_i^1(t) = \begin{cases} \min \left\{dem_i(t), \dfrac{I_i(t)/N_i]X(t)[\varphi(t+1) - \varphi(t)]}{\sum_{j\in H} I_j(t)/N_j}\right\} & i \in H, \\ \min \left\{dem_i(t), \dfrac{I_i(t)/N_i[1 - X(t)][\varphi(t+1) - \varphi(t)]}{\sum_{j\in L} I_j(t)/N_j}\right\} & i \in L. \end{cases}$$

- **Inequitable incidence-based allocation.**

$$X(t) = \max\ \{\chi, \frac{\sum_{j\in H} I_j^{cum}(t) - I_j^{cum}(t-14)}{\sum_j I_j^{cum}(t) - I_j^{cum}(t-14)}\}.$$

Thus,

$$\Omega_i^1(t) = \begin{cases} \min \left\{dem_i(t), \dfrac{[I_i^{cum}(t) - I_i^{cum}(t-14)]X(t)[\varphi(t+1) - \varphi(t)]}{\sum_{j\in H}[I_j^{cum}(t) - I_j^{cum}(t-14)]}\right\} & i \in H, \\ \min \left\{dem_i(t), \dfrac{[I_i^{cum}(t) - I_i^{cum}(t-14)][1 - X(t)][\varphi(t+1) - \varphi(t)]}{\sum_{j\in L}[I_j^{cum}(t) - I_j^{cum}(t-14)]}\right\} & i \in L. \end{cases}$$

- **Inequitable mortality rate-based allocation.**

$$X(t) = \max\ \{\chi, \frac{\sum_{j\in H} D_j(t)/N_j - D_j(t-14)/N_j}{\sum_j D_j(t)/N_j - D_j(t-14)/N_j}\}.$$

Thus,

$$\Omega_i^1(t) = \begin{cases} \min\ \Big\{dem_i(t), \frac{[\frac{D_i(t)}{N_i} - \frac{D_i(t-14)}{N_i}]X(t)[\varphi(t+1)-\varphi(t)]}{\sum_{j\in H}[D_j(t)/N_j - D_j(t-14)/N_j]}\Big\} & i \in H, \\ \min\ \Big\{dem_i(t), \frac{[\frac{D_i(t)}{N_i} - \frac{D_i(t-14)}{N_i}][1-X(t)][\varphi(t+1)-\varphi(t)]}{\sum_{j\in L}[D_j(t)/N_j - D_j(t-14)/N_j]}\Big\} & i \in L. \end{cases}$$

For all inequitable strategies, in the second step,

$$\Omega_i^2(t) = \begin{cases} \min\ \Big\{dem_i(t) - \Omega_i^1(t), & \\ \big\{X(t)[\varphi(t+1)-\varphi(t)] - \sum_{j\in H}\Omega_j^1(t)\big\}\frac{dem_i(t) - \Omega_i^1(t)}{\sum_{j\in H} dem_j(t) - \Omega_j^1(t)}\Big\} & i \in H, \\ \min\ \Big\{dem_i(t) - \Omega_i^1(t), & \\ \big\{[1-X(t)][\varphi(t+1)-\varphi(t)] - \sum_{j\in L}\Omega_j^1(t)\big\}\frac{dem_i(t) - \Omega_i^1(t)}{\sum_{j\in L} dem_j(t) - \Omega_j^1(t)}\Big\} & i \in L. \end{cases}$$

The number of individuals that can be fully vaccinated for country i at time t equals half the number of available vaccines for country i at time t, i.e., $\frac{\Omega_i(t)+vs_i(t)}{2}$. Denote the maximum daily vaccination rate for country i as $\overline{\phi}_i$. Then, the vaccination rate for country i at time t should not exceed either the available vaccine supply or the maximum daily vaccination rate, i.e., $\phi_i(t) = \min\ \{S_i(t), \frac{\Omega_i(t)+vs_i(t)}{2}, \overline{\phi}_i\}$. The vaccine stock held by country i at next time is $vs_i(t+1) = \Omega_i(t) + vs_i(t) - 2\phi_i(t)$. In all simulations, we set $\varphi(\tau) = \sum_i N_i$, and τ is 183 days. The upper bounds of daily vaccination rates for HICs and LMICs are the maximum daily vaccination rates achieved by HICs and LMICs by June 15, 2021.

$$\overline{\phi}_i = \begin{cases} N_i \max_{j\in H} \frac{\tilde{\phi}_j}{2N_j}, & i \in H. \\ N_i \max_{j\in L} \frac{\tilde{\phi}_j}{2N_j}, & i \in L, \end{cases}$$

where $\tilde{\phi}_i$ is the maximum daily vaccine doses administrated by country i from December 1, 2020, to June 15, 2021 ($t = 0$). After excluding the extremely high daily vaccination rates in Bhutan (with a maximum daily vaccination rate of 6% of the population), the upper bounds of the daily vaccination rate for HICs and LMICs are 1.2% and 1.9%, respectively.

Supplementary Note 6. Model parameter settings

The model works in a daily time step. For all simulations, we take $\sigma = 0.2$ corresponding to an incubation period of five days [22] and take $\alpha = 0.2$ corresponding to an infectious period of five days [19, 23]. Since no significant changes are found in the incubation period and infectious period [24], we assume them as identical for all strains. P_{ij} and γ are computed based on the real-world air traffic data in 2020 from OAG (`https://www.oag.com/`). $\gamma = 0.00015$ is the average (inflow/outflow) mobility rate per person per day in 2020 [25].

Currently, the duration of vaccinal immunity remains unclear. We set $1/\varepsilon = 365$ (days) based on publicly available clinical trial data [26, 27]. $\mathcal{T}_1$ is estimated by the initial basic reproduction number R_0 divided by the infectious period (5 days). Based on the phylogenetic analysis in the Global Initiative for Sharing All Influenza Data database [28], the global relative genome frequency of the Alpha, Beta, Gamma, and Delta is 43.8%, 1.2%, 8%, and 36.1%, respectively, from June 14, 2021 to June 20, 2021. The basic reproduction number is estimated to be 4 [29, 30], 4 [30], 4.4 [30], and 7 [29, 30], for the Alpha, Beta, Gamma, and Delta strain, respectively. No data is available for the remaining 10.9% of the genomes, we assume that they belong to the original strain ($R_0 = 2.79$). Therefore, we set $R_0 = 2.79 \times 10.9\% + 4 \times (43.8\% + 1.2\%) + 4.4 \times 8\% + 7 \times 36.1\% \approx 5$ at time 0. $\mathcal{F}_{i,1}$ is estimated by the case fatality rate for country i (see Supplementary Note 3). Currently, there are several vaccines with varying efficacy that have passed through Phase 3 clinical trials, we set η_1 and ϵ_1 as 0.828 and 0.967, respectively, according to the publicly available clinical trial data [31, 32].

Because of limited data to quantify the viral mutation parameters, M, θ, μ_1, and λ, we use the historical data for the first 1.5 years of the pandemic (from December 31, 2019, to June 15, 2021) to inform the values of these parameters. We assume the virus follows a similar mutation process in the future and perform sensitivity analysis on these parameters (Supplementary Figures 6-14, Supplementary Figures 20-24).

Most of the viral mutations have little impact on the virus' ability to transmit and cause severe infections. Variants meeting specific criteria (e.g., increase in transmissibility, increase in virulence, decrease in effectiveness of public health measures.) are designated as "Variants of Concern" by the World Health Organization [33]. Although there are thousands of genetic variants of SARS-CoV-2 [34], only four of them are designated as "Variants of Concern" as of June 15, 2021, i.e., the Alpha, Beta, Gamma, and Delta strain. Thus, the value of M for the first 1.5 years of the pandemic is 5. We assume M ranges from 3 to 10 in the next five years.

The most transmissible strain, Delta, demonstrates 2.5 times higher transmissibility compared to the original strain. Therefore, here we assume a linear strain space and local movement by a one-direction stepwise mutation [35], then the transmissibility of each new strain is 26% ($1.26^4 \approx 2.5$) higher than the immediate previous strain, i.e., $\theta = 0.26$ for the first 1.5 years of the pandemic. We assume θ ranges from 0.1 to 0.5 in the next five years.

There is evidence that mutations in the genome region encoding the spike protein (3822 nucleotides in length from site 21563 to 25384 [36]) may result in increased transmissibility [37] and viral load [38] of the virus. It is estimated that the mutation rate per virus replication cycle per site is 3×10^{-6} and the entire course of infection will take approximately five viral replication cycles [39]. Thus, the probability that the spike protein region changes per infection is $1 - ((1 - 3 \times 10^{-6})^5)^{3822} \approx 0.056$. Most of such mutations are neutral. Moreover, the virus cannot evolve indefinitely, primarily because each nucleotide can only mutate to three others (e.g., adenine (A) can only mutate to thymine (T) or guanine (G) or cytosine (C)), and we have limited number of nucleotides [40]. As the virus evolves in the strain space, the probability of major and new changes per infection decreases because fewer possible genome sequences remain. It means that the probability of emerging new and more dangerous strains per infection de-

creases over time [41]. Based on these facts, we assume that (a) for strain 1, only 0.01%-10% of such mutations can significantly affect the virus' ability to cause infections, i.e., μ_1 ranges from 5.6×10^{-6} to 5.6×10^{-3} in the next five years; (b) λ ranges from 10^2 to 10^4 in the next five years. We summarize all parameter symbols in the main text in Supplementary Table 5.

Supplementary Table 5: Model parameter symbols (in the main text)

Parameters	Symbol	Values
$\mathcal{T}$	The transmissibility matrix	
$\mathcal{F}_i$	The severity matrix for country i	
$\mathcal{U}$	The mutation matrix	
M	The number of possible strains emerged in the model	[3, 10]
$\mathcal{T}_1$	The transmissibility of strain 1	1
$\overline{\mathcal{F}_1}$	The average severity of strain 1	0.01737
μ_1	The mutation probability for strain 1	$[5.6 \times 10^{-6}, 5.6 \times 10^{-3}]$
λ	The decrease rate of the mutation probability	$[10^2, 10^4]$
d	The distance of antigenicity between the vaccine strain and a mutant strain when the cross-immunity is reduced to $1/e$	15
θ	The increase in transmissibility of each new strain	[0.1, 0.5]
η_{mn}	The efficacy against infection from strain n for vaccines designed for strain m	
ϵ_{mn}	The efficacy against death from strain n for vaccines designed for strain m	
η_1	The efficacy against infection for vaccines designed for strain 1	0.828
ϵ_1	The efficacy against death for vaccines designed for strain 1	0.967
N_i	The population size for country i	
S_i	The number of susceptible individuals in country i	
V_i	The number of vaccinated individuals in country i	
$E^S_{i,m}$	The number of individuals exposed to strain m (not yet infectious) without vaccinal immunity in country i	
$E^V_{i,m}$	The number of individuals exposed to strain m (not yet infectious) with vaccinal immunity in country i	
$I^S_{i,m}$	The number of infectious individuals caused by strain m without vaccinal immunity in country i	
$I^V_{i,m}$	The number of infectious individuals caused by strain m with vaccinal immunity in country i	
R_i	The number of recovered individuals in country i	
D_i	The number of deceased individuals in country i	

$\phi_i(t)$	The vaccination rate	
ε	The rate at which vaccinated individuals gradually lose vaccinal immunity and become fully susceptible again	1/365
$\mathcal{C}_i^S(t)$	Contacts between susceptible individuals and infectious individuals	
$\mathcal{C}_i^V(t)$	Contacts between vaccinated individuals and infectious individuals	
c_i	The effectiveness of non-pharmaceutical interventions	
σ	The reciprocal of the incubation period	0.2
α	The reciprocal of the infectious period	0.2
G_{ij}	The number of individuals travelling from country i to country j	
γ	The average mobility rate (inflow/outflow)	0.00015
A_i	The number of individuals allowed to travel from country i to other cities	
P_{ij}	The fraction of individuals travelling from country i to country j	
F_{ij}	The number of passengers traveling from country i to country j per day	
$\varphi(t)$	The cumulative global vaccine supply at time t	
τ	The time when the maximum daily vaccine production capacity is reached	183
v	The rate at which productivity grows	0.0076
χ	The minimum fraction of available vaccines that are purchased by HICs	{0.8, 0.9}

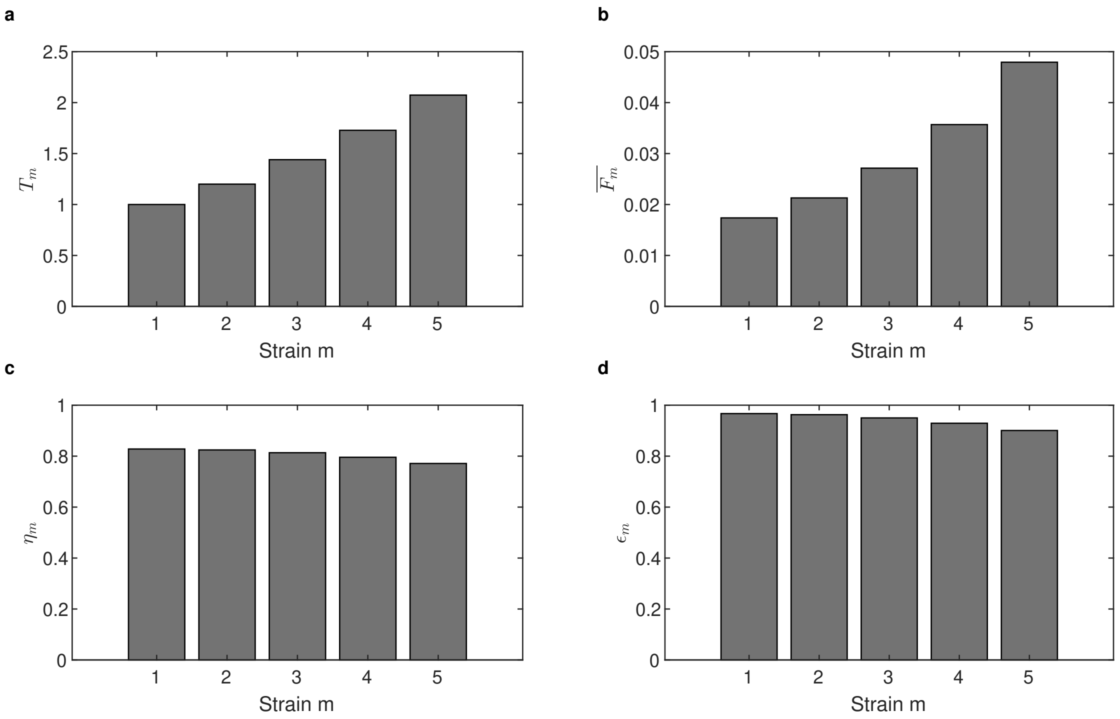

Supplementary Figure 2: The transmissibility and the average severity for each strain, and the vaccine efficacy against infection and death for each strain. **a** and **b**, The transmissibility ($\mathcal{T}_m$) and the average severity ($\overline{\mathcal{F}_m} = \sum_i \mathcal{F}_{i,m}/179$) of strain m, respectively. **c** and **d**, The vaccine efficacy against infection (η_m) and death (ϵ_m) from strain m. Parameter values $M = 5$ and $\theta = 0.2$.

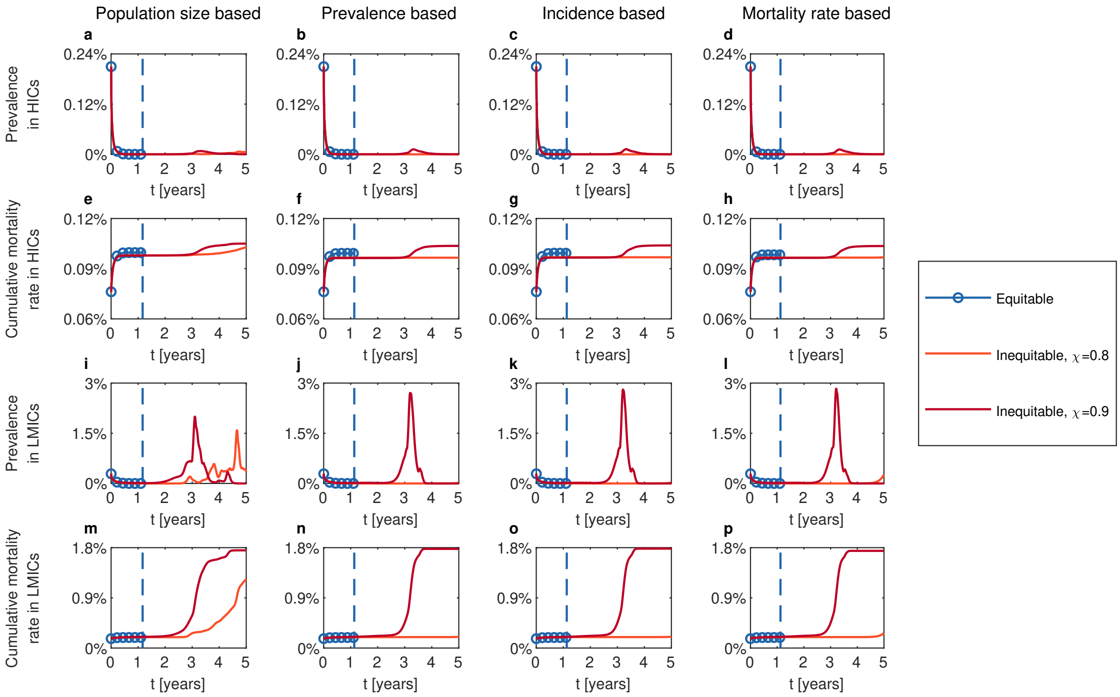

Supplementary Figure 3: Impact of equitable and inequitable vaccine allocation strategies on epidemic dynamics. **a-h**, Time series of the prevalence (**a-d**) and the cumulative mortality rate (**e-h**) in HICs under different global vaccine allocation strategies. **i-p**, Time series of the prevalence (**i-l**) and the cumulative mortality rate (**m-p**) in LMICs under different global vaccine allocation strategies. Four prioritization criteria for allocation are adopted: the population size (the left panel), prevalence (second left panel), incidence (second right panel), and mortality rate (the right panel). Dash lines indicate the time when the pandemic ends (time exceeding five years is not presented; dashed lines referring to the priority criterion are represented by the same colour). Parameter values $M = 5$, $\mu_1 = 5.6 \times 10^{-3}$, $\theta = 0.2$, and $\lambda = 5 \times 10^2$.

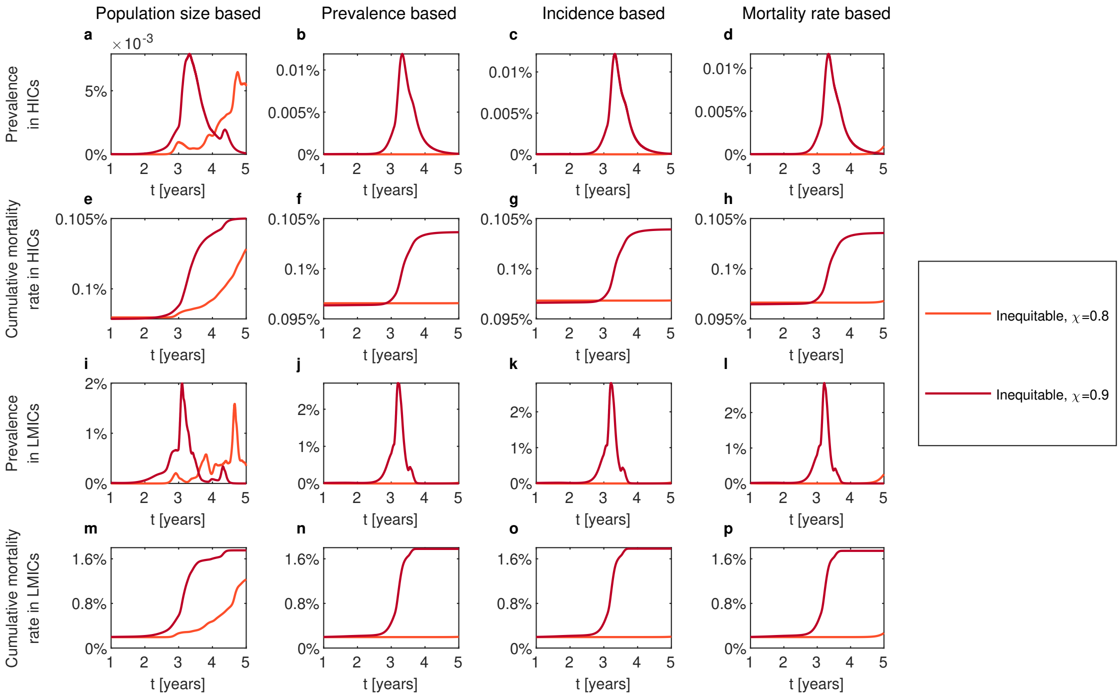

Supplementary Figure 4: Impact of inequitable vaccine allocation strategies on epidemic dynamics. **a-h**, Time series of the prevalence (**a-d**) and the cumulative mortality rate (**e-h**) in HICs under different global vaccine allocation strategies. **i-p**, Time series of the prevalence (**i-l**) and the cumulative mortality rate (**m-p**) in LMICs under different global vaccine allocation strategies. Four prioritization criteria for allocation are adopted: the population size (the left panel), prevalence (second left panel), incidence (second right panel), and mortality rate (the right panel). Results from the second year are presented. Parameter values $M = 5$, $\mu_1 = 5.6 \times 10^{-3}$, $\theta = 0.2$, and $\lambda = 5 \times 10^2$.

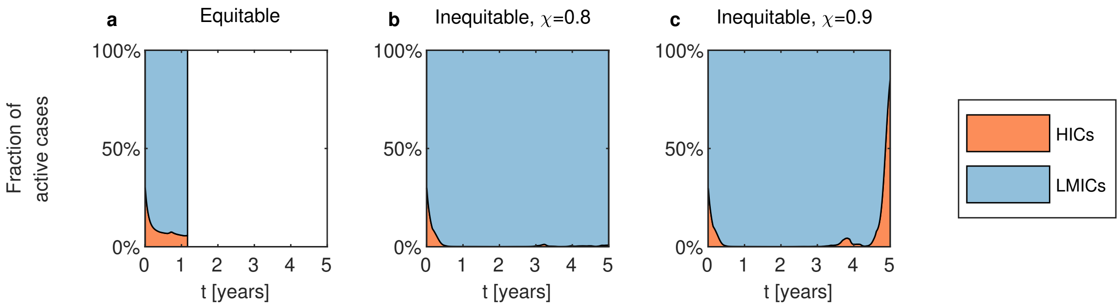

Supplementary Figure 5: Area plots of the fraction of active cases in HICs and LMICs. Figures in the left panel (a), the middle panel (b), and the right panel (c) are based on the equitable, inequitable and $\chi = 0.8$, and inequitable and $\chi = 0.9$ vaccine allocation strategies, respectively. All results are based on the prioritization criterion of the population size. Parameter values $M = 5$, $\mu_1 = 5.6 \times 10^{-3}$, $\theta = 0.2$, and $\lambda = 5 \times 10^2$.

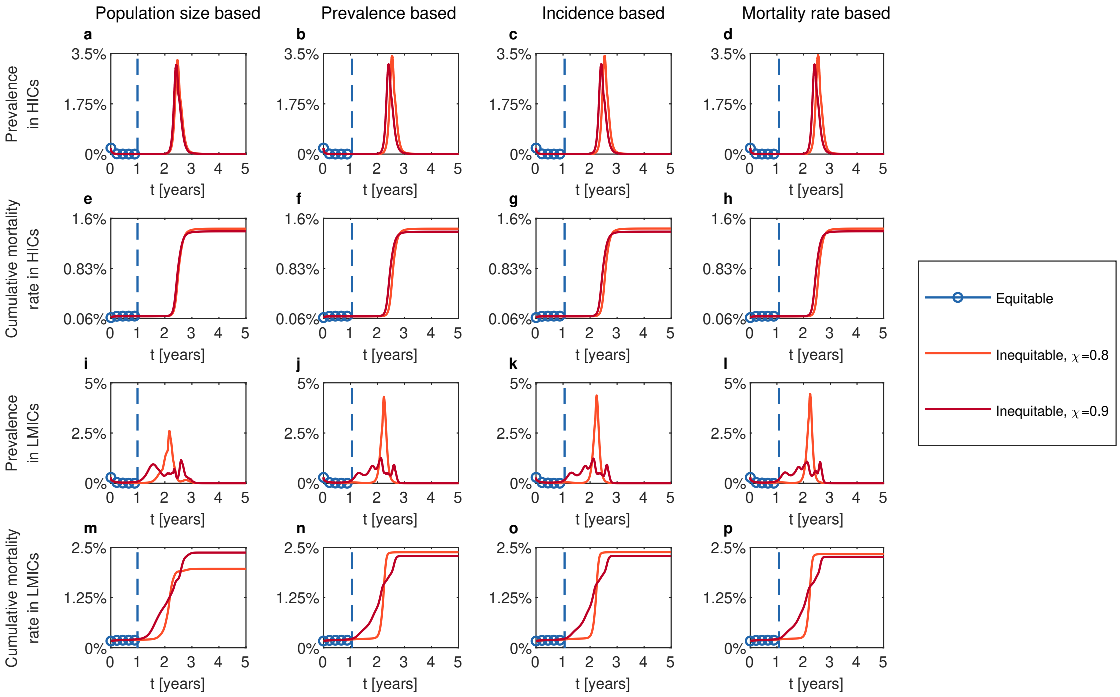

Supplementary Figure 6: Impact of equitable and inequitable vaccine allocation strategies on epidemic dynamics. **a-h**, Time series of the prevalence (**a-d**) and the cumulative mortality rate (**e-h**) in HICs under different global vaccine allocation strategies. **i-p**, Time series of the prevalence (**i-l**) and the cumulative mortality rate (**m-p**) in LMICs under different global vaccine allocation strategies. Four prioritization criteria for allocation are adopted: the population size (the left panel), prevalence (second left panel), incidence (second right panel), and mortality rate (the right panel). Dash lines indicate the time when the pandemic ends (time exceeding five years is not presented; dashed lines referring to the priority criterion are represented by the same colour. Parameter values $M = 6$, $\mu_1 = 5.6 \times 10^{-3}$, $\theta = 0.26$, and $\lambda = 10^2$.

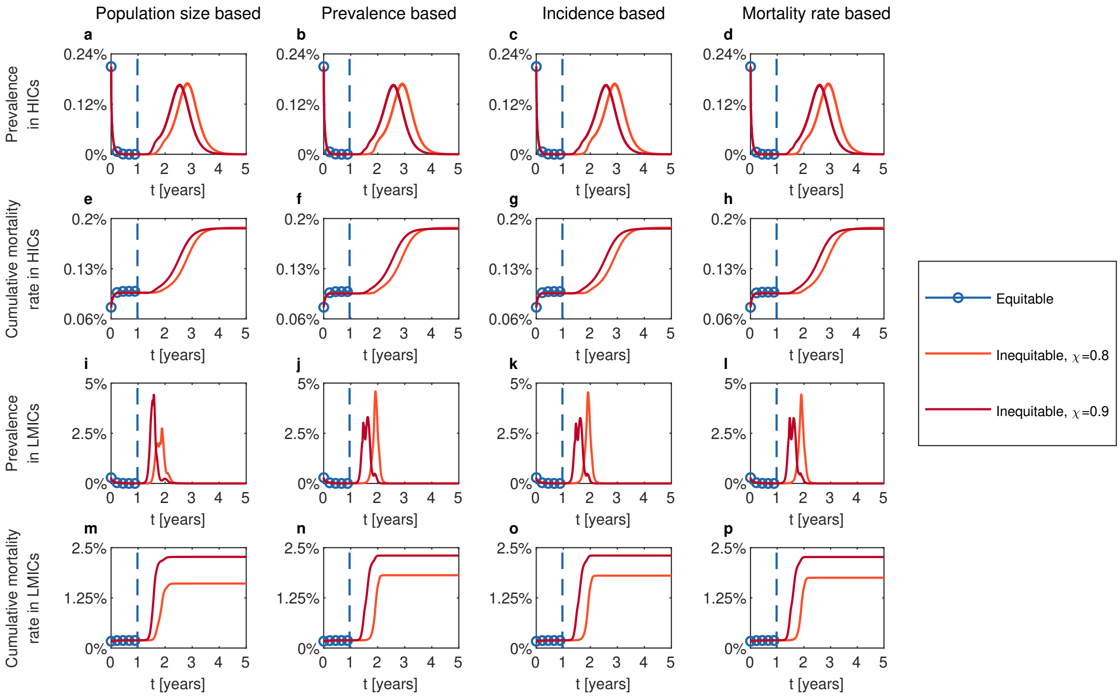

Supplementary Figure 7: Impact of equitable and inequitable vaccine allocation strategies on epidemic dynamics. **a-h**, Time series of the prevalence (**a-d**) and the cumulative mortality rate (**e-h**) in HICs under different global vaccine allocation strategies. **i-p**, Time series of the prevalence (**i-l**) and the cumulative mortality rate (**m-p**) in LMICs under different global vaccine allocation strategies. Four prioritization criteria for allocation are adopted: the population size (the left panel), prevalence (second left panel), incidence (second right panel), and mortality rate (the right panel). Dash lines indicate the time when the pandemic ends (time exceeding five years is not presented; dashed lines referring to the priority criterion are represented by the same colour. Parameter values $M = 4$, $\mu_1 = 5.6 \times 10^{-6}$, $\theta = 0.4$, and $\lambda = 10^2$.

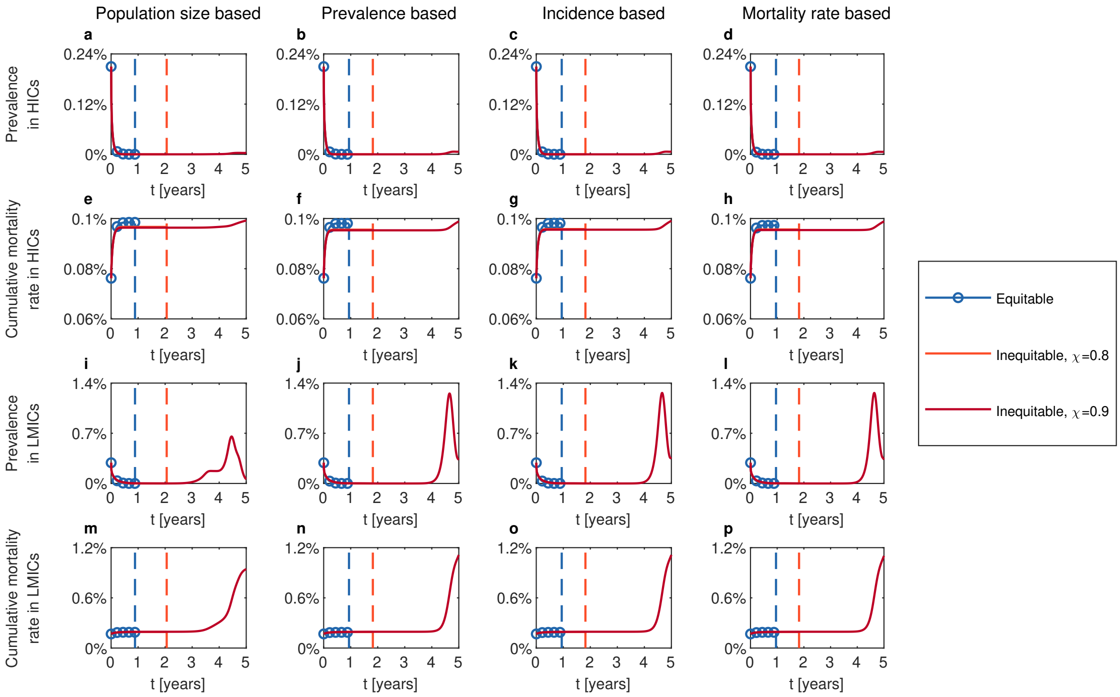

Supplementary Figure 8: Impact of equitable and inequitable vaccine allocation strategies on epidemic dynamics. **a-h**, Time series of the prevalence (**a-d**) and the cumulative mortality rate (**e-h**) in HICs under different global vaccine allocation strategies. **i-p**, Time series of the prevalence (**i-l**) and the cumulative mortality rate (**m-p**) in LMICs under different global vaccine allocation strategies. Four prioritization criteria for allocation are adopted: the population size (the left panel), prevalence (second left panel), incidence (second right panel), and mortality rate (the right panel). Dash lines indicate the time when the pandemic ends (time exceeding five years is not presented; dashed lines referring to the priority criterion are represented by the same colour. Parameter values $M = 7$, $\mu_1 = 5.6 \times 10^{-4}$, $\theta = 0.2$, and $\lambda = 10^4$.

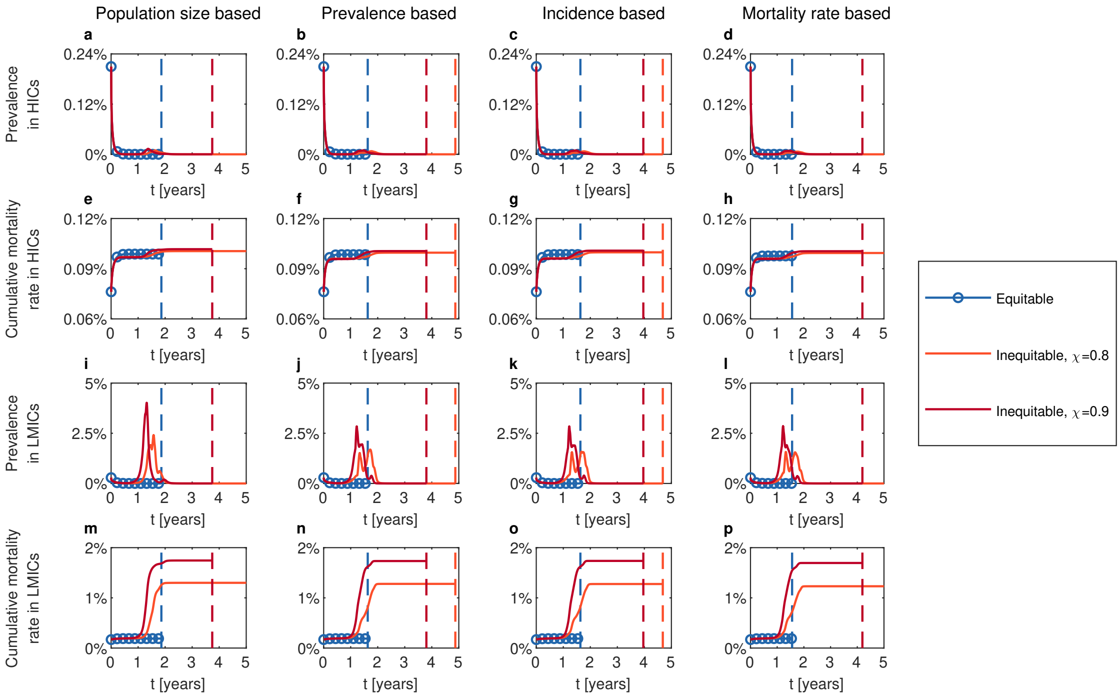

Supplementary Figure 9: Impact of equitable and inequitable vaccine allocation strategies on epidemic dynamics. **a-h**, Time series of the prevalence (**a-d**) and the cumulative mortality rate (**e-h**) in HICs under different global vaccine allocation strategies. **i-p**, Time series of the prevalence (**i-l**) and the cumulative mortality rate (**m-p**) in LMICs under different global vaccine allocation strategies. Four prioritization criteria for allocation are adopted: the population size (the left panel), prevalence (second left panel), incidence (second right panel), and mortality rate (the right panel). Dash lines indicate the time when the pandemic ends (time exceeding five years is not presented; dashed lines referring to the priority criterion are represented by the same colour. Parameter values $M = 3$, $\mu_1 = 5.6 \times 10^{-5}$, $\theta = 0.5$, and $\lambda = 10^4$.

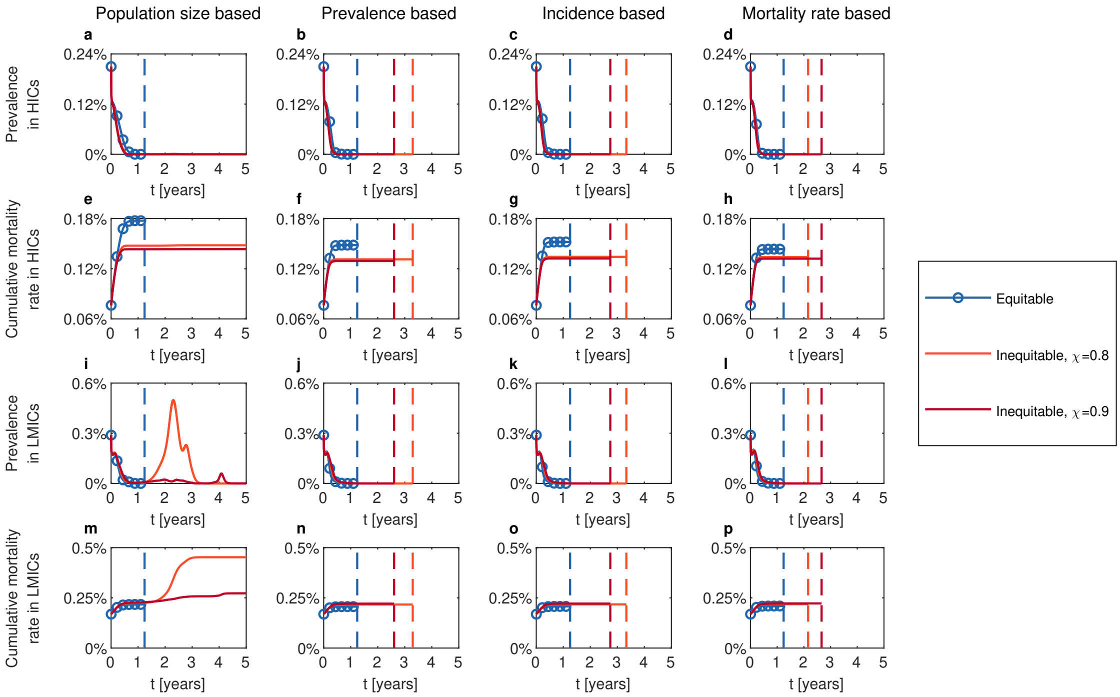

Supplementary Figure 10: Impact of equitable and inequitable vaccine allocation strategies on epidemic dynamics. **a-h**, Time series of the prevalence (**a-d**) and the cumulative mortality rate (**e-h**) in HICs under different global vaccine allocation strategies. **i-p**, Time series of the prevalence (**i-l**) and the cumulative mortality rate (**m-p**) in LMICs under different global vaccine allocation strategies. Four prioritization criteria for allocation are adopted: the population size (the left panel), prevalence (second left panel), incidence (second right panel), and mortality rate (the right panel). Dash lines indicate the time when the pandemic ends (time exceeding five years is not presented; dashed lines referring to the priority criterion are represented by the same colour. Parameter values $M = 5$, $\mu_1 = 5.6 \times 10^{-4}$, $\theta = 0.1$, and $\lambda = 10^3$.

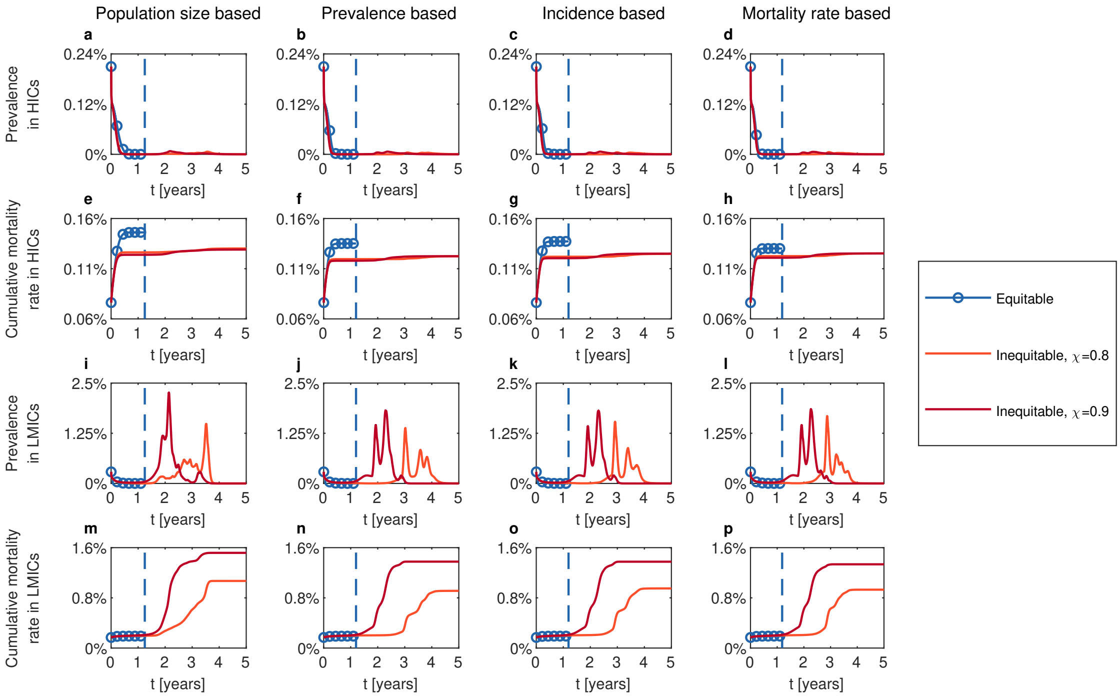

Supplementary Figure 11: Impact of equitable and inequitable vaccine allocation strategies on epidemic dynamics. **a-h**, Time series of the prevalence (**a-d**) and the cumulative mortality rate (**e-h**) in HICs under different global vaccine allocation strategies. **i-p**, Time series of the prevalence (**i-l**) and the cumulative mortality rate (**m-p**) in LMICs under different global vaccine allocation strategies. Four prioritization criteria for allocation are adopted: the population size (the left panel), prevalence (second left panel), incidence (second right panel), and mortality rate (the right panel). Dash lines indicate the time when the pandemic ends (time exceeding five years is not presented; dashed lines referring to the priority criterion are represented by the same colour. Parameter values $M = 4$, $\mu_1 = 5.6 \times 10^{-3}$, $\theta = 0.22$, and $\lambda = 10^2$.

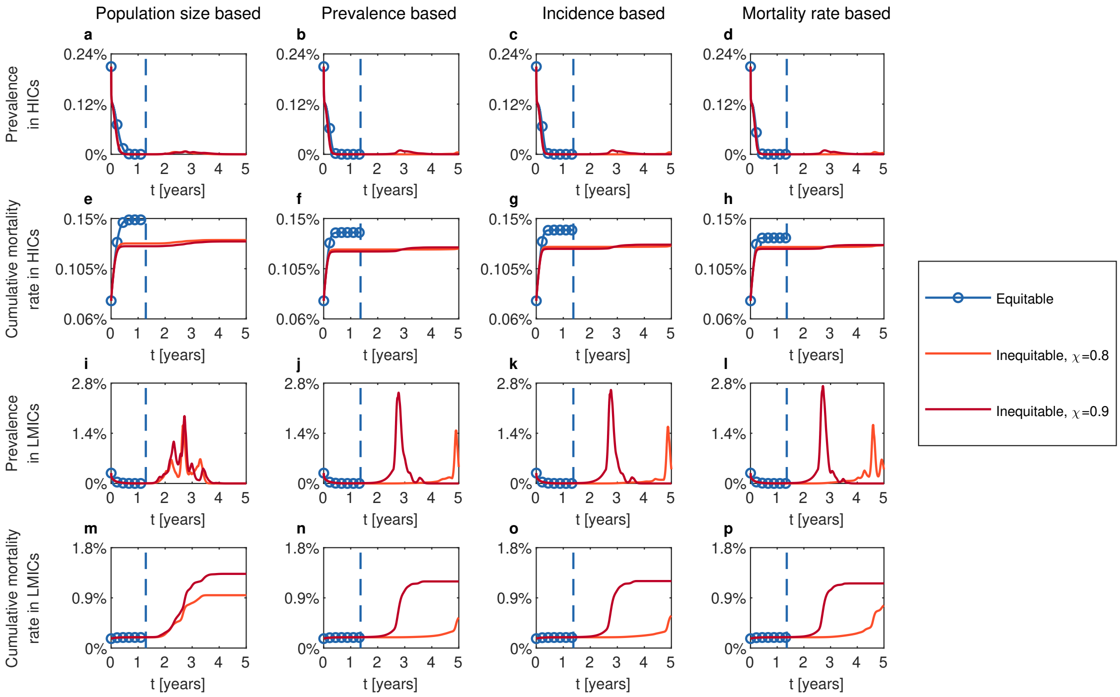

Supplementary Figure 12: Impact of equitable and inequitable vaccine allocation strategies on epidemic dynamics. **a-h**, Time series of the prevalence (**a-d**) and the cumulative mortality rate (**e-h**) in HICs under different global vaccine allocation strategies. **i-p**, Time series of the prevalence (**i-l**) and the cumulative mortality rate (**m-p**) in LMICs under different global vaccine allocation strategies. Four prioritization criteria for allocation are adopted: the population size (the left panel), prevalence (second left panel), incidence (second right panel), and mortality rate (the right panel). Dash lines indicate the time when the pandemic ends (time exceeding five years is not presented; dashed lines referring to the priority criterion are represented by the same colour. Parameter values $M = 3$, $\mu_1 = 5.6 \times 10^{-5}$, $\theta = 0.3$, and $\lambda = 10^4$.

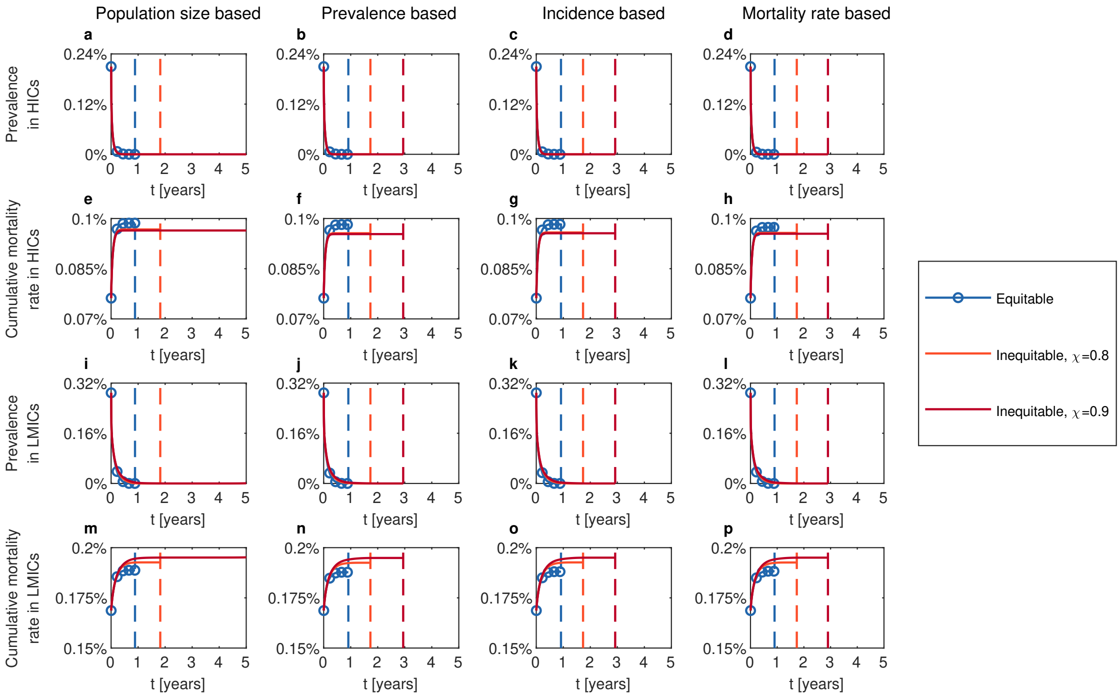

Supplementary Figure 13: Impact of equitable and inequitable vaccine allocation strategies on epidemic dynamics. **a-h**, Time series of the prevalence (**a-d**) and the cumulative mortality rate (**e-h**) in HICs under different global vaccine allocation strategies. **i-p**, Time series of the prevalence (**i-l**) and the cumulative mortality rate (**m-p**) in LMICs under different global vaccine allocation strategies. Four prioritization criteria for allocation are adopted: the population size (the left panel), prevalence (second left panel), incidence (second right panel), and mortality rate (the right panel). Dash lines indicate the time when the pandemic ends (time exceeding five years is not presented; dashed lines referring to the priority criterion are represented by the same colour. Parameter values $M = 10$, $\mu_1 = 5.6 \times 10^{-3}$, $\theta = 0.12$, and $\lambda = 10^3$.

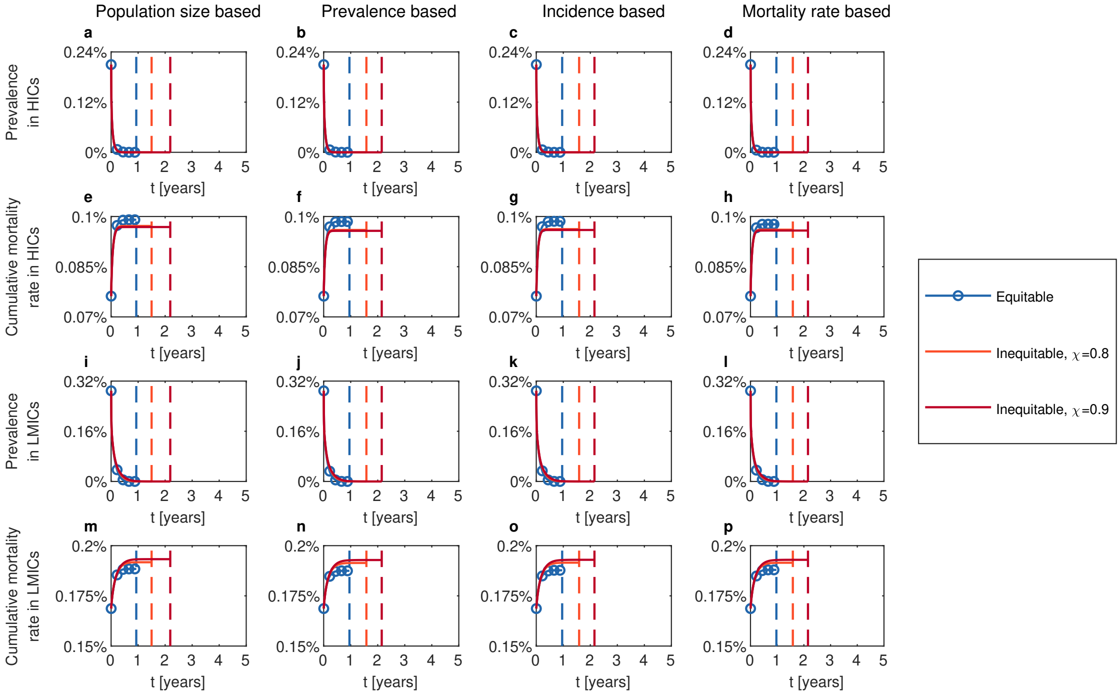

Supplementary Figure 14: Impact of equitable and inequitable vaccine allocation strategies on epidemic dynamics. **a-h**, Time series of the prevalence (**a-d**) and the cumulative mortality rate (**e-h**) in HICs under different global vaccine allocation strategies. **i-p**, Time series of the prevalence (**i-l**) and the cumulative mortality rate (**m-p**) in LMICs under different global vaccine allocation strategies. Four prioritization criteria for allocation are adopted: the population size (the left panel), prevalence (second left panel), incidence (second right panel), and mortality rate (the right panel). Dash lines indicate the time when the pandemic ends (time exceeding five years is not presented; dashed lines referring to the priority criterion are represented by the same colour. Parameter values $M = 9$, $\mu_1 = 5.6 \times 10^{-5}$, $\theta = 0.1$, and $\lambda = 10^2$.

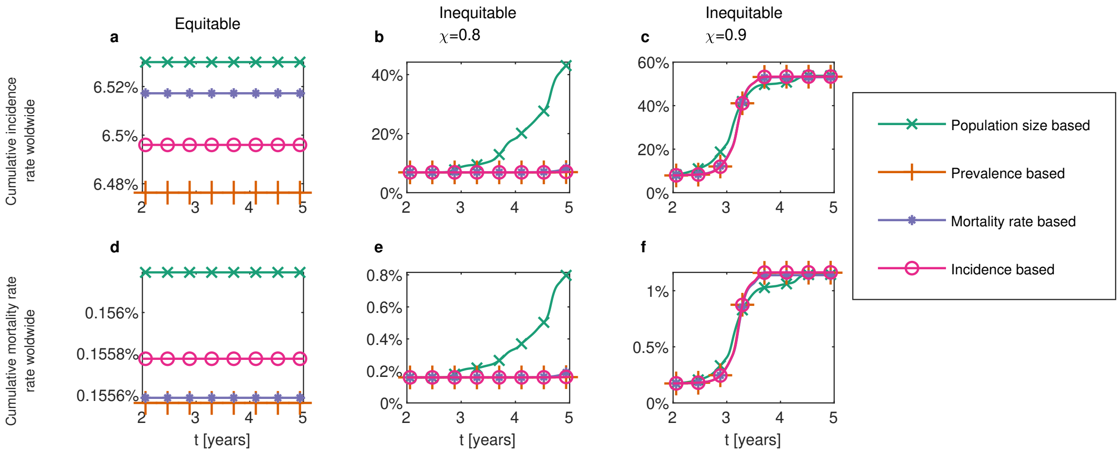

Supplementary Figure 15: Comparison of the impact of four prioritization criteria for global vaccine allocation. **a-c**, Cumulative incidence worldwide under different global vaccine allocation strategies. **d-f**, Cumulative mortality rate worldwide under different global vaccine allocation strategies. Parameters values $M = 5$, $\mu_1 = 5.6 \times 10^{-3}$, $\theta = 0.2$, and $\lambda = 5 \times 10^2$.

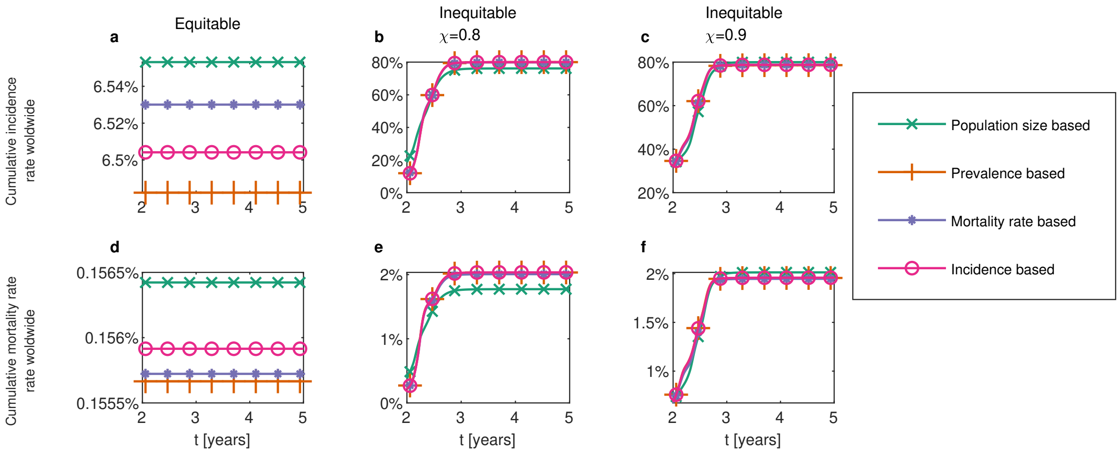

Supplementary Figure 16: Comparison of the impact of four prioritization criteria for global vaccine allocation. **a-c**, Cumulative incidence worldwide under different global vaccine allocation strategies. **d-f**, Cumulative mortality rate worldwide under different global vaccine allocation strategies. Parameters values $M = 6$, $\mu_1 = 5.6 \times 10^{-3}$, $\theta = 0.26$, and $\lambda = 10^2$.

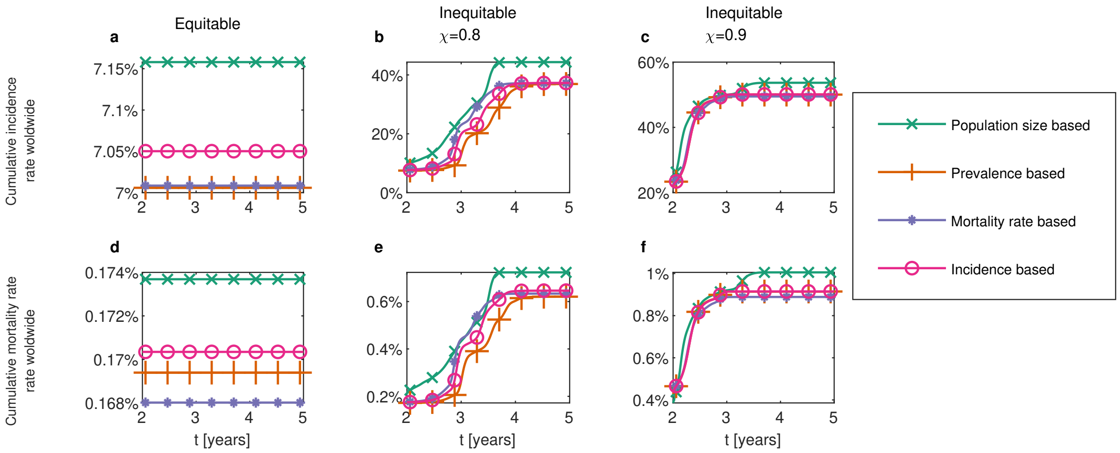

Supplementary Figure 17: Comparison of the impact of four prioritization criteria for global vaccine allocation. **a-c**, Cumulative incidence worldwide under different global vaccine allocation strategies. **d-f**, Cumulative mortality rate worldwide under different global vaccine allocation strategies. Parameters values $M = 4$, $\mu_1 = 5.6 \times 10^{-3}$, $\theta = 0.22$, and $\lambda = 10^2$.

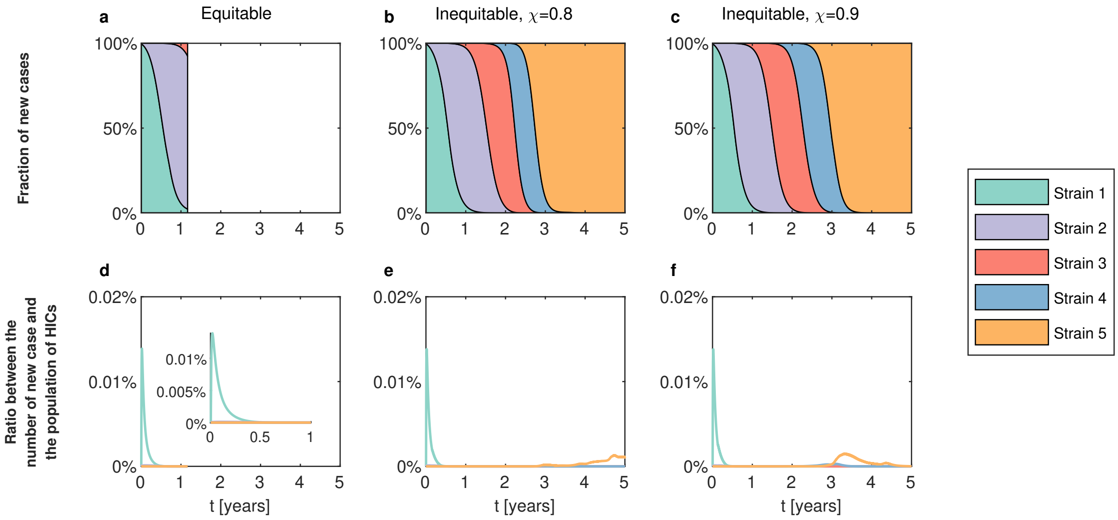

Supplementary Figure 18: Emergence of new strains under equitable and inequitable vaccine allocation strategies. **a-c**, Area plots of the fraction of daily new cases produced by different strains in HICs. **d-f**, The ratio between the number of new cases produced by different strains and the population in HICs. Figures in the left panel, the middle panel, and the right panel are based on the equitable, inequitable and $\chi = 0.8$, and inequitable and $\chi = 0.9$ vaccine allocation strategies, respectively. All results are based on the prioritization criterion of the population size. The inset in subfigure **d** is the zoomed version of subfigure **d**. Parameters values $M = 5$, $\mu_1 = 5.6 \times 10^{-3}$, $\theta = 0.2$, and $\lambda = 5 \times 10^2$.

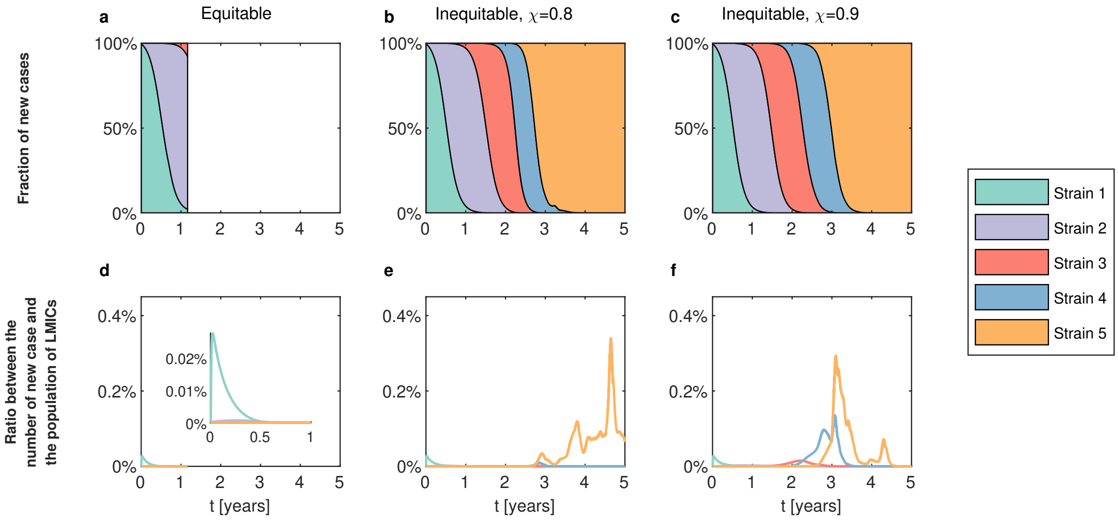

Supplementary Figure 19: Emergence of new strains under equitable and inequitable vaccine allocation strategies. **a-c**, Area plots of the fraction of daily new cases produced by different strains in LMICs. **d-f**, The ratio between the number of new cases produced by different strains and the population in LMICs. Figures in the left panel, the middle panel, and the right panel are based on the equitable, inequitable and $\chi = 0.8$, and inequitable and $\chi = 0.9$ vaccine allocation strategies, respectively. All results are based on the prioritization criterion of the population size. The inset in subfigure **d** is the zoomed version of subfigure **d**. Parameters values $M = 5$, $\mu_1 = 5.6 \times 10^{-3}$, $\theta = 0.2$, and $\lambda = 5 \times 10^2$.

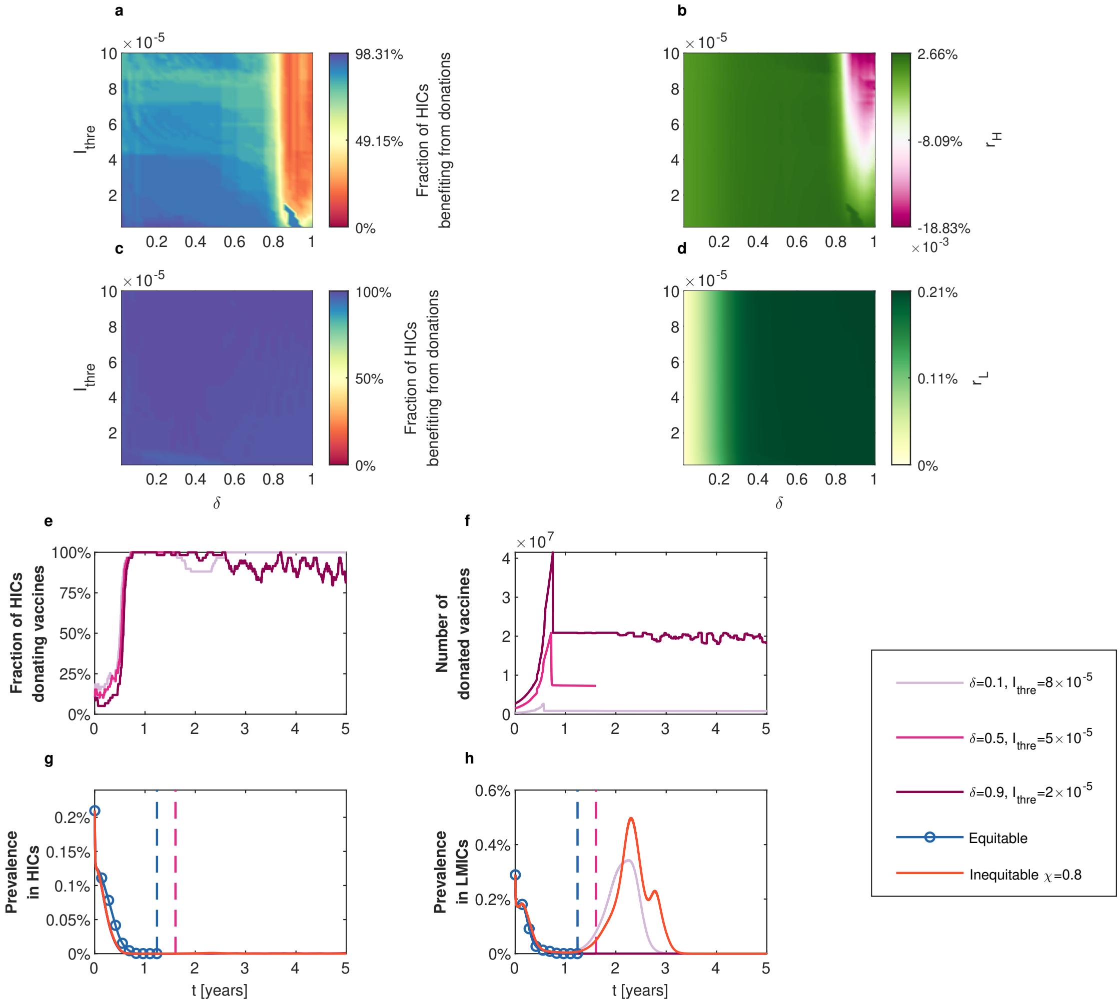

Supplementary Figure 20: Impact of different allow-donation vaccine allocation strategies on epidemic dynamics. **a** and **c**, Fraction of HICs and LMICs benefiting from donations. **b** and **d**, Average lives saved by vaccine donations as the share of the national population in HICs (r_H) and LMICs (r_L). **e**, Fraction of HICs donating vaccines. **f**, Total number of donated vaccines. **g** and **h**, Prevalence in HICs and LMICs under different vaccine allocation strategies. Dash lines indicate the time when the pandemic ends. Countries with larger population sizes are prioritized for vaccination. Parameter values $M = 5$, $\mu_1 = 5.6 \times 10^{-4}$, $\theta = 0.1$, and $\lambda = 10^3$.

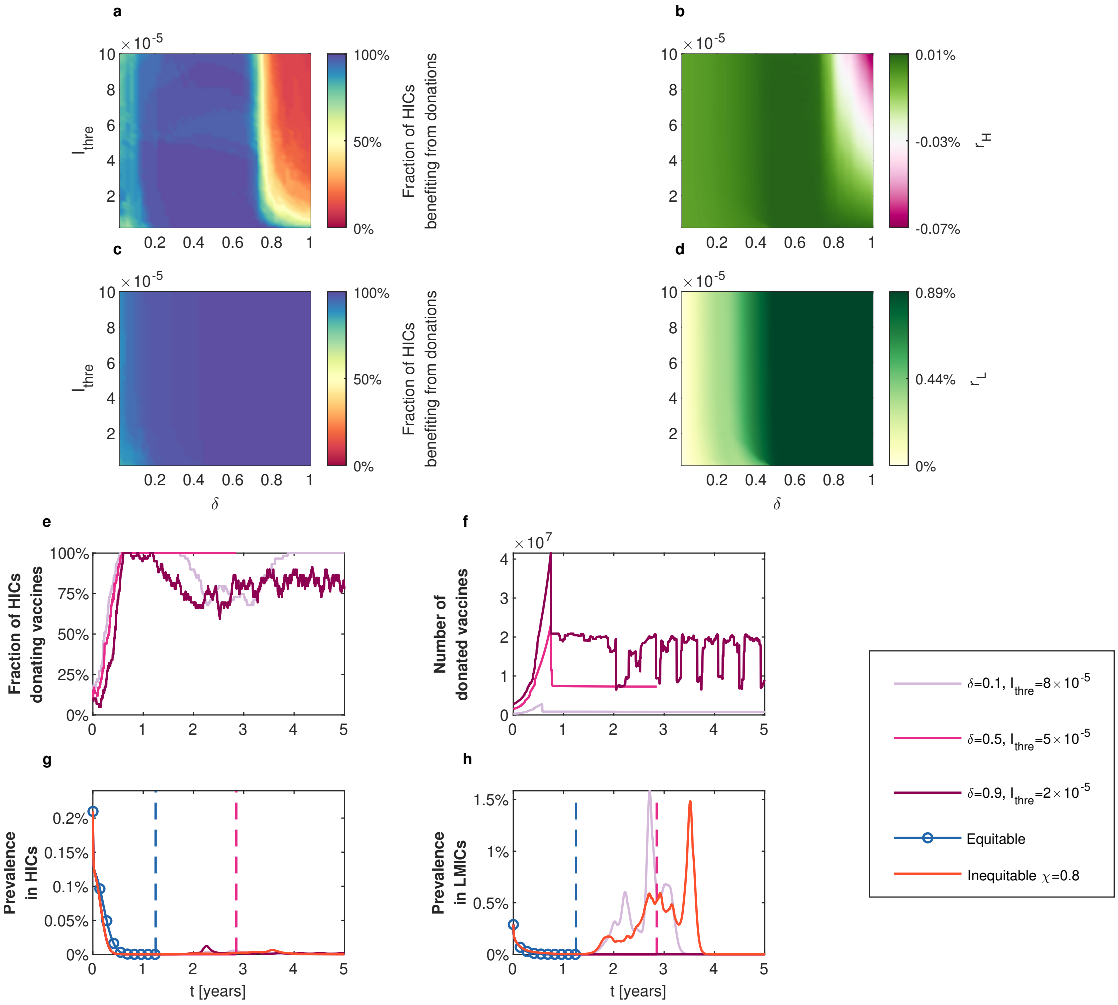

Supplementary Figure 21: Impact of different allow-donation vaccine allocation strategies on epidemic dynamics. **a** and **c**, Fraction of HICs and LMICs benefiting from donations. **b** and **d**, Average lives saved by vaccine donations as the share of the national population in HICs (r_H) and LMICs (r_L). **e**, Fraction of HICs donating vaccines. **f**, Total number of donated vaccines. **g** and **h**, Prevalence in HICs and LMICs under different vaccine allocation strategies. Dash lines indicate the time when the pandemic ends. Countries with larger population sizes are prioritized for vaccination. Parameter values $M = 4$, $\mu_1 = 5.6 \times 10^{-3}$, $\theta = 0.22$, and $\lambda = 10^2$.

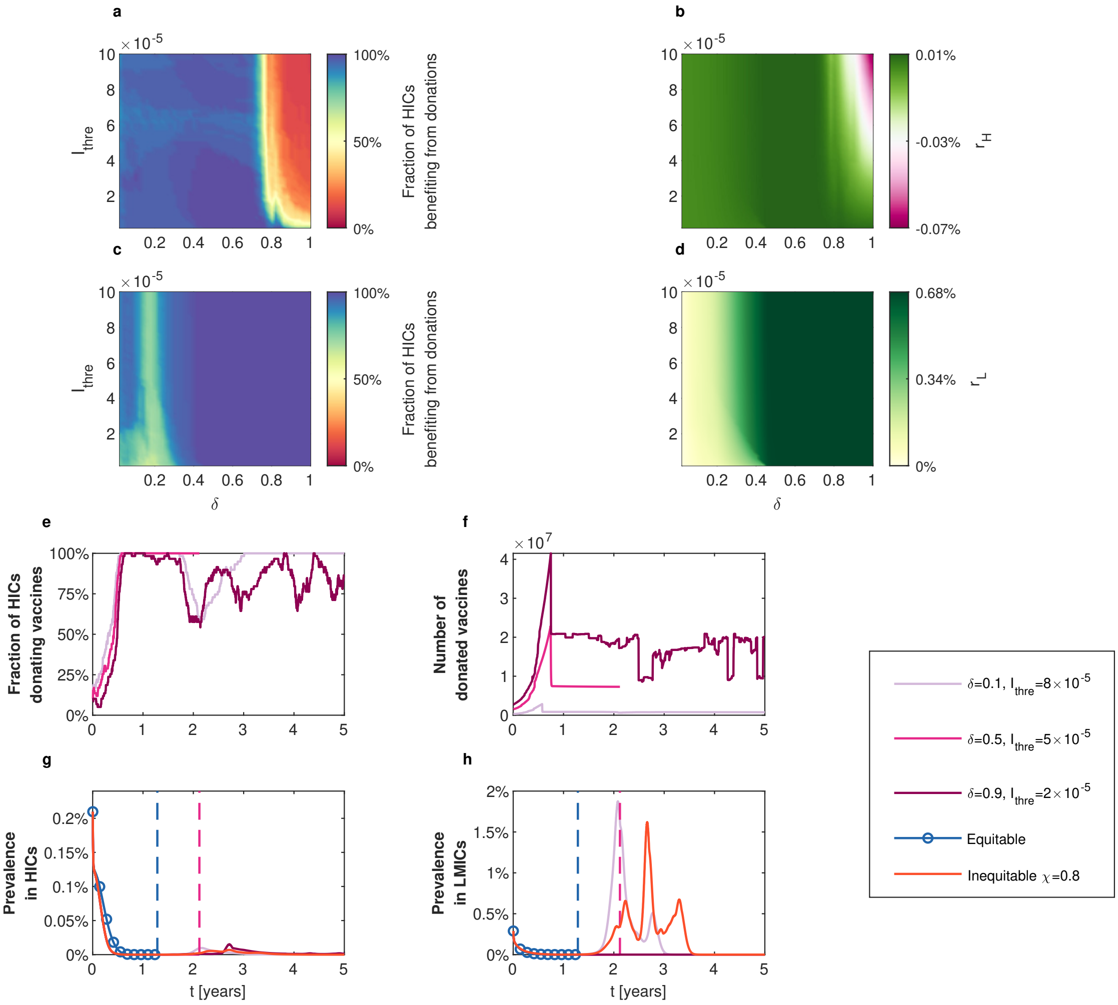

Supplementary Figure 22: Impact of different allow-donation vaccine allocation strategies on epidemic dynamics. **a** and **c**, Fraction of HICs and LMICs benefiting from donations. **b** and **d**, Average lives saved by vaccine donations as the share of the national population in HICs (r_H) and LMICs (r_L). **e**, Fraction of HICs donating vaccines. **f**, Total number of donated vaccines. **g** and **h**, Prevalence in HICs and LMICs under different vaccine allocation strategies. Dash lines indicate the time when the pandemic ends. Countries with larger population sizes are prioritized for vaccination. Parameter values $M = 3$, $\mu_1 = 5.6 \times 10^{-5}$, $\theta = 0.3$, and $\lambda = 10^4$.

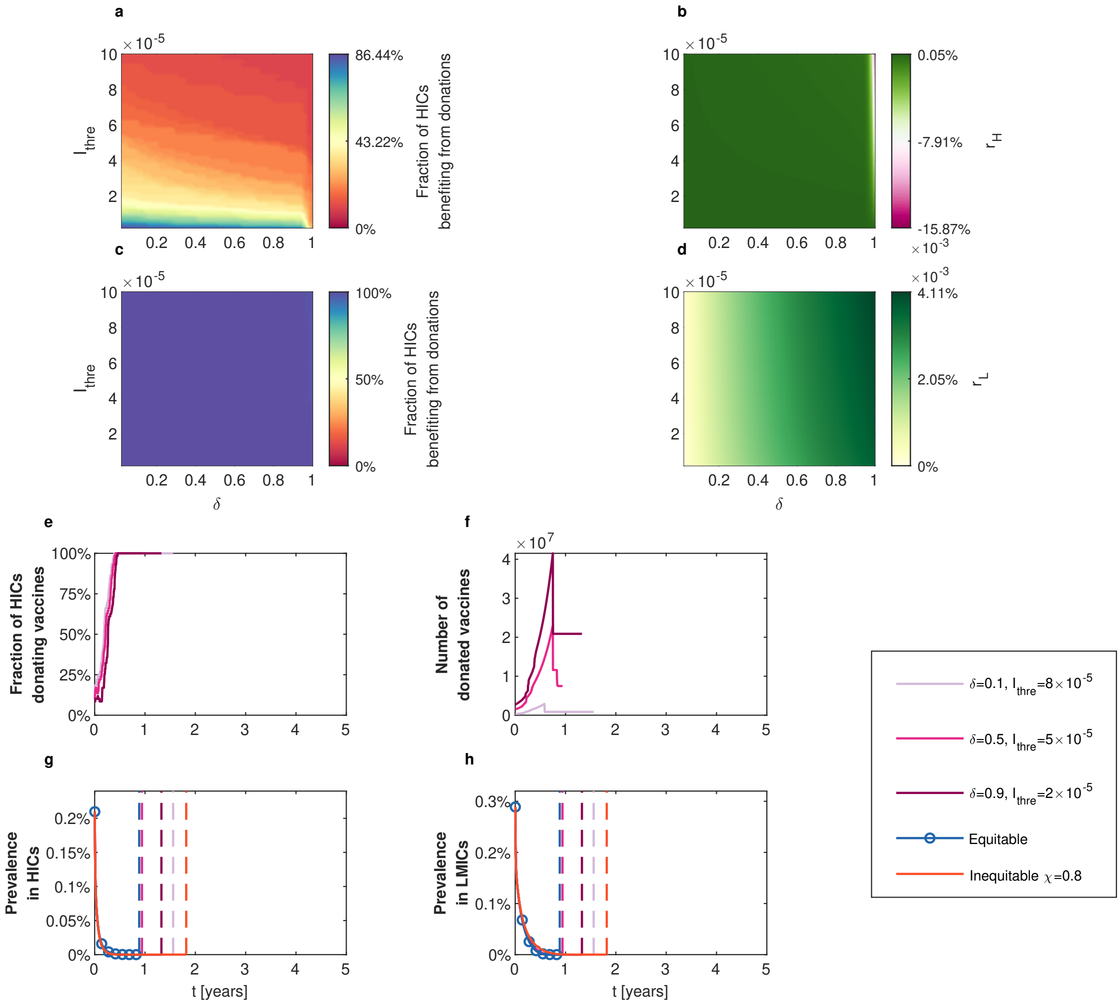

Supplementary Figure 23: Impact of different allow-donation vaccine allocation strategies on epidemic dynamics. **a** and **c**, Fraction of HICs and LMICs benefiting from donations. **b** and **d**, Average lives saved by vaccine donations as the share of the national population in HICs (r_H) and LMICs (r_L). **e**, Fraction of HICs donating vaccines. **f**, Total number of donated vaccines. **g** and **h**, Prevalence in HICs and LMICs under different vaccine allocation strategies. Dash lines indicate the time when the pandemic ends. Countries with larger population sizes are prioritized for vaccination. Parameter values $M = 10$, $\mu_1 = 5.6 \times 10^{-3}$, $\theta = 0.12$, and $\lambda = 10^3$.

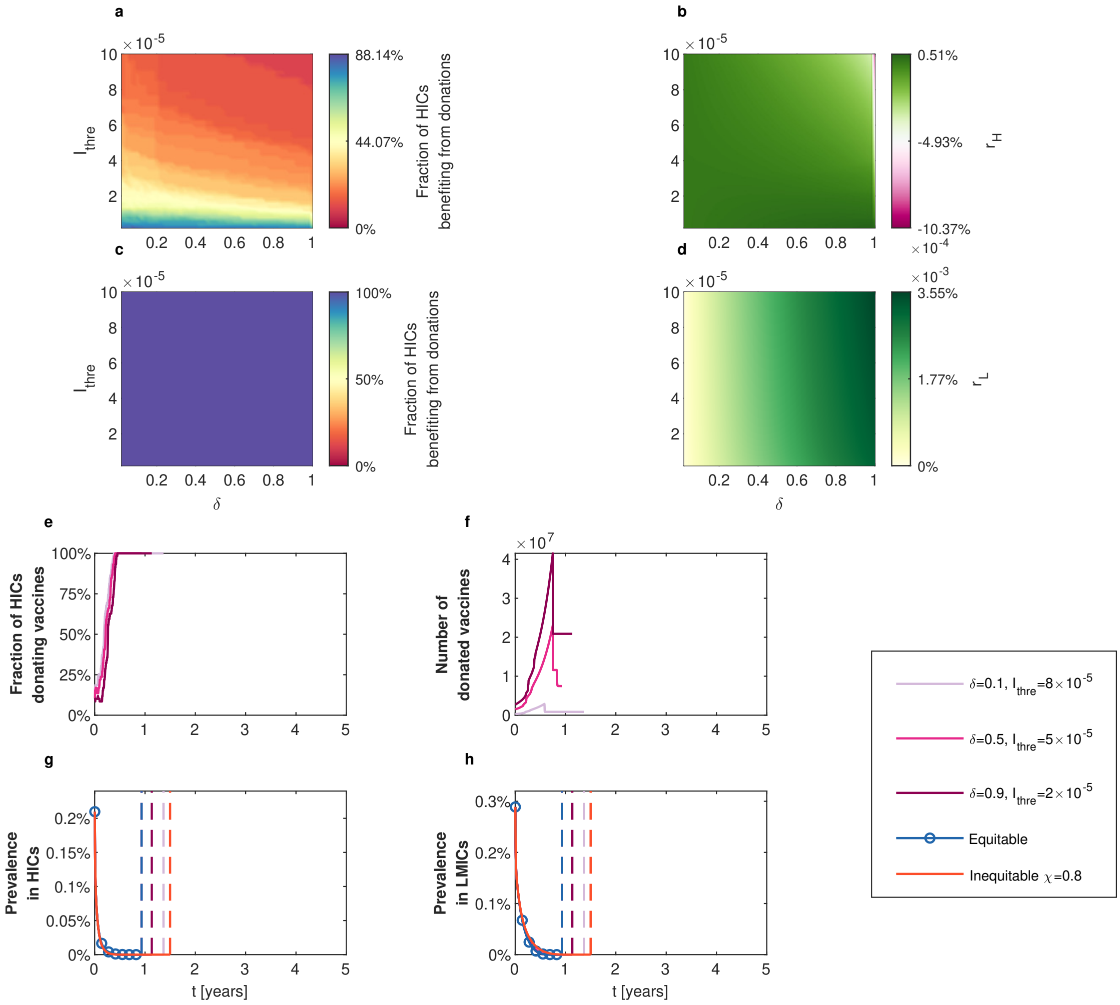

Supplementary Figure 24: Impact of different allow-donation vaccine allocation strategies on epidemic dynamics. **a** and **c**, Fraction of HICs and LMICs benefiting from donations. **b** and **d**, Average lives saved by vaccine donations as the share of the national population in HICs (r_H) and LMICs (r_L). **e**, Fraction of HICs donating vaccines. **f**, Total number of donated vaccines. **g** and **h**, Prevalence in HICs and LMICs under different vaccine allocation strategies. Dash lines indicate the time when the pandemic ends. Countries with larger population sizes are prioritized for vaccination. Parameter values $M = 9$, $\mu_1 = 5.6 \times 10^{-5}$, $\theta = 0.1$, and $\lambda = 10^2$.

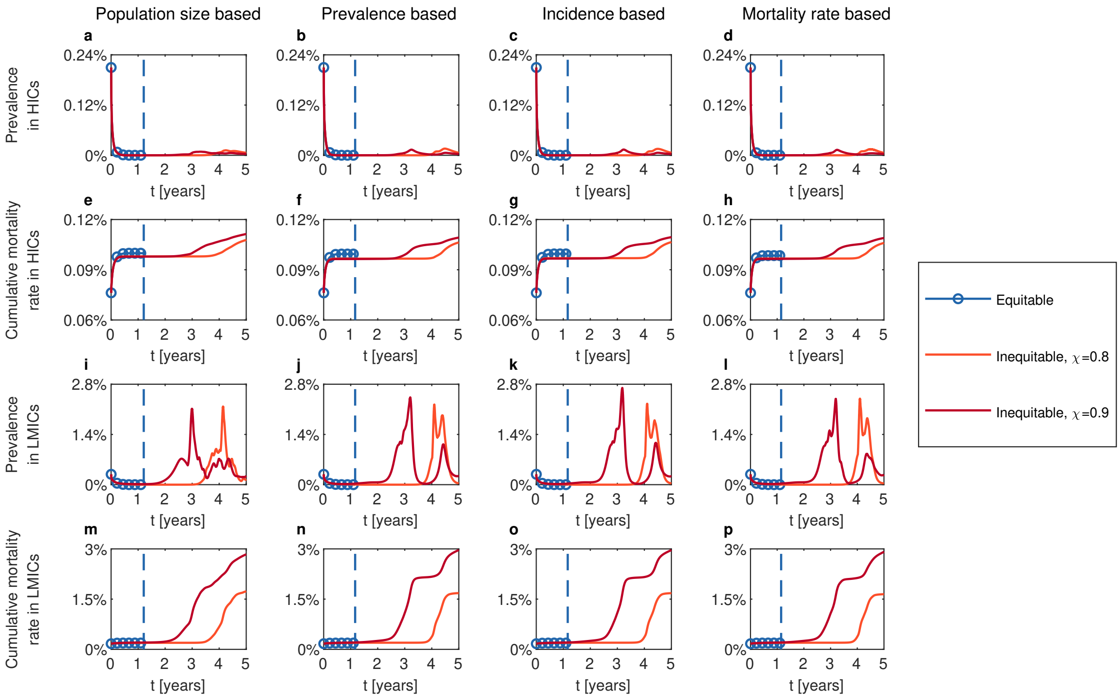

Supplementary Figure 25: Impact of equitable and inequitable vaccine allocation strategies on epidemic dynamics. **a-h**, Time series of the prevalence (**a-d**) and the cumulative mortality rate (**e-h**) in HICs under different global vaccine allocation strategies. **i-p**, Time series of the prevalence (**i-l**) and the cumulative mortality rate (**m-p**) in LMICs under different global vaccine allocation strategies. Four prioritization criteria for allocation are adopted: the population size (the left panel), prevalence (second left panel), incidence (second right panel), and mortality rate (the right panel). Dash lines indicate the time when the pandemic ends (time exceeding five years is not presented; dashed lines referring to the priority criterion are represented by the same colour. The duration of natural immunity is two years. Parameter values $M = 5$, $\mu_1 = 5.6 \times 10^{-3}$, $\theta = 0.2$, and $\lambda = 5 \times 10^2$.

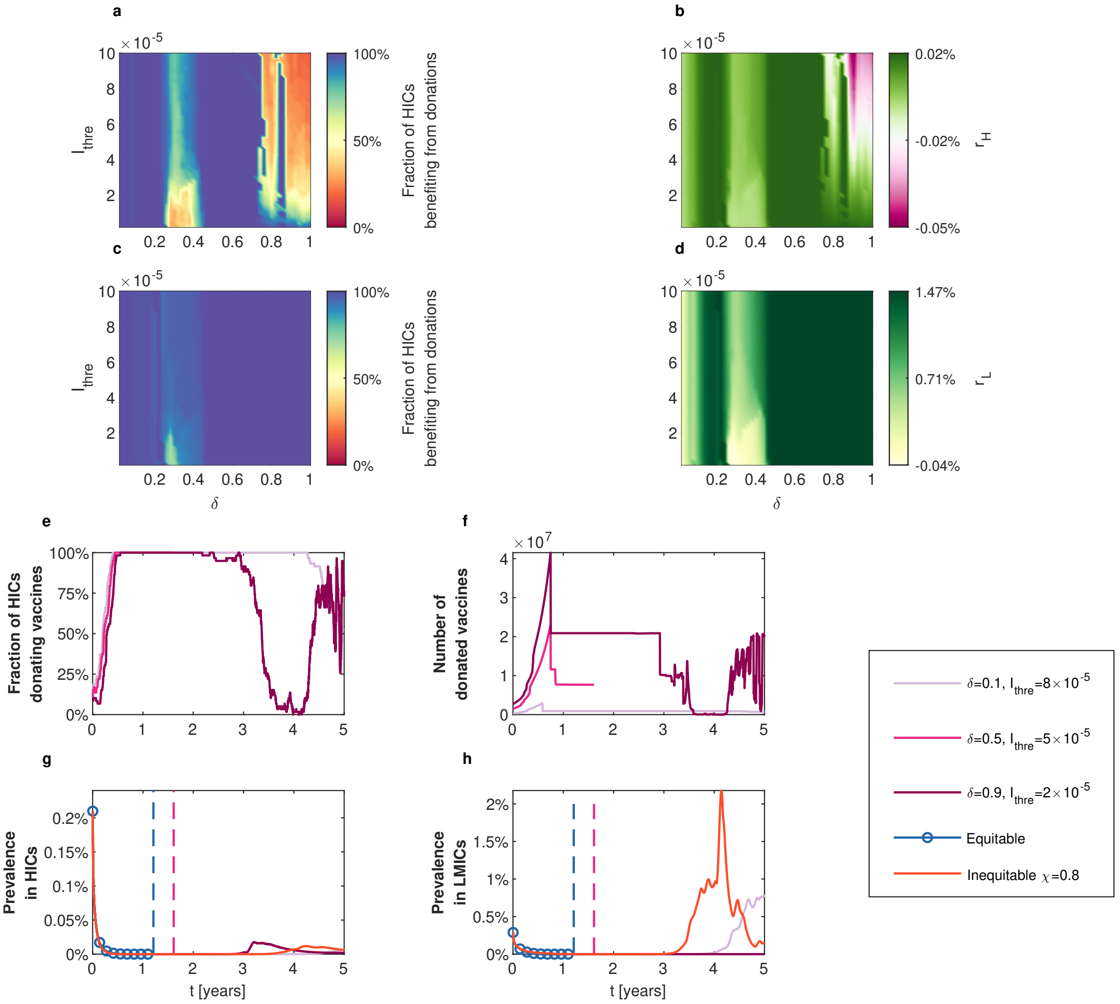

Supplementary Figure 26: Impact of different allow-donation vaccine allocation strategies on epidemic dynamics. **a** and **c**, Fraction of HICs and LMICs benefiting from donations. **b** and **d**, Average lives saved by vaccine donations as the share of the national population in HICs (r_H) and LMICs (r_L). **e**, Fraction of HICs donating vaccines. **f**, Total number of donated vaccines. **g** and **h**, Prevalence in HICs and LMICs under different vaccine allocation strategies. Dash lines indicate the time when the pandemic ends. Countries with larger population sizes are prioritized for vaccination. The duration of natural immunity is two years. Parameter values $M = 5$, $\mu_1 = 5.6 \times 10^{-3}$, $\theta = 0.2$, and $\lambda = 5 \times 10^2$.

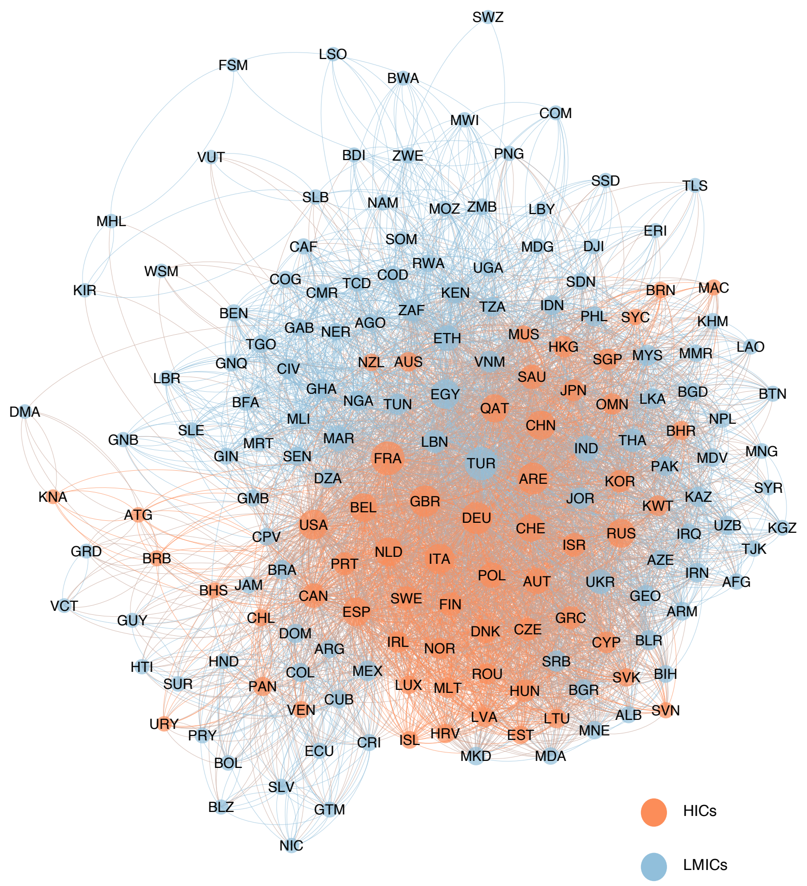

Supplementary Figure 27: The global mobility network. Nodes represent countries/regions. Edges represent the aggregated number of seats on scheduled commercial flights between countries/regions per day. The size of a node is proportional to the number of neighboring countries/regions (countries/regions that are reachable via direct flights). Only 3-letter ISO codes for countries/regions are presented for a clear illustration.

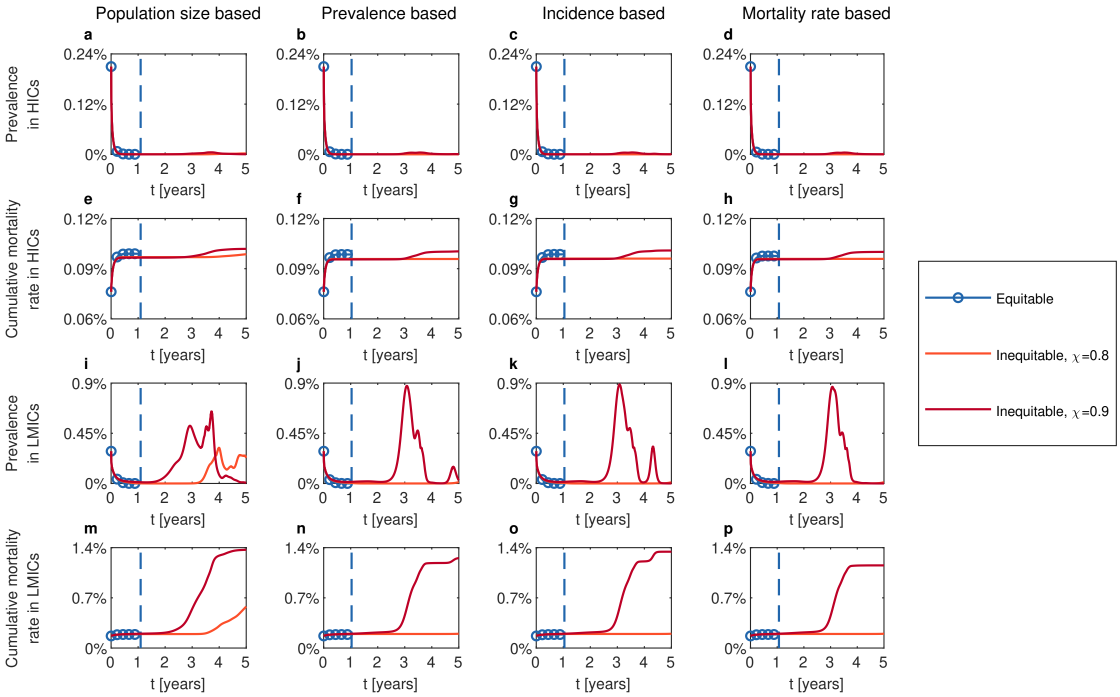

Supplementary Figure 28: Impact of equitable and inequitable vaccine allocation strategies on epidemic dynamics. **a-h**, Time series of the prevalence (**a-d**) and the cumulative mortality rate (**e-h**) in HICs under different global vaccine allocation strategies. **i-p**, Time series of the prevalence (**i-l**) and the cumulative mortality rate (**m-p**) in LMICs under different global vaccine allocation strategies. Four prioritization criteria for allocation are adopted: the population size (the left panel), prevalence (second left panel), incidence (second right panel), and mortality rate (the right panel). Dash lines indicate the time when the pandemic ends (time exceeding five years is not presented; dashed lines referring to the priority criterion are represented by the same colour. Stringent NPIs are triggered when the local effective reproduction number exceeds 0.8. Parameter values $M = 5$, $\mu_1 = 5.6 \times 10^{-3}$, $\theta = 0.2$, and $\lambda = 5 \times 10^2$.

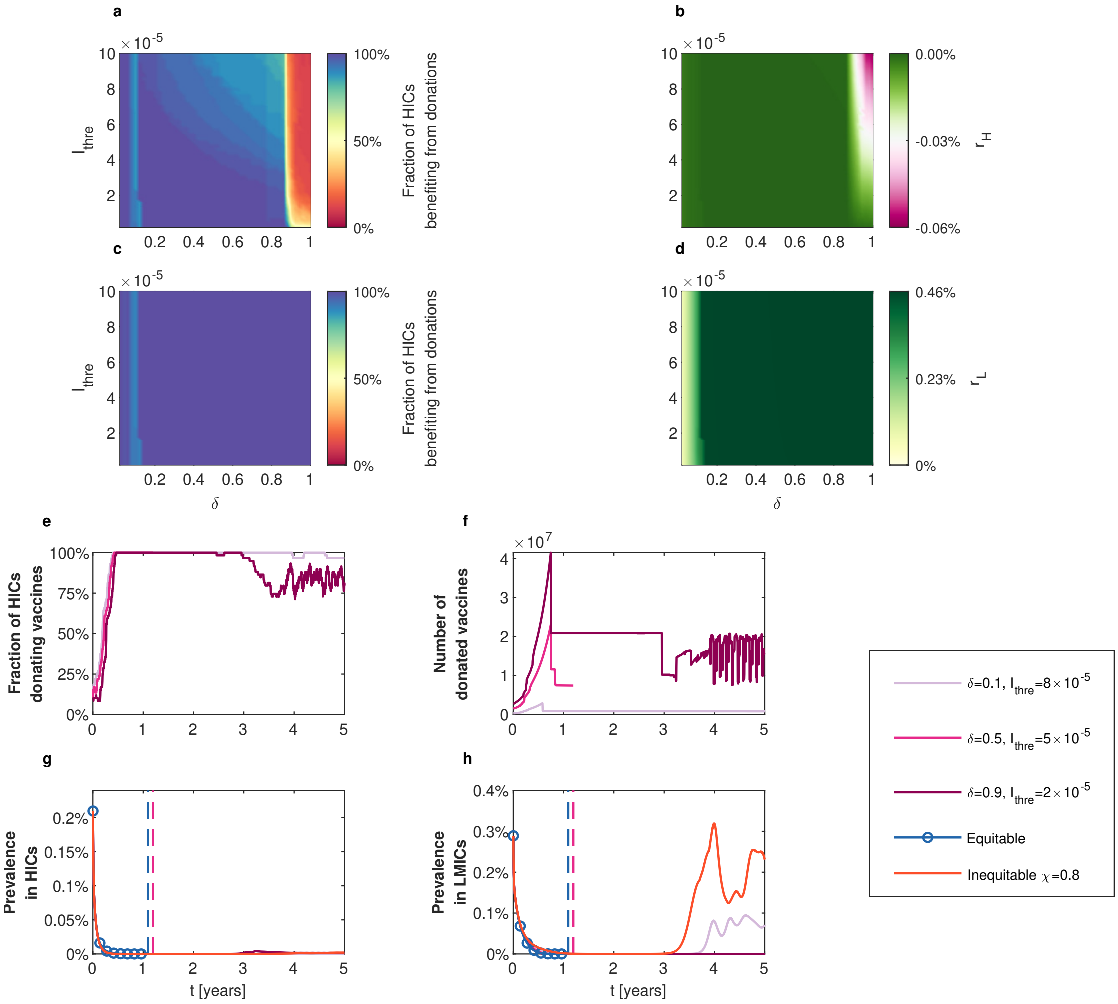

Supplementary Figure 29: Impact of different allow-donation vaccine allocation strategies on epidemic dynamics. **a** and **c**, Fraction of HICs and LMICs benefiting from donations. **b** and **d**, Average lives saved by vaccine donations as the share of the national population in HICs (r_H) and LMICs (r_L). **e**, Fraction of HICs donating vaccines. **f**, Total number of donated vaccines. **g** and **h**, Prevalence in HICs and LMICs under different vaccine allocation strategies. Dash lines indicate the time when the pandemic ends. Countries with larger population sizes are prioritized for vaccination. Stringent NPIs are triggered when the local effective reproduction number exceeds 0.8. Parameter values $M = 5$, $\mu_1 = 5.6 \times 10^{-3}$, $\theta = 0.2$, and $\lambda = 5 \times 10^2$.

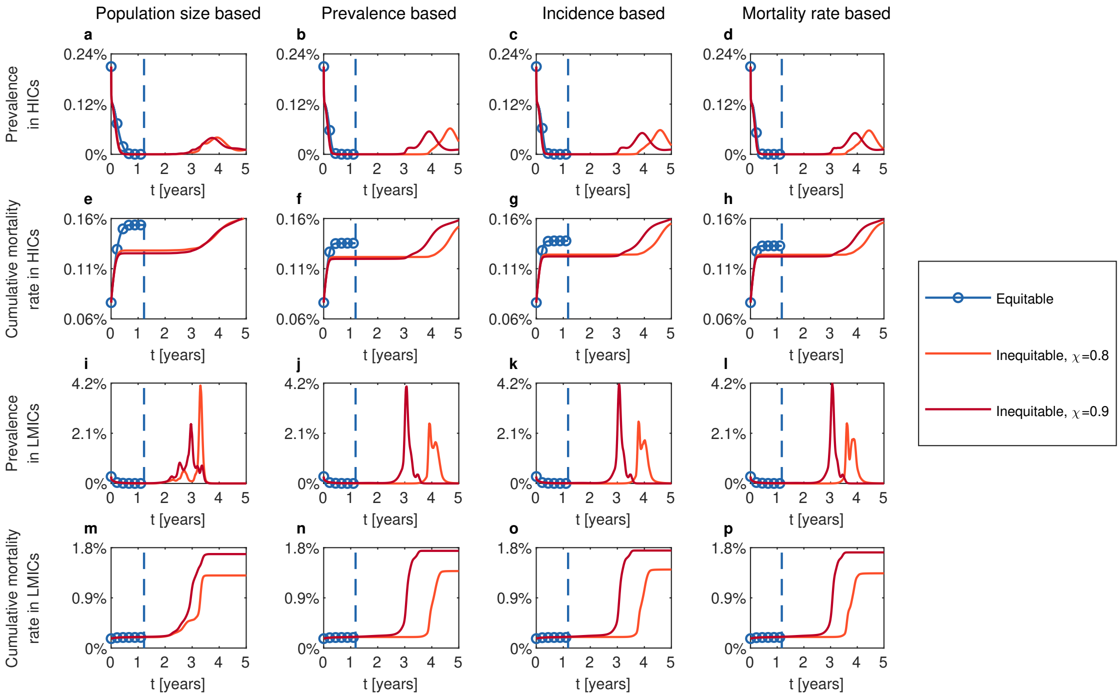

Supplementary Figure 30: Impact of equitable and inequitable vaccine allocation strategies on epidemic dynamics. **a-h**, Time series of the prevalence (**a-d**) and the cumulative mortality rate (**e-h**) in HICs under different global vaccine allocation strategies. **i-p**, Time series of prevalence (**i-l**) and the cumulative mortality rate (**m-p**) in LMICs under different global vaccine allocation strategies. Four prioritization criteria for allocation are adopted: the population size (the left panel), prevalence (second left panel), incidence (second right panel), and mortality rate (the right panel). Dash lines indicate the time when the pandemic ends (time exceeding five years is not presented; dashed lines referring to the priority criterion are represented by the same colour. Stringent NPIs are triggered when the local effective reproduction number exceeds 1.2. Parameter values $M = 5$, $\mu_1 = 5.6 \times 10^{-3}$, $\theta = 0.2$, and $\lambda = 5 \times 10^2$.

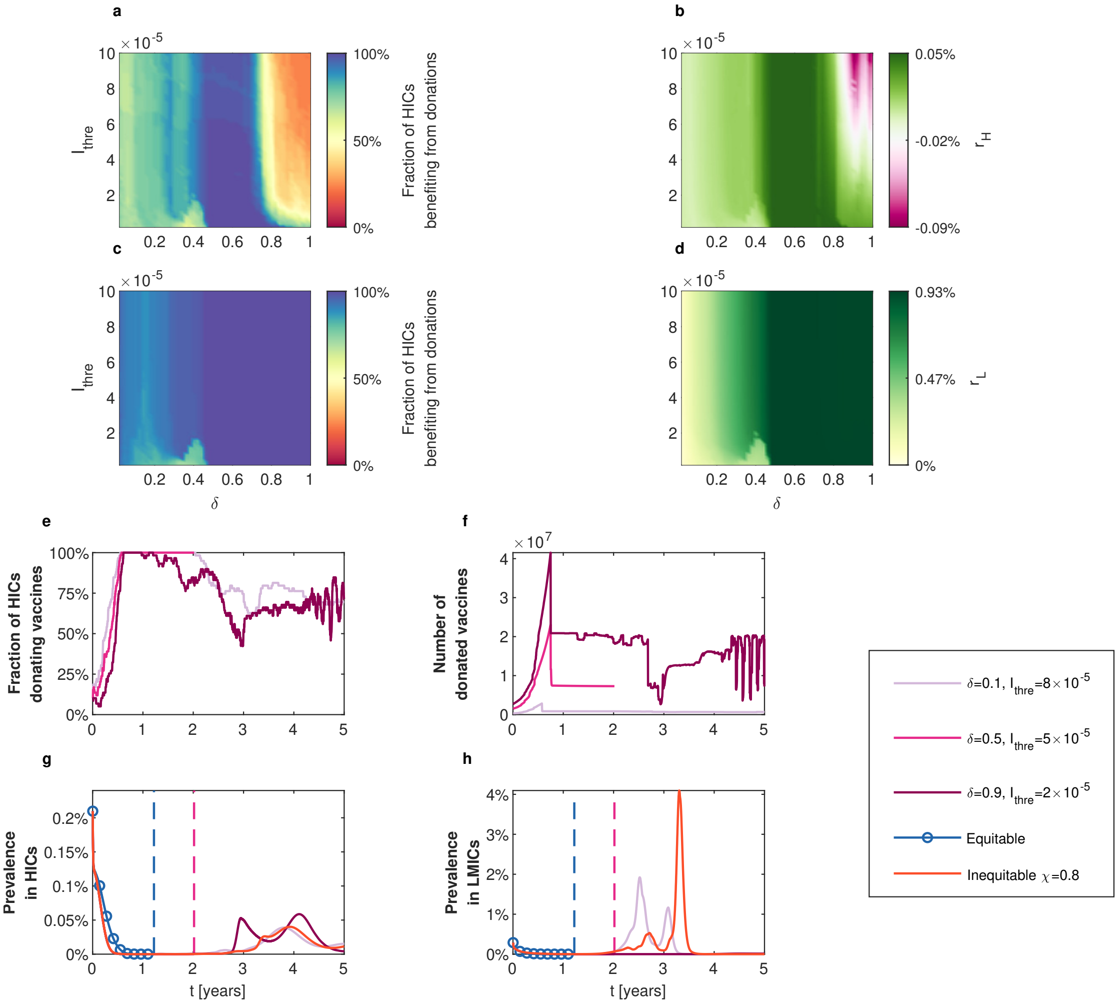

Supplementary Figure 31: Impact of different allow-donation vaccine allocation strategies on epidemic dynamics. **a** and **c**, Fraction of HICs and LMICs benefiting from donations. **b** and **d**, Average lives saved by vaccine donations as the share of the national population in HICs (r_H) and LMICs (r_L). **e**, Fraction of HICs donating vaccines. **f**, Total number of donated vaccines. **g** and **h**, Prevalence in HICs and LMICs under different vaccine allocation strategies. Dash lines indicate the time when the pandemic ends. Countries with larger population sizes are prioritized for vaccination. Stringent NPIs are triggered when the local effective reproduction number exceeds 1.2. Parameter values $M = 5$, $\mu_1 = 5.6 \times 10^{-3}$, $\theta = 0.2$, and $\lambda = 5 \times 10^2$.

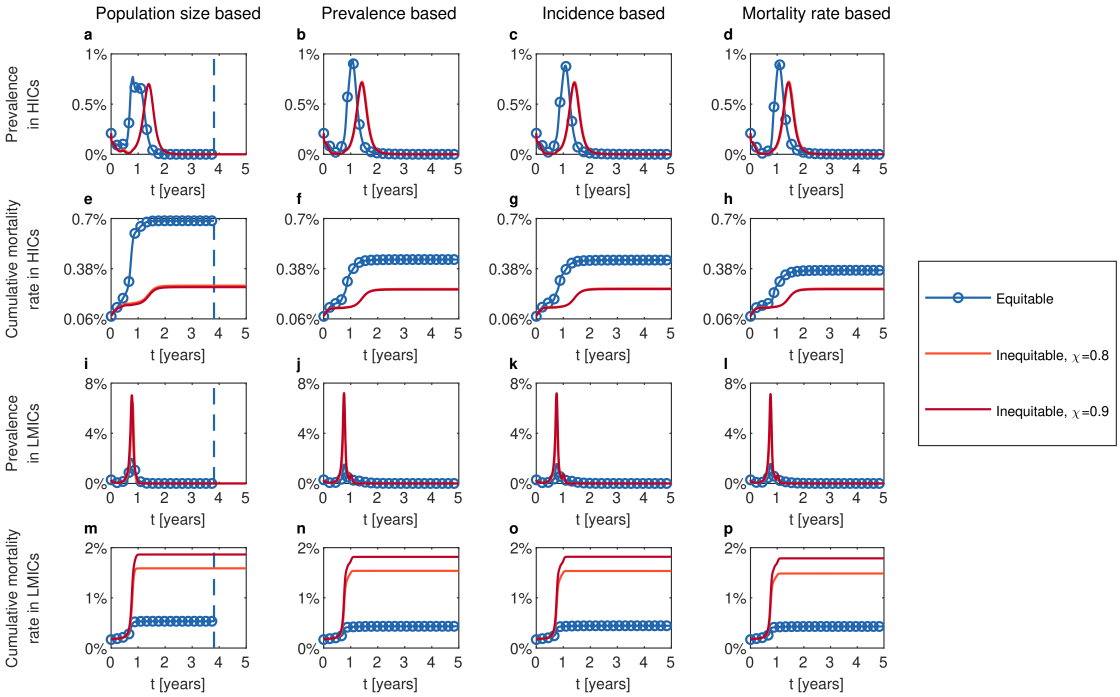

Supplementary Figure 32: Impact of equitable and inequitable vaccine allocation strategies on epidemic dynamics. **a-h**, Time series of the prevalence (**a-d**) and the cumulative mortality rate (**e-h**) in HICs under different global vaccine allocation strategies. **i-p**, Time series of the prevalence (**i-l**) and the cumulative mortality rate (**m-p**) in LMICs under different global vaccine allocation strategies. Four prioritization criteria for allocation are adopted: the population size (the left panel), prevalence (second left panel), incidence (second right panel), and mortality rate (the right panel). Dash lines indicate the time when the pandemic ends (time exceeding five years is not presented; dashed lines referring to the priority criterion are represented by the same colour. Stringent NPIs are triggered when the local effective reproduction number exceeds 1.4. Parameter values $M = 3$, $\mu_1 = 5.6 \times 10^{-3}$, $\theta = 0.5$, and $\lambda = 5 \times 10^2$.

Supplementary References

[1] Wu, S. L. *et al.* Substantial underestimation of sars-cov-2 infection in the united states. *Nature communications* **11**, 4507 (2020). https://doi.org/10.1038/s41467-020-18272-4.

[2] Lash, T. L., Fox, M. P., Fink, A. K. *et al. Applying quantitative bias analysis to epidemiologic data* (Springer, 2009), 1 edn. https://doi.org/10.1007/978-0-387-87959-8.

[3] Sethuraman, N., Jeremiah, S. S. & Ryo, A. Interpreting diagnostic tests for SARS-CoV-2. *JAMA* **323**, 2249–2251 (2020). https://doi.org/10.1001/jama.2020.8259.

[4] Kucirka, L. M., Lauer, S. A., Laeyendecker, O., Boon, D. & Lessler, J. Variation in false-negative rate of reverse transcriptase polymerase chain reaction–based SARS-CoV-2 tests by time since exposure. *Annals of Internal Medicine* **173**, 262–267 (2020). https://doi.org/10.7326/M20-1495.

[5] Corman, V. M. *et al.* Detection of 2019 novel coronavirus (2019-nCoV) by real-time RT-PCR. *Eurosurveillance* **25**, 2000045 (2020). https://doi.org/10.2807/1560-7917.ES.2020.25.3.2000045.

[6] Republic of South Africa Department of Health. *Guidelines for case-finding, diagnosis, and public health response in South Africa* (2020). https://www.nicd.ac.za/wp-content/uploads/2020/09/Guidelines-for-case-finding-diagnosis-and-public-health-response-in-South-Africa_18Aug2020.pdf#page=10.

[7] Indian Council of Medical Research. *Advisory for COVID-19 testing during the second wave of the pandemic* (2021). https://www.icmr.gov.in/pdf/covid/strategy/Advisory_COVID_Testing_in_Second_Wave_04052021.pdf.

[8] Australian Government Department of Health. *Testing Framework for COVID-19 in Australia* (2021). https://www.health.gov.au/sites/default/files/documents/2021/06/coronavirus-covid-19-testing-framework-for-covid-19-in-australia.pdf.

[9] World Health Organization. *Recommendations for national SARS-CoV-2 testing strategies and diagnostic capacities* (2021). https://apps.who.int/iris/bitstream/handle/10665/342002/WHO-2019-nCoV-lab-testing-2021.1-eng.pdf?sequence=1&isAllowed=y.

[10] Laxminarayan, R. *et al.* SARS-CoV-2 infection and mortality during the first epidemic wave in Madurai, south India: a prospective, active surveillance study. *The Lancet Infectious Diseases* **21**, 1665–1676 (2021). https://doi.org/10.1016/S1473-3099(21)00393-5.

[11] Velumani, A. *et al.* SARS-CoV-2 Seroprevalence in 12 Cities of India from July-December 2020. *medRxiv* (2021). https://doi.org/10.1101/2021.03.19.21253429.

[12] Sayampanathan, A. A. *et al.* Infectivity of asymptomatic versus symptomatic COVID-19. *The Lancet* **397**, 93–94 (2021). https://doi.org/10.1016/S0140-6736(20)32651-9.

[13] Jha, S. *et al.* Prevalence of flu-like symptoms and COVID-19 in healthcare workers from India. *Journal of Association Physician of India* **68**, 27–29 (2020). PMID: 32602677.

[14] Goldblatt, D. *et al.* Cross-sectional prevalence of SARS-CoV-2 antibodies in healthcare workers in paediatric facilities in eight countries. *Journal of Hospital Infection* **110**, 60–66 (2021). https://doi.org/10.1016/j.jhin.2020.12.019.

[15] Hasell, J. *et al.* A cross-country database of COVID-19 testing. *Scientific Data* **7**, 345 (2020). https://doi.org/10.1038/s41597-020-00688-8.

[16] Dong, E., Du, H. & Gardner, L. An interactive web-based dashboard to track COVID-19 in real time. *The Lancet infectious diseases* **20**, 533–534 (2020). https://doi.org/10.1016/S1473-3099(20)30120-1.

[17] Yang, J. *et al.* Despite vaccination, China needs non-pharmaceutical interventions to prevent widespread outbreaks of COVID-19 in 2021. *Nature Human Behaviour* **5**, 1009–1020 (2021). https://doi.org/10.1038/s41562-021-01155-z.

[18] Flaxman, S. *et al.* Estimating the effects of non-pharmaceutical interventions on COVID-19 in Europe. *Nature* **584**, 257–261 (2020). https://doi.org/10.1038/s41586-020-2405-7.

[19] Saad-Roy, C. M. *et al.* Immune life history, vaccination, and the dynamics of SARS-CoV-2 over the next 5 years. *Science* **370**, 811–818 (2020). https://doi.org/10.1126/science.abd7343.

[20] Diekmann, O., Heesterbeek, J. A. P. & Metz, J. A. On the definition and the computation of the basic reproduction ratio R0 in models for infectious diseases in heterogeneous populations. *Journal of Mathematical Biology* **28**, 365–382 (1990). https://doi.org/10.1007/BF00178324.

[21] Laxminarayan, R. *et al.* Epidemiology and transmission dynamics of COVID-19 in two Indian states. *Science* **370**, 691–697 (2020). https://doi.org/10.1126/science.abd7672.

[22] Hu, B., Guo, H., Zhou, P. & Shi, Z.-L. Characteristics of SARS-CoV-2 and COVID-19. *Nature Reviews Microbiology* **19**, 141–154 (2021). https://doi.org/10.1038/s41579-020-00459-7.

[23] Baker, R. E., Yang, W., Vecchi, G. A., Metcalf, C. J. E. & Grenfell, B. T. Susceptible supply limits the role of climate in the early SARS-CoV-2 pandemic. *Science* **369**, 315–319 (2020). https://doi.org/10.1126/science.abc2535.

[24] Graham, M. S. *et al.* Changes in symptomatology, reinfection, and transmissibility associated with the SARS-CoV-2 variant B. 1.1. 7: an ecological study. *The Lancet Public Health* **6**, E335–E345 (2021). https://doi.org/10.1016/S2468-2667(21)00055-4.

[25] Ye, Y. *et al.* Impacts of Export Restrictions on the Global Personal Protective Equipment Trade Network During COVID-19. *Advanced Theory and Simulations* 2100352 (2021). https://doi.org/10.1002/adts.202100352.

[26] Anderson, R. M., Vegvari, C., Truscott, J. & Collyer, B. S. Challenges in creating herd immunity to SARS-CoV-2 infection by mass vaccination. *The Lancet* **396**, 1614–1616 (2020). https://doi.org/10.1016/S0140-6736(20)32318-7.

[27] Dan, J. M. *et al.* Immunological memory to SARS-CoV-2 assessed for up to 8 months after infection. *Science* **371**, eabf4063 (2021). https://doi.org/10.1126/science.abf4063.

[28] Shu, Y. & McCauley, J. GISAID: Global initiative on sharing all influenza data–from vision to reality. *Eurosurveillance* **22**, 30494 (2017). https://doi.org/10.2807/1560-7917.ES.2017.22.13.30494.

[29] Burki, T. K. Lifting of COVID-19 restrictions in the UK and the Delta variant. *The Lancet Respiratory Medicine* **9**, e85 (2021). https://doi.org/10.1016/S2213-2600(21)00328-3.

[30] Campbell, F. *et al.* Increased transmissibility and global spread of sars-cov-2 variants of concern as at june 2021. *Eurosurveillance* **26**, 2100509 (2021). https://doi.org/10.2807/1560-7917.ES.2021.26.24.2100509.

[31] Olliaro, P., Torreele, E. & Vaillant, M. COVID-19 vaccine efficacy and effectiveness—the elephant (not) in the room. *The Lancet Microbe* **2**, e279–e280 (2021). https://doi.org/10.1016/S2666-5247(21)00069-0.

[32] Haas, E. J. *et al.* Impact and effectiveness of mRNA BNT162b2 vaccine against SARS-CoV-2 infections and COVID-19 cases, hospitalisations, and deaths following a nationwide vaccination campaign in Israel: an observational study using national surveillance data. *The Lancet* **396**, 1819–1829 (2021). https://doi.org/10.1016/S0140-6736(21)00947-8.

[33] World Health Organization. *Tracking SARS-CoV-2 variants* (2021). https://www.who.int/en/activities/tracking-SARS-CoV-2-variants/.

[34] Rambaut, A. *et al.* A dynamic nomenclature proposal for SARS-CoV-2 lineages to assist genomic epidemiology. *Nature microbiology* **5**, 1403–1407 (2020). https://doi.org/10.1038/s41564-020-0770-5.

[35] Gog, J. R. & Grenfell, B. T. Dynamics and selection of many-strain pathogens. *Proceedings of the National Academy of Sciences* **99**, 17209–17214 (2002). https://doi.org/10.1073/pnas.252512799.

[36] Yang, H.-C. *et al.* Analysis of genomic distributions of SARS-CoV-2 reveals a dominant strain type with strong allelic associations. *Proceedings of the National Academy of Sciences* **117**, 30679–30686 (2020). https://doi.org/10.1073/pnas.2007840117.

[37] Li, Q. *et al.* The impact of mutations in SARS-CoV-2 spike on viral infectivity and antigenicity. *Cell* **182**, 1284–1294 (2020). https://doi.org/10.1016/j.cell.2020.07.012.

[38] Frampton, D. *et al.* Genomic characteristics and clinical effect of the emergent SARS-CoV-2 B. 1.1. 7 lineage in London, UK: a whole-genome sequencing and hospital-based cohort study. *The Lancet Infectious Diseases* **21**, 1246–1256 (2021). https://doi.org/10.1016/S1473-3099(21)00170-5.

[39] Sender, R. *et al.* The total number and mass of SARS-CoV-2 virions. *Proceedings of the National Academy of Sciences* **118**, e2024815118 (2021). https://doi.org/10.1073/pnas.2024815118.

[40] Burioni, R. & Topol, E. J. Has SARS-CoV-2 reached peak fitness? *Nature Medicine* **27**, 1323–1324 (2021). https://doi.org/10.1038/s41591-021-01421-7.

[41] Kupferschmidt, K. Evolving threat. *Science* **373**, 844–849 (2021). https://doi.org/10.1126/science.373.6557.844.
