# Supplementary material for: Equitable access to COVID-19 vaccines makes a life-saving difference to all countries
Source: Nat Hum Behav. 2022 Jan 31;6(2):207–16. doi: 10.1038/s41562-022-01289-8 (PMC8873023; doi:10.1038/s41562-022-01289-8)
Supplement: Supplementary file 2 — Reporting Summary [file 41562_2022_1289_MOESM2_ESM.pdf]

nature portfolio

Corresponding author(s): Qingpeng Zhang and Daniel Dajun Zeng

Last updated by author(s): Dec 31, 2021

Reporting Summary

Nature Portfolio wishes to improve the reproducibility of the work that we publish. This form provides structure for consistency and transparency in reporting. For further information on Nature Portfolio policies, see our Editorial Policies and the Editorial Policy Checklist.

Statistics

For all statistical analyses, confirm that the following items are present in the figure legend, table legend, main text, or Methods section.

n/a	Confirmed	
☐	☒	The exact sample size (n) for each experimental group/condition, given as a discrete number and unit of measurement
☒	☐	A statement on whether measurements were taken from distinct samples or whether the same sample was measured repeatedly
☒	☐	The statistical test(s) used AND whether they are one- or two-sided<br>*Only common tests should be described solely by name; describe more complex techniques in the Methods section.*
☒	☐	A description of all covariates tested
☒	☐	A description of any assumptions or corrections, such as tests of normality and adjustment for multiple comparisons
☐	☒	A full description of the statistical parameters including central tendency (e.g. means) or other basic estimates (e.g. regression coefficient) AND variation (e.g. standard deviation) or associated estimates of uncertainty (e.g. confidence intervals)
☒	☐	For null hypothesis testing, the test statistic (e.g. F, t, r) with confidence intervals, effect sizes, degrees of freedom and P value noted<br>*Give P values as exact values whenever suitable.*
☐	☒	For Bayesian analysis, information on the choice of priors and Markov chain Monte Carlo settings
☒	☐	For hierarchical and complex designs, identification of the appropriate level for tests and full reporting of outcomes
☒	☐	Estimates of effect sizes (e.g. Cohen's d, Pearson's r), indicating how they were calculated

Our web collection on statistics for biologists contains articles on many of the points above.

Software and code

Policy information about availability of computer code

Data collection	No software was used to collected data.
Data analysis	All simulations were implemented in Python (version 3.7.1). All simulation results are visualized in Matlab (version 9.5.0.1049112 (R2018b) Update 3). All processed data and code have been deposited in Zenodo (https://doi.org/10.5281/zenodo.5810400) and can also be accessed at https://github.com/jianan0099/VacEquity.

For manuscripts utilizing custom algorithms or software that are central to the research but not yet described in published literature, software must be made available to editors and reviewers. We strongly encourage code deposition in a community repository (e.g. GitHub). See the Nature Portfolio guidelines for submitting code & software for further information.

Data

Policy information about availability of data

All manuscripts must include a data availability statement. This statement should provide the following information, where applicable:

- Accession codes, unique identifiers, or web links for publicly available datasets
- A description of any restrictions on data availability
- For clinical datasets or third party data, please ensure that the statement adheres to our policy

Country-level epidemiological data are collected from publicly available repositories operated by the Johns Hopkins University Center for Systems Science and Engineering (https://github.com/CSSEGISandData/COVID-19) and Our World in Data (https://github.com/owid/covid-19-data). Country-level income data are obtained from the World Bank (https://data.worldbank.org). Population data are obtained from the United Nations World Population Prospects national estimates (https://population.un.org/wpp/). Global mobility data are commercially available from the Official Aviation Guide (https://www.oag.com/) and are used under

license for the current study. Due to restrictions in the licensing agreement with OAG, these data are not publicly available. Other data we used are documented in the main text. Processed data have been deposited in Zenodo (https://doi.org/10.5281/zenodo.5810400).

Field-specific reporting

Please select the one below that is the best fit for your research. If you are not sure, read the appropriate sections before making your selection.

☐ Life sciences ☒ Behavioural & social sciences ☐ Ecological, evolutionary & environmental sciences

For a reference copy of the document with all sections, see nature.com/documents/nr-reporting-summary-flat.pdf

Behavioural & social sciences study design

All studies must disclose on these points even when the disclosure is negative.

Study description	To exploit a realistic pathway to global vaccine equity with the consideration of evolving COVID-19 strains, we examine a number of vaccine allocation strategies using a multi-strain meta-population model based on real-world quantitative data. This study provides insights into effective response to the next wave of COVID-19 and other emerging infectious diseases.
Research sample	The proposed model considers 179 countries.
Sampling strategy	Countries appear in all datasets are included.
Data collection	Country-level epidemiological data are obtained from publicly available repositories operated by the Johns Hopkins University Center for Systems Science and Engineering and Our World in Data. Country-level income data (gross national income per capita) are obtained from the World Bank Open Data. Population data are obtained from the 2019 version of United Nations World Population Prospects national estimates. Global mobility data are provided by the Official Aviation Guide and are used under license for the current study. The experimental condition and the study hypothesis do not apply to this study.
Timing	The modeling study was performed during March 01 2021 to August 10 2021.
Data exclusions	Data for countries that do not appear in all datasets are excluded.
Non-participation	No participants dropped out or declined participation because this study does not involve individual participation.
Randomization	This is not a randomization study.

Reporting for specific materials, systems and methods

We require information from authors about some types of materials, experimental systems and methods used in many studies. Here, indicate whether each material, system or method listed is relevant to your study. If you are not sure if a list item applies to your research, read the appropriate section before selecting a response.

Materials & experimental systems

n/a	Involved in the study	
☒	☐	Antibodies
☒	☐	Eukaryotic cell lines
☒	☐	Palaeontology and archaeology
☒	☐	Animals and other organisms
☒	☐	Human research participants
☒	☐	Clinical data
☒	☐	Dual use research of concern

Methods

n/a	Involved in the study	
☒	☐	ChIP-seq
☒	☐	Flow cytometry
☒	☐	MRI-based neuroimaging
