# Supplementary material for: Equitable access to COVID-19 vaccines makes a life-saving difference to all countries
Source: Nat Hum Behav. 2022 Jan 31;6(2):207–16. doi: 10.1038/s41562-022-01289-8 (PMC8873023; doi:10.1038/s41562-022-01289-8)
Supplement: Supplementary file 3 — Peer Review Information [file 41562_2022_1289_MOESM3_ESM.pdf]

naturerearch

Peer Review Information

Journal: Nature Human Behaviour
Manuscript Title: Equitable access to COVID-19 vaccines makes a lifesaving difference to all countries.
Corresponding author name(s): Qingpeng Zhang and Daniel Dajun Zeng.

Editorial Notes:

Transferred manuscripts This manuscript has been previously reviewed at another journal that is not operating a transparent peer review scheme. This document only contains reviewer comments, rebuttal and decision letters for versions considered at Nature Human Behaviour.

Reviewer Comments & Decisions

Decision Letter, initial version:

4th October 2021

Dear Professor Zhang,

Thank you once again for your manuscript, entitled "Promoting equitable access to COVID-19 vaccines makes a life-saving difference to all countries", and for your patience during the peer review process.

Your Article has now been evaluated by 3 referees. You will see from their comments copied below that, although they find your work of considerable potential interest, they have raised quite substantial concerns. In light of these comments, we cannot accept the manuscript for publication, but would be interested in considering a revised version if you are willing and able to fully address reviewer and editorial concerns.

We hope you will find the referees' comments useful as you decide how to proceed. If you wish to submit a substantially revised manuscript, please bear in mind that we will be reluctant to approach the referees again in the absence of major revisions. We are committed to providing a fair and constructive peer-review process. Do not hesitate to contact us if there are specific requests from the reviewers that you believe are technically impossible or unlikely to yield a meaningful outcome.

In your revision, we ask you to address all reviewers' concerns, to justify your parameter choices, and to perform new sensitivity analyses around the key parameters, including the number of strains and mutation rates (which currently seems to be fixed to 0.001).

Please also address reviewer concerns about the inability of your model to account for key aspects of the pandemic, such as age stratification/differential case fatality ratios across countries and the existence of waning immunity and immune escape.

Finally, your revised manuscript must comply fully with our editorial policies and formatting requirements. Failure to do so will result in your manuscript being returned to you, which will delay its consideration. To assist you in this process, I have attached a checklist that lists all of our requirements. If you have any questions about any of our policies or formatting, please don't hesitate to contact me.

If you wish to submit a suitably revised manuscript we would hope to receive it within 6 months. We understand that the COVID-19 pandemic is causing significant disruptions which may prevent you from carrying out the additional work required for resubmission of your manuscript within this timeframe. If you are unable to submit your revised manuscript within 6 months, please let us know. We will be happy to extend the submission date to enable you to complete your work on the revision.

With your revision, please:

• Include a "Response to the editors and reviewers" document detailing, point-by-point, how you addressed each editor and referee comment. If no action was taken to address a point, you must provide a compelling argument. This response will be used by the editors to evaluate your revision and sent back to the reviewers along with the revised manuscript.

• Highlight all changes made to your manuscript or provide us with a version that tracks changes.

Please use the link below to submit your revised manuscript and related files:

[REDACTED]

Note: This URL links to your confidential home page and associated information about manuscripts you may have submitted, or that you are reviewing for us. If you wish to forward this email to co-authors, please delete the link to your homepage.

Thank you for the opportunity to review your work. Please do not hesitate to contact me if you have any questions or would like to discuss the required revisions further.

Sincerely,

Arunas Radzvilavicius, PhD
Editor

Nature Human Behaviour

Reviewer expertise:

Reviewer #1: computational epidemiology, mathematical modelling

Reviewer #2: evolutionary ecology of infectious diseases

Reviewer #3: epidemiological modelling

REVIEWER COMMENTS:

Reviewer #1:
Remarks to the Author:
In their analysis Professor Zhang and colleagues use a multi-strain metapopulation model to explore SARS-CoV-2 epidemic trajectories in HICs and LMICs under different global allocation strategies for vaccines. Their claim is that inequitable distribution of vaccines provides short-term benefits to HICs and triggers important epidemics in LMICs. The latter may represent a threat also for HICs, first because of the likelihood of importing infections from LMICs, second because these epidemics may fuel the emergence of new (more transmissible and more severe) variants.

I appreciate the attempt of the authors to deal with such an important topic. Even if their claims are fully reasonable, my feeling is that they are poorly supported by modeling results. The title of the manuscript is catchy. The authors talk about "life-saving difference to all countries", a deceased compartment is included in the model, however, they do not quantify the burden of deaths under the different vaccination strategies. I have several serious concerns on both the Methods and the presentation of results which I summarize below.

Major comments
The authors initialize the model on June 15, 2021.
1. The number of infectious individuals at time 0 is assumed to be equal to the number of active cases on the day considered. It is well known that there is a certain degree of underreporting of SARS-CoV-2 infections. I expect that in LMICs this number could be much higher due to several reasons. Indeed, LMICs are generally characterized by a younger population compared to HICs, resulting in a higher proportion of cases in younger age groups, less likely to develop symptoms. The higher fraction of cases among young people coupled with less effective testing and monitoring strategies may result in a very low detection rate. Indeed, as shown in Figure 1 a-f, the fraction of infectious individuals at time 0 seems much lower in LICs than HICs. I believe that such a different time scale in the fraction of infectious at time 0 is not realistic at all.
2. The same comment applies also to the initial fraction of recovered. Initializing recovered by considering the cumulative number of reported cases up to June 15, 2021, especially for LMICs, characterized by low detection rates, could result in a significant underestimation of the fraction of immune in the population.

3. If I correctly understand, at model initialization all infectious cases are assumed to be generated by strain 1. The transmissibility of strain 1 (T_1) is computed assuming a basic reproduction number $R_0=2.79$. This value could have been reasonable for the original SARS-CoV-2 strain, circulating world-wide in 2020, but model initialization occurs on June 15, 2021. At that time, the original strain had been largely replaced by the alpha variant (characterized by ~50% higher transmissibility) and the delta variant (~50-60% more transmissible than the alpha) had started its path to become prevalent. As stated by the authors: "the most dangerous strain (Strain 5) has a 46.41% higher transmissibility than the original strain". This means that the authors are assuming that the most transmissible variant possibly appearing in the next 5 years will be characterized by a transmissibility lower than the one estimated for the alpha variant (already prevalent at the time at which the model is initialized). This assumption is far too optimistic. Please adjust the transmissibility of strain 1 at least to the value observed for the alpha variant and explore a higher range of transmissibility for variants (possibly up to R0=10-11).
4. I am not convinced by the assumption made on NPIs. I understand that the authors estimate the contact rate c_i in country i as a function of the effective reproduction number estimated for country i at June 15 and they keep this level of NPIs over the 5 years considered. First, given the poor detection of cases in LMICs I am not sure the estimates of the effective reproduction number in those countries are reliable. Second, even when assuming that they are reliable, it is known that NPIs are usually adapted by governments in the presence of re-emergence of cases and then released when the number of cases comes again under control. Assuming that the NPIs level will remain constant for the next 5 years to a value based on a picture of the effective reproduction number at a specific time point is a very strong assumption. I believe that this assumption could make the epidemic trajectories in the different countries hardly comparable. I don't know if there is a solution to this issue. One possibility could be to explore the future epidemic trajectories in the absence of NPIs.
5. Are prioritization criteria based on incidence/prevalence decided according to the value assumed at initialization or are they updated dynamically depending on the epidemic evolution within each country?
6. I was wondering which is the percentage of people immunized with single dose vaccines worldwide (e.g. Johnson&Johnson). If this percentage is low, please consider doubling the number of doses required to build full vaccinal immunity (it is not necessary to implement dynamically the administration of the doses separately).
7. Also, the assumption of an unlimited vaccination rate is quite strong. If possible, adding an upper bound to the vaccination rate based on (eventually rough) estimates of the maximum rate achieved by LMICs and HICs could certainly benefit the interpretation of results in light of the real-world context.
8. Do you eventually re-vaccinate individuals who have lost vaccine immunity? If vaccinated individuals who lose immunity become susceptible and eligible again for vaccination, I was wondering if this assumption coupled with an unlimited vaccination rate is basically equivalent (at least in countries with a big stock of vaccines) to not considering waning immunity at all. In fact, I find it surprising that in a model considering variants progressively more transmissible, with a reduced vaccine protection and short-living vaccine immunity, the end of the epidemic is achieved. I would expect zero-COVID not to occur.
9. I find that the Figures are not sufficiently clear to transmit the information needed:
Figure 2:
- Please explain better in the caption what are the insets of Figure a-f representing.
- I would suggest adding a picture summarizing the cumulative number of deaths under the different vaccination strategies shown in Figure a-f. I find surprising that no information about COVID-19 burden of deaths is reported in the manuscript.

- What are panels k-o showing? Is this the fraction of new daily infections caused by strain m divided by the world population? I find it could be more interesting to show the share (%) of new daily cases due to the different strains (possibly on the same plot). I expect the curves to sum to 100% at each time step.
Figure 3
- Panel b,c,e,f. How is this reduction/increase computed? Do the numbers on in the right legend represent net increases or percentages?

Minor comments:
a. A reference for the average case fatality ratio worldwide (0.02) should be added.
b. I do not see significant differences in results obtained using prioritization based on incidence and prevalence. This is somehow expected. I would simplify figures in the main text and place results on one of the two in the supplementary materials.
c. It is known that COVID-19 severity strongly increases with ages. This could be one of the reasons why HICs countries, characterized by older populations, have been strongly hit by the pandemic, even in the presence of advanced and efficient health care systems, while in some other LMICs COVID-19 burden appears relatively low (see e.g. Trentini et al, BMC Medicine, 2021). Please acknowledge that one of the main limitations of your approach is that your model is not stratified by age.
d. Pag.9: reference to Figure 3b in the text. Do you mean 3d?
e. Pag.9. "Either a larger δ or a larger I_thre results in a larger reduction in cumulative cases in LMICs (Fig. 3e and f), which means the larger proportion of vaccines they share, the fewer people in LMICs will be infected.". Looking at the figure, I_thre apparently play no role (or a very limited role). I would modify to: "Larger δ results in a larger reduction in cumulative cases in LMICs (Fig. 3e and f), which means the larger proportion of vaccines they share, the fewer people in LMICs will be infected.".
f. Pag 15: "We have proposed a mathematical model to investigate both the short-term and long-term impacts of vaccine equity taking account of immune escape and global transportation.". Apparently, the authors are not including immune escape (hosts recovered from either strain are immune to all other strains). Please specify.
g. Results (pag.7): "In these new waves, infections in HICs are largely due to imported cases from LMICs.". Could you clarify if this assumption is based on your model outcome? Can the model separately keep track of secondary cases generated by imported infections?

Reviewer #2:
Remarks to the Author:
This paper aims to understand, through a multi-strain metapopulaiton mathematical model, how vaccine equity for COVID-19 can impact its global epidemiology. Briefly, it shows that vaccine unequity can only provide short term benefits to the HICs and that vaccine donations is the best strategy to decrease COVID-19 burden.

I think the paper is interesting, timely, and deserves to be published when my comments would have been included.

Major comments

My main concern is about the connectivity between LMICs and HIcs which is not explictly mentionned. However, this connectivity network is far from random and change impact dramatically their conclusion. I can understand that is not addressed explicitly, but it needs to be carefully discussed.

The second concern is about the lack of references to other works on that topic. Indeed, there are several papers discussing this topic (on other pathogens) and they have to clearly cited and discussed.

Finally, my last major concern is about the initialisation of the simulation, especially regarding the number of strains. From what I've understood, it starts with 5 strains but different initial conditions can produce very different outcomes.

Minor comments:

Figure 2 is complicated to read. Moreover, axes are not consistent. A summary figure would be better

Axes labels on figure 3 need to be clearer

Frequently, parameter symbols are mentioned inside the text. It is better to avoid that since it adds confusion

Reviewer #3:
Remarks to the Author:
Overall
The study is a very important and well-written paper aiming to measure inequity in COVID-19 vaccination distribution. The authors developed a mathematical model that explicitly considers 1) the inequity in vaccine distribution and 2) the viral evolutionary dynamics and their effects on vaccine efficacy. Their key finding suggests that vaccine inequity only provides limited and short-term benefits to HICs, leading to a moderate increase in infections and deaths in LMICs.

The work is timely and is of particular interest nowadays, with the initiation of the third booster dose in several countries. I find their mathematical model clear, transparent, and elegant.
I do have two concerns: 1) it is clear that there is a wanning of both natural and vaccine immunity. It is not being considered in their model and might affect their results.
2) COVID-19 is mild in younger age groups and but may cause severe infection in the elderly. Population from HIC, in that sense, are more susceptible to a severe outcome. Given that the model is not age-structured, it is essential to have a different mortality rate in HIC and LIC. I might have missed it, but I did not see that the authors accounted for the difference in death rates in HIC and LIC. I have several more points that may help improve the paper (please see below). I, therefore, recommend accepting the paper following a major revision.

Introduction

• Citation 6. It might be more beneficial to add policy papers (i.e, advisory committees, FDA regulations etc.) that explicitly call for a vaccination with booster doses (e.g., Israel, US, UK).

• The authors stated: "Thus, making COVID-19 3 vaccines distributed equitably is not only a moral obligation for high-income countries but also in their rational self-interest." If it has been previously found, add ref. If not, it sounds like a statement or an opinion and should not appear in the intro. I think it is part of their finding, so it should not be here. In the introduction, it might be useful to say that it has been previously shown for flu https://bmcpublichealth.biomedcentral.com/articles/10.1186/s12889-021-11601-2 , which provides a motivation for their study.

• The authors stated: "With these solutions, global vaccine distribution could no longer be a 'zero-sum game' but a 'cooperative game' " This is has been previously considered in the context of game-theoretic model. You might want to consider this study https://pubsonline.informs.org/doi/abs/10.1287/mnsc.1120.1661

Methods
• The model is very clear, well written, and transparent.
• Please insert a clear table with the parameter values used in the model – at least the main parameters.
• The authors considered strategies as follows:
o "• Population size. Priority to countries with larger population sizes. • Prevalence. Priority to countries with a higher number of active cases (currently infectious cases) per capita. • Incidence rate. Priority to countries with a higher incidence rate, which is defined as the number of new cases during two weeks as a share of the total population."
o Typically, strategies are considered in the scientific literature to work of such kind – 'morbidity based' and 'mortality based' are considered. I strongly suggest adding a mortality based strategy (i.e., prioritizing in regions of higher mortality)
Results
• Figures 2 and 3 present infections. Given that COVID-19 is typically mild or asymptomatic in young age groups, it is more important to present mortalities or severe outcomes.
Discussion
Please add two limitations and try to explain if they should affect your main outcomes:
1) waning immunity following infection (i.e., moving from recovered to or at least susceptible)
2) age-structured model

I would like to wish the authors the best of luck in addressing the review.

Author Rebuttal to Initial comments

Manuscript title: Promoting equitable access to COVID-19 vaccines makes a life-saving difference to all countries

Dear Editor and Reviewers,

Thank you so much for reviewing our paper! We really appreciate all the very helpful comments and constructive suggestions! We have significantly revised the manuscript according to the review comments. All revised portions are marked in red in the revised manuscript. In the revision, we have invited Dr. Xuan Wei (Shanghai Jiao Tong University) to be a co-author due to his contribution to additional data collection and analyses.

The comments and our specific responses are detailed below.

REVIEWER 1'S COMMENTS

Remarks to the Author:

COMMENT 1-1. *In their analysis Professor Zhang and colleagues use a multi-strain metapopulation model to explore SARS-CoV-2 epidemic trajectories in HICs and LMICs under different global allocation strategies for vaccines. Their claim is that inequitable distribution of vaccines provides short-term benefits to HICs and triggers important epidemics in LMICs. The latter may represent a threat also for HICs, first because of the likelihood of importing infections from LMICs, second because these epidemics may fuel the emergence of new (more transmissible and more severe) variants.*

I appreciate the attempt of the authors to deal with such an important topic. Even if their claims are fully reasonable, my feeling is that they are poorly supported by modeling results. The title of the manuscript is catchy. The authors talk about "life-saving difference to all countries", a deceased compartment is included in the model, however, they do not quantify the burden of deaths under the different vaccination strategies. I have several serious concerns on both the Methods and the presentation of results which I summarize below.

RESPONSE 1-1. Thank you so much for the positive comments and appreciation of the importance of our paper. We have significantly revised the paper according to your comments. Please to the following responses for detailed revisions made.

COMMENT 1-2. *The number of infectious individuals at time 0 is assumed to be equal to the number of active cases on the day considered. It is well known that there is a certain degree of underreporting of SARS-CoV-2 infections. I expect that in LMICs this number could be much higher due to several reasons. Indeed, LMICs are generally characterized by a younger population compared to HICs, resulting in a higher proportion of cases in younger age groups, less likely to develop symptoms. The higher fraction of cases among young people coupled with less effective testing and monitoring strategies may result in a very low detection rate. Indeed, as shown in Figure 1 a-f, the fraction of infectious individuals at time 0 seems much lower in LICs than HICs. I believe that such a different time scale in the fraction of infectious at time 0 is not realistic at all. The same comment applies also to the initial fraction of recovered.*

Initializing recovered by considering the cumulative number of reported cases up to June 15, 2021, especially for LMICs, characterized by low detection rates, could result in a significant underestimation of the fraction of immune in the population.

RESPONSE 1-2. Thank you very much for pointing out the potential under-reporting problem. We agree with the reviewer that such an under-reporting problem exists in the number of infections, recoveries, and deaths in most countries, particularly LMICs. Therefore, we adopted the probabilistic bias analysis [1, 2] to quantify the impact of incomplete testing and imperfect tests and estimate the actual numbers of infections, recoveries, deaths, and active cases for all countries. This method is commonly used to quantify the impact of measurement bias in epidemiologic studies [2].

More specifically, we estimated the number of tested individuals by the number of tests performed in each country, obtained from the Our World in Data (https://ourworldindata.org/coronavirus-testing) by the University of Oxford and the Global Change Data Lab [3]. Then, based on existing literature, we defined the prior distributions of the following parameters for each country:

- test sensitivity,
- the test specificity,
- the ratio of the test positivity rate of untested individuals with moderate or severe symptoms to that of tested individuals,
- the ratio of the test positivity rate of untested individuals with mild or no symptoms to that of tested individuals,
- the fraction of individuals with moderate or severe symptoms among untested individuals,
- the fraction of detected recovered individuals among recovered individuals who are tested positive ($\frac{\text{\# of detected recovered individuals who were tested positive}}{\text{\# of recovered individuals who were tested positive}}$),
- the fraction of recovered individuals among false positive cases.

Next, we randomly sampled from each prior distribution for 10^5 times using Monte Carlo techniques and generated the bias-adjusted estimates of the number of infections, recoveries, deaths, and active cases based on the bias model, as described by equations (S1)-(S12). Finally, we corrected the numbers of total infections, recoveries, deaths, active cases as the median values of all estimates.

Note that the Johns Hopkins Coronavirus Resource Center has stopped reporting recovery data for 16 countries before June 15, 2021 (Thailand, Cameroon, Serbia, the US, Belgium, Sweden, Netherlands, Ireland, the United Kingdom of Great Britain and Northern Ireland, Saint Kitts and Nevis, Greece, Spain, France, Finland, Bouvet Island, Switzerland). We also manually corrected the recovery data using the data provided by Worldometers (https://www.worldometers.info/coronavirus/) before performing the probabilistic bias analysis for these countries.

We have also released raw codes of data correction in https://github.com/jianan0099/VACEquity. The TXT file named "read_me_data_corrections.txt" provides detailed instructions about how to reproduce our estimation. Details of the probabilistic bias analysis can be found in Section 1 **Details of the probabilistic bias analysis in model initialization** in the Supplementary Information.

We ran all the experiments with corrected data. Based on the correction of source recovery data and the estimation through probabilistic bias analysis, the corrected number of total cases, total recoveries, total deaths in LMICs at time 0 (June 15, 2021) are 3.40, 3.42, 4.21 times higher than the public figures. The corrected number of total cases, total recoveries, total deaths in HICs at time 0 (June 15, 2021) are 1.23, 3.09, 1.23 times higher than the public figures. The corrected cumulative fraction of cases in LMICs is

6.32%, which is 1.72 times higher than that in HICs (3.67%). The corrected fractions of active cases on June 15, 2021, are 0.36% and 0.26% in LMICs and HICs, respectively.

Despite these noticeable differences at time 0, the predicted epidemic curves in HICs and LMICs are not affected (please refer to figures in the revised manuscript). The research findings are consistent with the original manuscript.

We added the following statement in the revised manuscript to summarize the correction process:

Method Section, **Model Initialization** Part: "Due to severe under-reporting problem worldwide, especially in LMICs with a low testing rate, we adopt the probabilistic bias analysis [1,2] to correct the number of infections, recoveries, deaths, and active cases for all countries. Please refer to the Supplementary Information for a detailed description of the data correction."

We also copied below the estimated numbers and reported numbers of cumulative infections, cumulative recoveries, cumulative deaths, and active cases on June 15, 2021, for ten countries with the largest numbers of estimated cumulative infections.

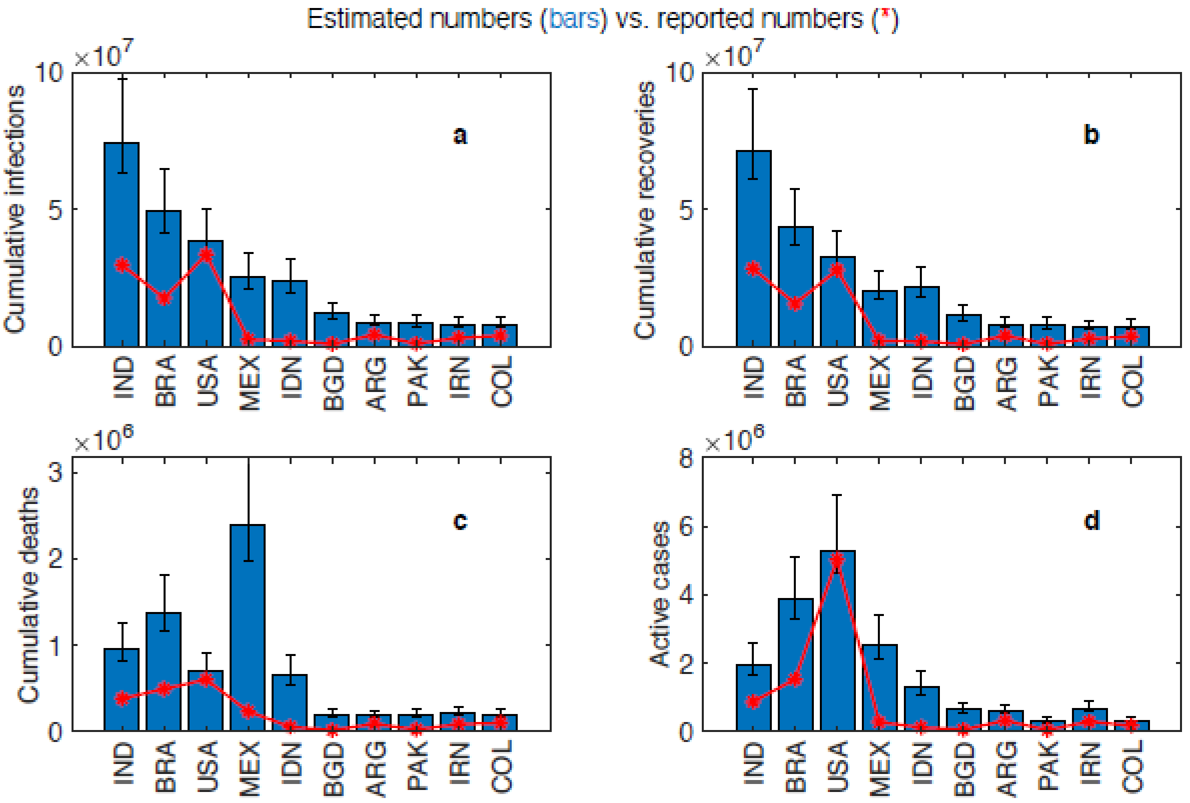

Fig. S1: Estimated numbers vs. reported numbers of (a) cumulative infections, (b) cumulative recoveries, (c) cumulative deaths, and (d) active cases on June 15, 2021, for ten countries with the largest numbers of estimated cumulative infections. Blue bars indicate the median of the sampled distribution of bias-adjusted estimates. Red asterisks (*) indicate the reported numbers. Vertical black lines indicate the 2.5th and 97.5th percentiles of the distribution of sampled distribution of bias-adjusted estimates. Only 3-letter ISO codes for countries are presented for a clear illustration. See country codes list in Table S2.

Table S2: Country Alpha 3 ISO codes list.

Country name	Alpha 3 ISO code
India	IND
Brazil	BRA
United States of America	USA
Mexico	MEX
Indonesia	IDN
Bangladesh	BGD
Argentina	ARG
Pakistan	PAK
Iran	IRN
Colombia	COL

References:

[1] Wu, S. L., Mertens, A. N., Crider, Y. S., Nguyen, A., Pokpongkiat, N. N., Djajadi, S., ... & Benjamin-Chung, J. (2020). Substantial underestimation of SARS-CoV-2 infection in the United States. Nature Communications, 11(1), 1-10.

[2] Lash, T. L., Fox, M. P., & Fink, A. K. (2009). Applying quantitative bias analysis to epidemiologic data (Vol. 192). New York: Springer. https://doi.org/10.1007/978-0-387-87959-8

[3] Hasell, J., Mathieu, E., Beltekian, D., Macdonald, B., Giattino, C., Ortiz-Ospina, E., ... & Ritchie, H. (2020). A cross-country database of COVID-19 testing. Scientific data, 7(1), 1-7.

COMMENT 1-3. *If I correctly understand, at model initialization all infectious cases are assumed to be generated by strain 1. The transmissibility of strain 1 (T_1) is computed assuming a basic reproduction number R_0=2.79. This value could have been reasonable for the original SARS-CoV-2 strain, circulating worldwide in 2020, but model initialization occurs on June 15, 2021. At that time, the original strain had been largely replaced by the alpha variant (characterized by ~50% higher transmissibility) and the delta variant (~50-60% more transmissible than the alpha) had started its path to become prevalent. As stated by the authors: "the most dangerous strain (Strain 5) has a 46.41% higher transmissibility than the original strain". This means that the authors are assuming that the most transmissible variant possibly appearing in the next 5 years will be characterized by a transmissibility lower than the one estimated for the alpha variant (already prevalent at the time at which the model is initialized). This assumption is far too optimistic. Please adjust the transmissibility of strain 1 at least to the value observed for the alpha variant and explore a higher range of transmissibility for variants (possibly up to R0=10-11).*

RESPONSE 1-3. Thank you very much for the suggestion. We agree with the reviewer that the assumption of R_0=2.79 at time 0 is too optimistic. Based on the phylogenetic analysis in the *Global Initiative for Sharing All Influenza Data* database (https://www.gisaid.org/hcov19-variants/) [1], the global relative genome frequency of the Alpha, Beta, Gamma, and Delta is 43.8%, 1.2%, 8%, and 36.1%, respectively, from June 14, 2021, to June 20, 2021. The basic reproduction number is estimated to be 4 [2,3], 4 [3], 4.4 [3], and 7 [2,3] for the Alpha, Beta, Gamma, and Delta strain, respectively. No data is available for the remaining 10.9% of the genomes. We assumed that they belong to the original strain

(R_0=2.79). Therefore, in the revised manuscript, we set R_0=2.79×10.9%+4×(43.8%+1.2%)+4.4×8%+7×36.1%≈5 at time 0.

Reviewer 2 and editors also suggested that we perform sensitivity analysis of the mutation-related parameters, M, θ, μ_1, and λ. Following all these suggestions, we used the historical data for the first 1.5 years of the pandemic (from December 31, 2019, to June 15, 2021) to inform the values of these parameters. We assumed the virus would follow a similar mutation process in the future and performed sensitivity analysis on these parameters. Results of the sensitivity analysis were similar to those reported in the main manuscript. Please refer to the description below and Fig. S6-Fig. S14 and Fig. S20-Fig. S24 in the Supplementary Information for details.

The number of "Variants of Concern" (M) emerged in the next five years: Most of the viral mutations have little impact on the virus' ability to transmit and cause severe infections. Variants meeting specific criteria (e.g., increase in transmissibility, increase in virulence, decrease in the effectiveness of public health measures.) are designated as "Variants of Concern" by the World Health Organization [4]. Although there are thousands of genetic variants of SARS-CoV-2 [5], only four of them are designated as "Variants of Concern" as of June 15, 2021, i.e., the Alpha, Beta, Gamma, and Delta strain. Thus, the value of M for the first 1.5 years of the pandemic is 5. In the revised manuscript, we assumed M ranges from 3 to 10 in the next five years.

The increase in the transmissibility of each new strain (θ): The most transmissible strain, Delta, demonstrates 2.5 times higher transmissibility than the original strain. Therefore, here we assume a linear strain space and local movement by a one-direction stepwise mutation [6], then the transmissibility of each new strain is 26% (1.26^4≈2.5) higher than the immediate previous strain, i.e., θ=0.26 for the first 1.5 years of the pandemic. In the revised manuscript, we assumed θ ranges from 0.15 to 0.5 in the next five years.

The mutation probability per infection of the strain at time 0 (μ_1), and decrease rate of the mutation probability per infection (λ): There is evidence that mutations in the genome region encoding the spike protein (3822 nucleotides in length from site 21563 to 25384 [7]) may result in increased transmissibility [8] and viral load [9] of the virus. It is estimated that the mutation rate per virus replication cycle per site is 3×10^{-6}, and the entire course of infection will take approximately five viral replication cycles [10]. Thus, the probability that the spike protein region changes per infection is $1-((1-3\times10^{-6})^5)^{3822}\approx0.056$. Most of such mutations are neutral. Moreover, the virus cannot evolve indefinitely, primarily because each nucleotide can only mutate to three others (e.g., adenine (A) can only mutate to thymine (T) or guanine (G) or cytosine (C)), and we have limited number of nucleotides [11]. As the virus evolves in the strain space, the probability of major and new changes per infection decreases because fewer possible genome sequences remain. It means that the probability of emerging new and more dangerous strains per infection decreases over time [12]. Based on these facts, we assume that (a) for strain 1, only 1‱~10% of such mutations can significantly affect the virus' ability to cause infections, i.e., μ_1 ranges from 5.6×10^{-6} to 5.6×10^{-3} in the next five years; (b) $\mu_{m+1} = \mu_m/\lambda$, where λ quantifies the decrease rate of the probability of emerging new and more dangerous strains per infection. λ ranges from 10^2 to 10^4 in the next five years.

The revised parts are copied below:

Method Section, **Multi-strain model** Part: "The virus in strain m can either remains as strain m with probability $1-\mu_m$ or mutates to strain $m+1$ (one-direction stepwise mutation) with probability μ_m

while adapting to a new host (please refer to the Supplementary Information for details of the spreading process), thus, we construct $\mathcal{U}$ as

$$\mathcal{U} = \begin{bmatrix} 1-\mu_1 & \mu_1 & \cdots & 0 & 0 \\ 0 & 1-\mu_2 & \mu_2 & \cdots & 0 \\ \vdots & \vdots & \vdots & \ddots & \vdots \\ 0 & 0 & 0 & \cdots & 1 \end{bmatrix}.$$

The virus cannot evolve indefinitely, primarily because each nucleotide can only mutate to three others (e.g., adenine can only mutate to thymine or guanine or cytosine), and the genome of SARS-CoV-2 has limited nucleotides in length [10, 11]. As the virus evolves in the strain space, the probability of major and new changes per infection decreases because fewer possible genome sequences remain. Thus, we assume $\mu_{m+1} = \mu_m/\lambda$, where λ decrease rate of the probability of emerging new and more dangerous strains…specifically, we assume $\mathcal{T}_{n+1} = (1+\theta)\mathcal{T}_n$."

Section 4 **Model parameter settings** in the Supplementary Information:"$\mathcal{T}_1$ is estimated by the initial basic reproduction number R_0 divided by the infectious period (5 days). Based on the phylogenetic analysis in the Global Initiative for Sharing All Influenza Data [1], the global relative genome frequency of the Alpha, Beta, Gamma, and Delta is 43.8%, 1.2%, 8%, and 36.1%, respectively, from June 14, 2021, to June 20, 2021. The basic reproduction number is estimated to be 4 [2,3], 4 [3], 4.4 [3], and 7 [2,3] for the Alpha, Beta, Gamma, and Delta strain, respectively. No data is available for the remaining 10.9% of the genomes. We assume that they belong to the original strain (R_0=2.79). Therefore, we set R_0=2.79×10.9%+4×(43.8%+1.2%)+4.4×8%+7×36.1%≈5 at time 0… Because of limited data to quantify the viral mutation parameters, M, θ, μ_1, and λ, we use the historical data for the first 1.5 years of the pandemic (from December 31, 2019, to June 15, 2021) to inform the values of these parameters. We assume the virus follows a similar mutation process in the future and perform sensitivity analysis on these parameters (Fig. S6-S14, Fig. S20-S24)."

References:

[1] Shu, Y., & McCauley, J. (2017). GISAID: Global initiative on sharing all influenza data–from vision to reality. Eurosurveillance, 22(13), 30494.

[2] Burki, T. K. (2021). Lifting of COVID-19 restrictions in the UK and the Delta variant. The Lancet Respiratory Medicine, 9(8), e85.

[3] Campbell, F., Archer, B., Laurenson-Schafer, H., Jinnai, Y., Konings, F., Batra, N., ... & de Waroux, O. L. P. (2021). Increased transmissibility and global spread of SARS-CoV-2 variants of concern as at June 2021. Eurosurveillance, 26(24), 2100509.

[4] WHO. Tracking SARS-CoV-2 variants (2021). https://www.who.int/en/activities/tracking-SARS-CoV-2-variants/

[5] Rambaut, A., Holmes, E. C., O'Toole, Á., Hill, V., McCrone, J. T., Ruis, C., ... & Pybus, O. G. (2020). A dynamic nomenclature proposal for SARS-CoV-2 lineages to assist genomic epidemiology. Nature microbiology, 5(11), 1403-1407.

[6] Gog, J. R., & Grenfell, B. T. (2002). Dynamics and selection of many-strain pathogens. Proceedings of the National Academy of Sciences, 99(26), 17209-17214.

[7] Yang, H. C., Chen, C. H., Wang, J. H., Liao, H. C., Yang, C. T., Chen, C. W., ... & Liao, J. C. (2020). Analysis of genomic distributions of SARS-CoV-2 reveals a dominant strain type with strong allelic associations. Proceedings of the National Academy of Sciences, 117(48), 30679-30686.

[8] Li, Q., Wu, J., Nie, J., Zhang, L., Hao, H., Liu, S., ... & Wang, Y. (2020). The impact of mutations in SARS-CoV-2 spike on viral infectivity and antigenicity. Cell, 182(5), 1284-1294.

[9] Frampton, D., Rampling, T., Cross, A., Bailey, H., Heaney, J., Byott, M., ... & Nastouli, E. (2021). Genomic characteristics and clinical effect of the emergent SARS-CoV-2 B. 1.1. 7 lineage in London, UK: a whole-genome sequencing and hospital-based cohort study. The Lancet Infectious Diseases, 21(9), 1246-1256.

[10] Sender, R., Bar-On, Y. M., Gleizer, S., Bernshtein, B., Flamholz, A., Phillips, R., & Milo, R. (2021). The total number and mass of SARS-CoV-2 virions. Proceedings of the National Academy of Sciences, 118(25).

[11] Burioni, R., & Topol, E. J. (2021). Has SARS-CoV-2 reached peak fitness?. Nature Medicine, 27 (8), 1323–1324.

[12] Kupferschmidt, K. (2021). Evolving threat. Science, 373(6557) 844-849. https://doi.org/10.1126/science.373.6557.844.

COMMENT 1-4. *I am not convinced by the assumption made on NPIs. I understand that the authors estimate the contact rate c_i in country i as a function of the effective reproduction number estimated for country i at June 15 and they keep this level of NPIs over the 5 years considered. First, given the poor detection of cases in LMICs I am not sure the estimates of the effective reproduction number in those countries are reliable. Second, even when assuming that they are reliable, it is known that NPIs are usually adapted by governments in the presence of re-emergence of cases and then released when the number of cases comes again under control. Assuming that the NPIs level will remain constant for the next 5 years to a value based on a picture of the effective reproduction number at a specific time point is a very strong assumption. I believe that this assumption could make the epidemic trajectories in the different countries hardly comparable. I don't know if there is a solution to this issue. One possibility could be to explore the future epidemic trajectories in the absence of NPIs.*

RESPONSE 1-4. Thank you very much for the valuable comment and suggestion. We agree with the reviewer that the assumption that the level of NPIs will remain constant for the next five years based on a picture of the effective reproduction number at a specific time point is not fully realistic. To provide a more convincing description of how countries introduce and relax NPIs during the COVID-19 pandemic, we consider the reproduction number-based adaptive policy adoption strategy [1-3], where more stringent NPIs are triggered when the local effective reproduction number exceeds a certain threshold, and are relaxed to less stringent when it falls below the threshold. Specifically, following [3], we integrated the impact of NPIs through a reduction in the value of the basic reproduction number and considered two levels of NPI intensity: stringent and mild NPIs. Denote the effectiveness of stringent and mild NPIs as $c_{stringent}$ and c_{mild}, respectively. Then, the effectiveness of NPIs at time t for country i is represented by

$$c_i(t) = \begin{cases} c_{stringent} & R_{e,i}(t) \geq 1, \\ c_{mild} & R_{e,i}(t) < 1. \end{cases}$$

$R_{e,i}(t)$ is the local effective reproduction number for country i at time t, which can be computed as the dominant eigenvalue of the Next Generation Matrix (NGM) [4] associated with the dynamical system considered. The NGM for country i is given by

$$NGF_i = M_{F,i} M_{V,i}{}^{-1} = \begin{bmatrix} 0 & 0 & (1-c_i)S_i/N_i(\mathcal{TU})^T & (1-c_i)S_i/N_i(\mathcal{TU})^T \\ 0 & 0 & (1-c_i)V_i/N_i(M_\eta \mathcal{TU})^T & (1-c_i)V_i/N_i(M_\eta \mathcal{TU})^T \\ 0 & 0 & 0 & 0 \\ 0 & 0 & 0 & 0 \end{bmatrix} \begin{bmatrix} \frac{1}{\sigma}1 & 0 \\ \frac{1}{\alpha}1 & \frac{1}{\alpha}1 \end{bmatrix}.$$

Here, $M_{F,i}$ and $M_{V,i}$ are the transmission matrix and the transition matrix for country i, respectively. Details of the derivation of $R_{e,i}(t)$ could be found in Section 2 **Details of the adaptive policy adoption strategy** in the Supplementary Information. According to the assessment of different control measures based on the real-world data [5, 6], we set the effectiveness of stringent and mild NPIs as 80% and 40%, respectively. The threshold of $R_{e,i}(t)$ triggering stringent NPIs is set as 1, and results are reported in the main manuscript. We also performed the sensitivity analysis of the threshold and reported the results in the Supplementary Information.

We added the following statement in the main manuscript:

Method Section, **Deterministic, discrete-time SVEIRD based metapopulation model** Part:

"To model how countries introduce and relax NPIs during the COVID-19 pandemic, we consider the reproduction number-based adaptive policy adoption strategy [1-3], where more stringent NPIs are triggered when the local effective reproduction number exceeds a certain threshold, and are relaxed to less stringent when it falls below the threshold. Note that each country introduces or relaxes NPIs based on the local effective reproduction number within each country, instead of the effective reproduction number for the metapopulation network [4, 7, 8]. Details of the adaptive policy adoption strategy can be found in the Supplementary Information."

Section 2 **Details of the adaptive policy adoption strategy** in the Supplementary Information:

"In the main text, we assume that the threshold of the local effective reproduction number leading to stringent NPIs is 1. Sensitivity analysis results (Fig. S28-S31) show that, relaxation of NPIs before the pandemic is well-contained substantially extends the duration of the pandemic and leads to more deaths globally. With a higher threshold of the local effective reproduction number leading to stringent NPIs, HICs need to donate more vaccines to protect themselves."

The added figures are copied below:

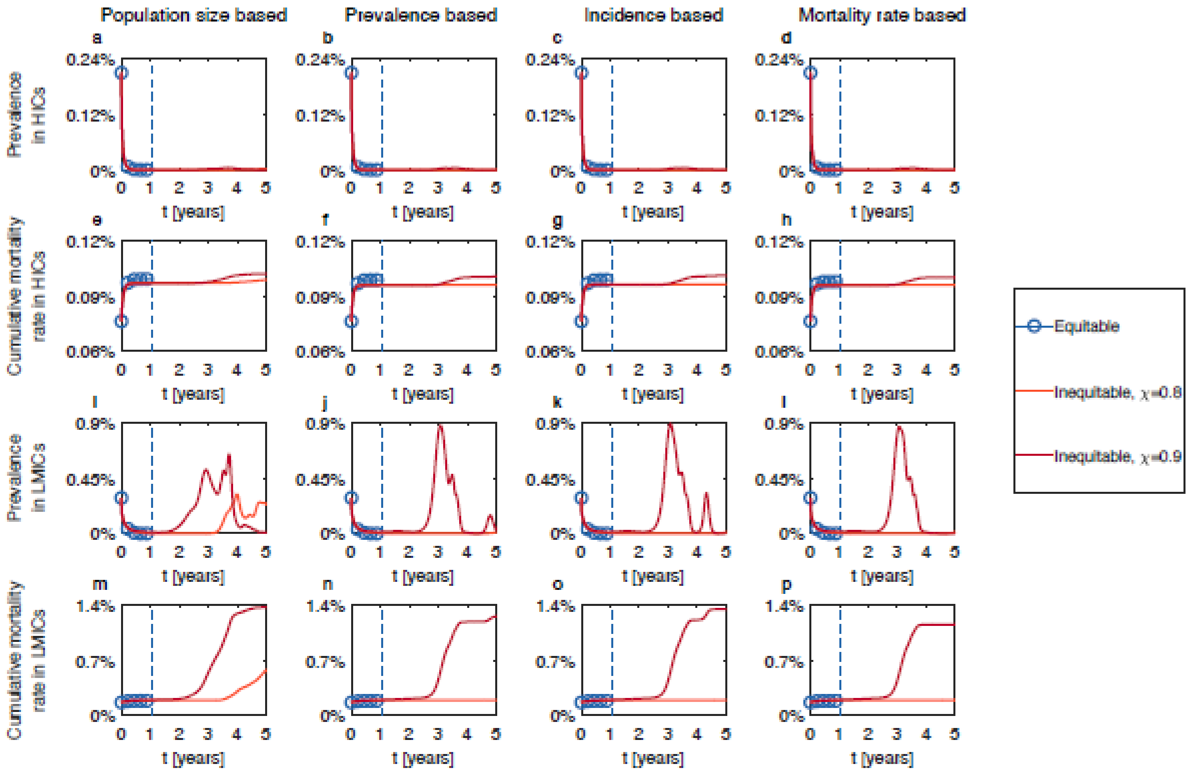

Figure S28: a-h, Time series of the prevalence (a-d) and the cumulative mortality rate (e-h) in HICs under different global vaccine allocation strategies. i-p, Time series of the prevalence (i-l) and the cumulative mortality rate (m-p) in LMICs under different global vaccine allocation strategies. Four prioritization criteria for allocation are adopted: the population size (the left panel), prevalence (second left panel), incidence (second right panel), and mortality rate (the right panel). Dash lines indicate the time when the pandemic ends (time exceeding five years is not presented). Stringent NPIs are triggered when the local effective reproduction number exceeds 0.8. Parameter values M=5, μ_1=5.6e-3, θ=0.2, and λ=500.

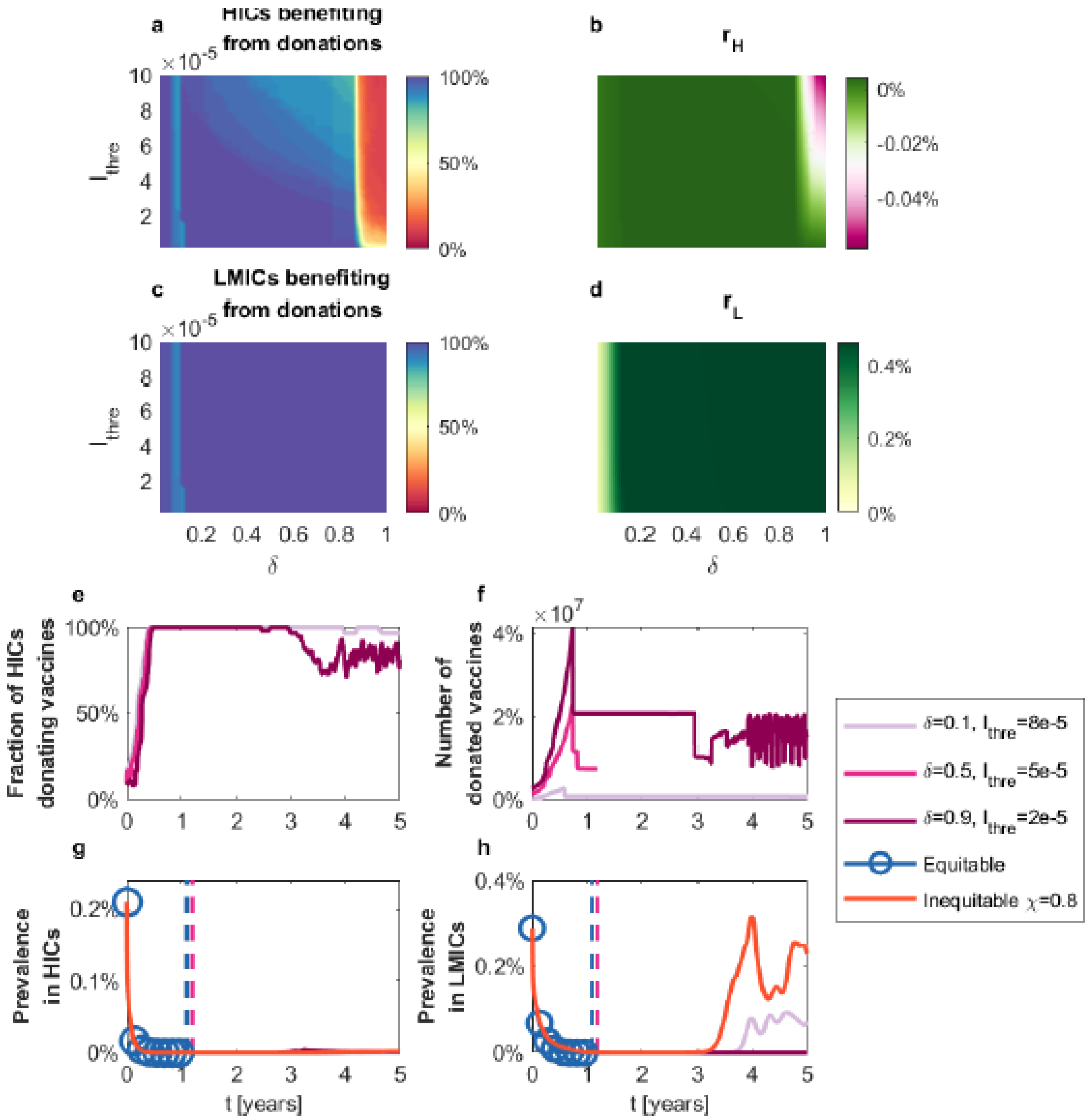

Figure S29: Impacts of different allow-donation vaccine allocation strategies on epidemic dynamics. a and c, Fraction of HICs and LMICs benefiting from donations. b and d, Average lives saved by vaccine donations as the share of the national population in HICs (r_H) and LMICs (r_L). e, Fraction of HICs donating vaccines. f, Total number of donated vaccines. g and h, Prevalence in HICs and LMICs under different vaccine allocation strategies. Countries with larger population sizes are prioritized for vaccination. Stringent NPIs are triggered when the local effective reproduction number exceeds 0.8. Parameter values M=5, μ_1=5.6e-3, θ=0.2, and λ=500.

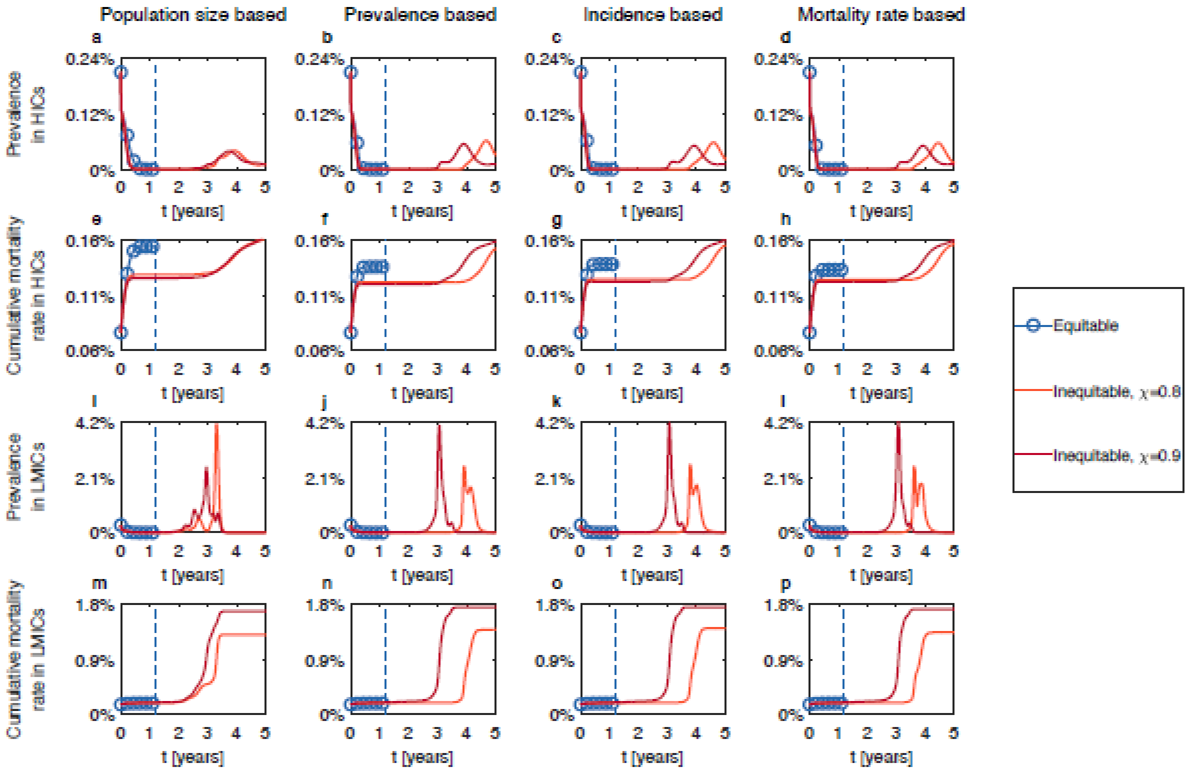

Figure S30: a-h, Time series of the prevalence (a-d) and the cumulative mortality rate (e-h) in HICs under different global vaccine allocation strategies. i-p, Time series of the prevalence (i-l) and the cumulative mortality rate (m-p) in LMICs under different global vaccine allocation strategies. Four prioritization criteria for allocation are adopted: the population size (the left panel), prevalence (second left panel), incidence (second right panel), and mortality rate (the right panel). Dash lines indicate the time when the pandemic ends (time exceeding five years is not presented). Stringent NPIs are triggered when the local effective reproduction number exceeds 1.2. Parameter values M=5, μ_1=5.6e-3, θ=0.2, and λ=500.

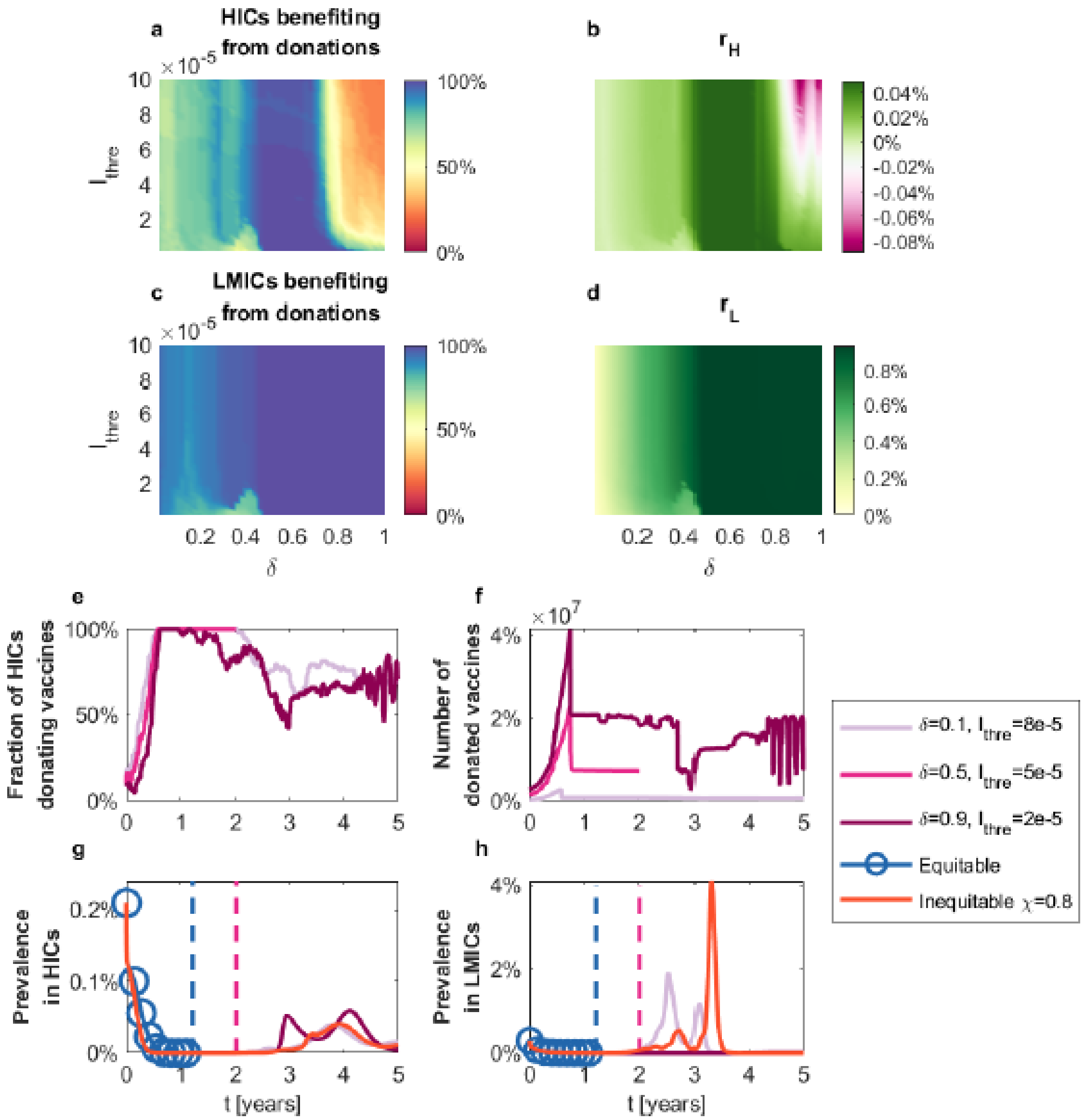

Figure S31: Impacts of different allow-donation vaccine allocation strategies on epidemic dynamics. a and c, Fraction of HICs and LMICs benefiting from donations. b and d, Average lives saved by vaccine donations as the share of the national population in HICs (r_H) and LMICs (r_L). e, Fraction of HICs donating vaccines. f, Total number of donated vaccines. g and h, Prevalence in HICs and LMICs under different vaccine allocation strategies. Countries with larger population sizes are prioritized for vaccination. Stringent NPIs are triggered when the local effective reproduction number exceeds 1.2. Parameter values M=5, μ_1=5.6e-3, θ=0.2, and λ=500.

References:

[1] Ferguson, N., Laydon, D., Nedjati Gilani, G., Imai, N., Ainslie, K., Baguelin, M., ... & Ghani, A. (2020). Report 9: Impact of non-pharmaceutical interventions (NPIs) to reduce COVID19 mortality and healthcare demand.

[2] Jiannan Yang, Qingpeng Zhang, Zhidong Cao, Jianxi Gao, Dirk Pfeiffer, Lu Zhong, and Daniel Dajun Zeng, "The impact of non-pharmaceutical interventions on the prevention and control of COVID-19 in New York City", Chaos 31, 021101 (2021) https://doi.org/10.1063/5.0040560

[3] Yang, J., Marziano, V., Deng, X., Guzzetta, G., Zhang, J., Trentini, F., ... & Yu, H. (2021). Despite vaccination, China needs non-pharmaceutical interventions to prevent widespread outbreaks of COVID-19 in 2021. Nature Human Behaviour, 1-12.

[4] Diekmann, O., Heesterbeek, J. A. P., & Metz, J. A. (1990). On the definition and the computation of the basic reproduction ratio R0 in models for infectious diseases in heterogeneous populations. Journal of mathematical biology, 28(4), 365-382.

[5] Flaxman, S., Mishra, S., Gandy, A., Unwin, H. J. T., Mellan, T. A., Coupland, H., ... & Bhatt, S. (2020). Estimating the effects of non-pharmaceutical interventions on COVID-19 in Europe. Nature, 584(7820), 257-261.

[6] Saad-Roy, C. M., Wagner, C. E., Baker, R. E., Morris, S. E., Farrar, J., Graham, A. L., ... & Grenfell, B. T. (2020). Immune life history, vaccination, and the dynamics of SARS-CoV-2 over the next 5 years. Science, 370(6518), 811-818.

[7] Anzo-Hernández, A., Bonilla-Capilla, B., Velázquez-Castro, J., Soto-Bajo, M., & Fraguela-Collar, A. (2019). The risk matrix of vector-borne diseases in metapopulation networks and its relation with local and global R0. Communications in Nonlinear Science and Numerical Simulation, 68, 1-14.

[8] Cao, L., Li, X., Wang, B., & Aihara, K. (2011). Rendezvous effects in the diffusion process on bipartite metapopulation networks. Physical Review E, 84(4), 041936.

COMMENT 1-5. *Are prioritization criteria based on incidence/prevalence decided according to the value assumed at initialization or are they updated dynamically depending on the epidemic evolution within each country?*

RESPONSE 1-5. Thank you for the comment. All prioritization criteria are being updated dynamically based on the epidemic evolution within each country. A detailed description of this dynamic vaccine distribution process is presented in Section 6 **Details of global vaccine allocation strategies** in the Supplementary Information. We added the following statement in the revised manuscript to avoid confusion:

Method Section, **Global vaccine allocation model** Part: "All prioritization criteria are being updated dynamically based on the epidemic evolution within each country."

COMMENT 1-6. *I was wondering which is the percentage of people immunized with single dose vaccines worldwide (e.g. Johnson&Johnson). If this percentage is low, please consider doubling the number of doses required to build full vaccinal immunity (it is not necessary to implement dynamically the administration of the doses separately).*

RESPONSE 1-6. Thank you for the valuable comment. As of March 9, 2021, only 4% are single-dose vaccines among all ordered vaccines produced by the top 10 manufacturers [1]. As recommended by the reviewer, we doubled the number of doses required to build full vaccinal immunity and assumed that two doses are administered simultaneously for simplicity. We copied the modifications in the revised manuscript below:

In the main text:

Method Section, **Global vaccine allocation model** Part:

"As of March 9, 2021, single-dose vaccines account for only 4% among all ordered vaccines produced by the top 10 manufacturers [1]. For simplicity, we assume that (1) all vaccines are administered with a two-dose schedule; (2) two doses are administered simultaneously; (3) the body can build full vaccinal immunity immediately after vaccination; (4) the upper bounds of daily vaccination rates for HICs and LMICs are the maximum daily vaccination rates achieved by HICs and LMICs from January 1, 2020, to June 15, 2021 (*t*=0)."

In the Supplementary Information:

Section 6 **Details of global vaccine allocation strategies**: "Denote $vs_i(t)$ as the vaccine stock held by country i at time t, then the demand of vaccines for country i at time t is $dem_i(t) = \max\{2S_i(t) - vs_i(t), 0\}$, which means each country orders vaccines that can vaccinate the entire susceptible population."

"The number of individuals that can be fully vaccinated for country i at time t equals half the number of available vaccines for country i at time t, i.e.,

$$\frac{\Omega_i(t) + vs_i(t)}{2}$$

Denote the maximum daily vaccination rate for country i as $\overline{\phi}_i$. Then, the vaccination rate for country i at time t should not exceed either the available vaccine supply or the maximum daily vaccination rate, i.e.,

$$\phi_i(t) = \min\left\{S_i(t), \frac{\Omega_i(t) + vs_i(t)}{2}, \overline{\phi}_i\right\}.$$

The vaccine stock held by country i at next time is

$$vs_i(t+1) = \Omega_i(t) + vs_i(t) - 2\phi_i(t).$$

In all simulations, we set $\varphi(\tau) = \sum_i N_i$, and τ is 183 days. If the upper bounds of daily vaccination rates for HICs and LMICs are the maximum daily vaccination rates achieved by HICs and LMICs by June 15, 2021.

$$\overline{\phi}_i = \begin{cases} N_i max_{j\in H}\dfrac{\tilde{\phi}_j}{2N_j} & i\in H, \\ N_i max_{j\in L}\dfrac{\tilde{\phi}_j}{2N_j} & i\in L, \end{cases}$$

where $\tilde{\phi}_i$ is the maximum daily vaccine doses administrated by country i from January 1, 2020, to June 15, 2021 (t=0). After excluding the extremely high daily vaccination rates in Bhutan (with a maximum daily vaccination rate of 6% of the population), the upper bounds of the daily vaccination rate for HICs and LMICs are 1.2% and 1.9%, respectively.

References:

[1] Bloomberg. Covid-19 Deals Tracker: 9.6 Billion Doses Under Contract (2021).

https://www.bloomberg.com/graphics/covid-vaccine-tracker-global-distribution/contracts-purchasing-agreements.html

COMMENT 1-7. *Also, the assumption of an unlimited vaccination rate is quite strong. If possible, adding an upper bound to the vaccination rate based on (eventually rough) estimates of the maximum rate achieved by LMICs and HICs could certainly benefit the interpretation of results in light of the real-world context.*

RESPONSE 1-7. Thank you for the valuable comment. Following the suggestion, we set the upper bound of vaccination rates for HICs and LMICs as the maximum daily vaccination rate achieved by HICs (1.2% of the population per day in Seychelles on February 13, 2021) and LMICs (1.9% of the population per day in Mongolia on May 7, 2021) by June 15, 2021, respectively. Note that the upper bound in HICs is smaller because of the vaccine hesitancy [1]. We added this restriction in the main text and the details in the Supplementary Information (please refer to **RESPONSE 1-6**).

References:

[1] Machingaidze, S., & Wiysonge, C. S. (2021). Understanding COVID-19 vaccine hesitancy. Nature Medicine, 27(8), 1338-1339.

COMMENT 1-8. *Do you eventually re-vaccinate individuals who have lost vaccine immunity? If vaccinated individuals who lose immunity become susceptible and eligible again for vaccination, I was wondering if this assumption coupled with an unlimited vaccination rate is basically equivalent (at least in countries with a big stock of vaccines) to not considering waning immunity at all. In fact, I find it surprising that in a model considering variants progressively more transmissible, with a reduced vaccine protection and short-living vaccine immunity, the end of the epidemic is achieved. I would expect zero-COVID not to occur.*

RESPONSE 1-8. Thank you very much for the comment. Yes, we assume that people get re-vaccinated in the model. The figure below shows half the cumulative doses administrated globally over time under the inequitable vaccine allocation strategy with χ=0.8. We take the half because we assume an individual takes two shots to finish one round of vaccination. As the figure indicates, the total dosage exceeds the world population.

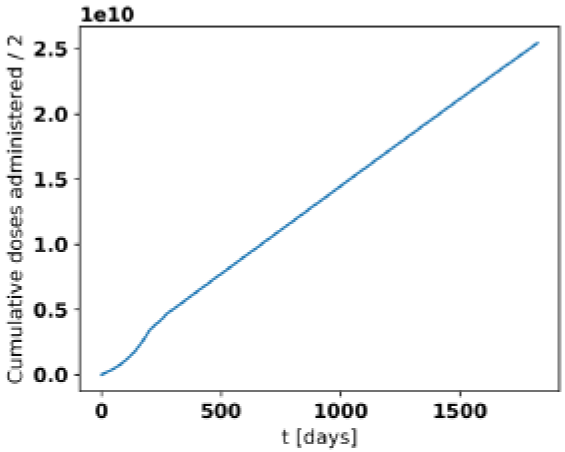

Specifically, the cumulative doses administrated at the end of the fifth year can vaccinate the world population more than 3.5 times over, which means people get re-vaccinated. Although the vaccine protection will be reduced due to evolving variants, the reduction is not as significant as losing vaccine immunity at all [1]. Therefore, the end of the epidemic can be achieved in certain scenarios. Specifically, under inequitable vaccine allocation strategies, the epidemic will not end unless the virus mutates slowly and newly emerged strains are not highly transmissible (e.g., M=5, μ_1=5.6e-4, θ=0.1, and λ=1000 in Figure S10). Under equitable vaccine allocation strategies, the epidemic will end in most cases. However, if the virus becomes highly transmissible in the short term and NPIs are relaxed before the epidemic is well-contained, the end of the epidemic cannot be achieved even under equitable vaccine allocation strategies (e.g., M=3, μ_1=5.6e-3, θ=0.5, and λ=500 in Figure S32).

The added figures are copied below:

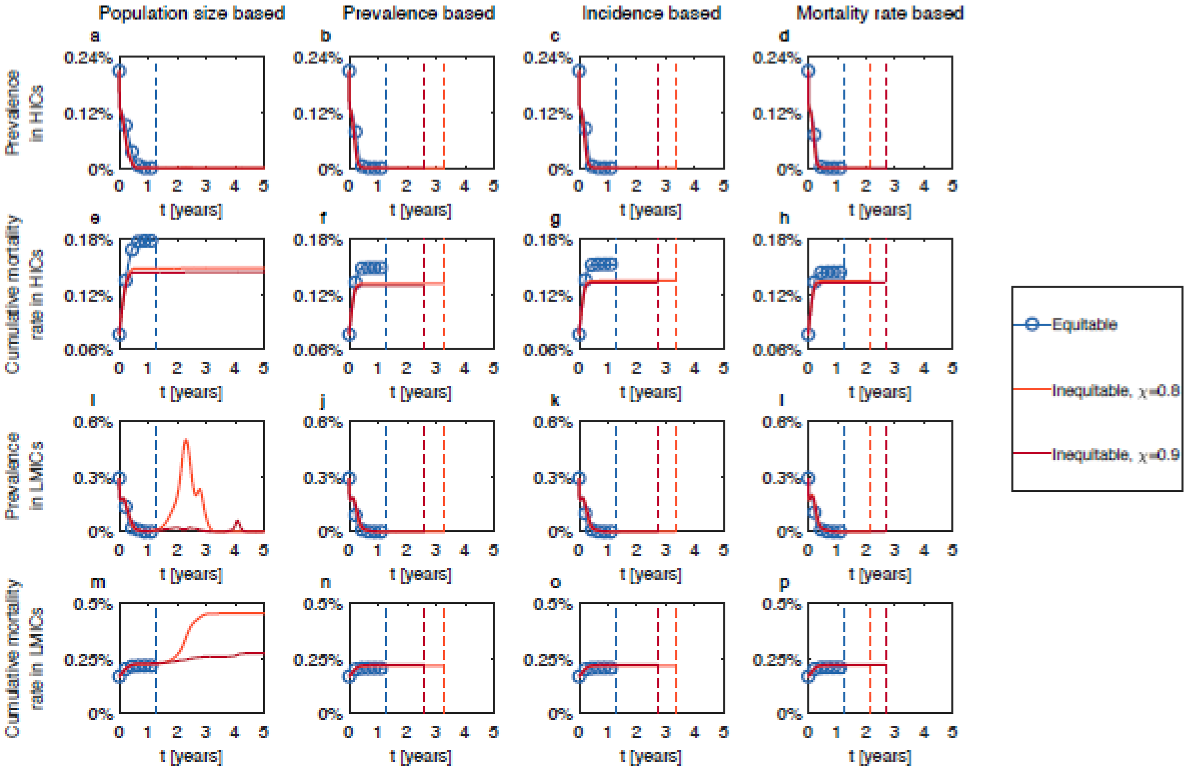

Figure S10: a-h, Time series of the prevalence (a-d) and the cumulative mortality rate (e-h) in HICs under different global vaccine allocation strategies. i-p, Time series of the prevalence (i-l) and the cumulative mortality rate (m-p) in LMICs under different global vaccine allocation strategies. Four prioritization criteria for allocation are adopted: the population size (the left panel), prevalence (second left panel), incidence (second right panel), and mortality rate (the right panel). Dash lines indicate the time when the pandemic ends (time exceeding five years is not presented). Stringent NPIs are triggered when the local effective reproduction number exceeds 1. Parameter values M=5, μ_1=5.6e-4, θ=0.1, and λ=1000.

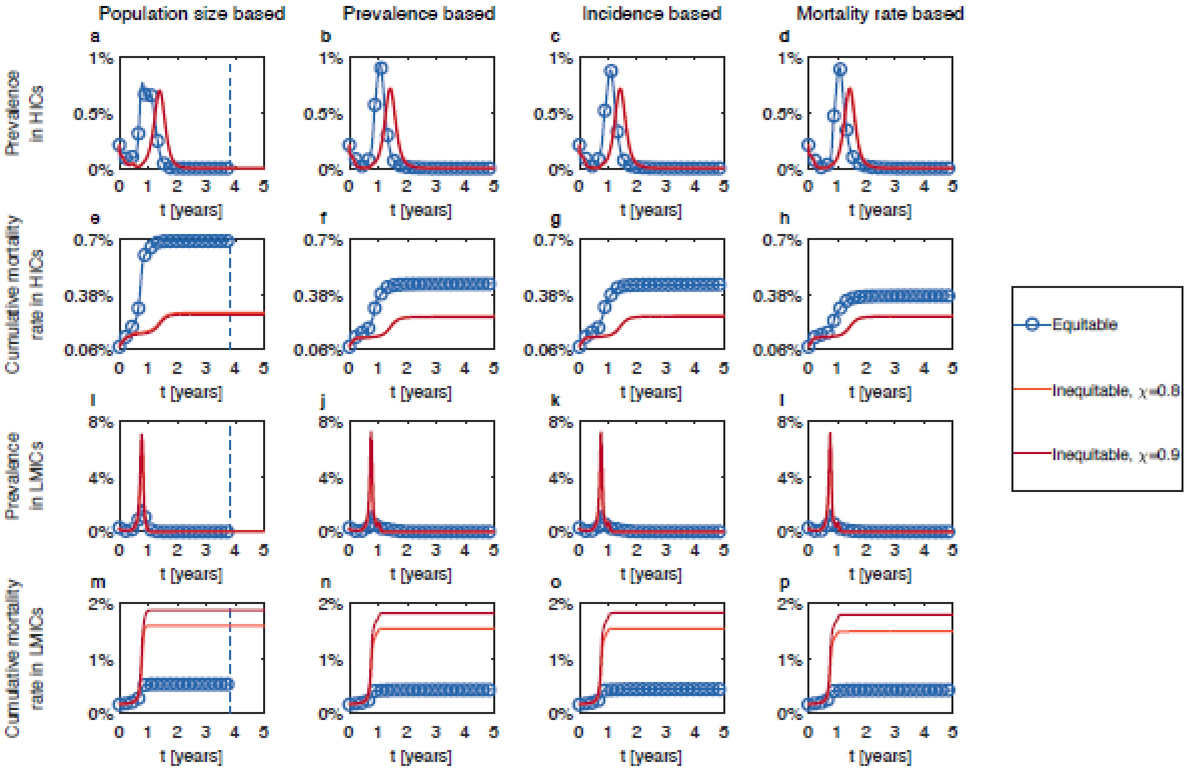

Figure S32: a-h, Time series of the prevalence (a-d) and the cumulative mortality rate (e-h) in HICs under different global vaccine allocation strategies. i-p, Time series of the prevalence (i-l) and the cumulative mortality rate (m-p) in LMICs under different global vaccine allocation strategies. Four prioritization criteria for allocation are adopted: the population size (the left panel), prevalence (second left panel), incidence (second right panel), and mortality rate (the right panel). Dash lines indicate the time when the pandemic ends (time exceeding five years is not presented). Stringent NPIs are triggered when the local effective reproduction number exceeds 1.4. Parameter values M=3, μ_1=5.6e-3, θ=0.5, and λ=500.

References:

[1] Lopez Bernal, J., Andrews, N., Gower, C., Gallagher, E., Simmons, R., Thelwall, S., ... & Ramsay, M. (2021). Effectiveness of Covid-19 vaccines against the B. 1.617. 2 (Delta) variant. N Engl J Med, 585-594.

COMMENT 1-9. *I find that the Figures are not sufficiently clear to transmit the information needed:*

Figure 2:

a. Please explain better in the caption what are the insets of Figure a-f representing.

RESPONSE 1-9a. Thank you for the suggestion. For clearer visualization, we split Figure 2 into four figures (Figure 2 and Figure 3 in the main text; Figure S2 and Figure S4 in the Supplementary Information) in the revised manuscript for better understanding. In the initial submission, the insets of Figure 2a-2f are the zoomed version of Figure 2a-2f, which shows only the fraction of infectious

individuals from the second year. In the revised manuscript, these results are presented separately in Figure S4 in the Supplementary Information. Figure S4 is copied below:

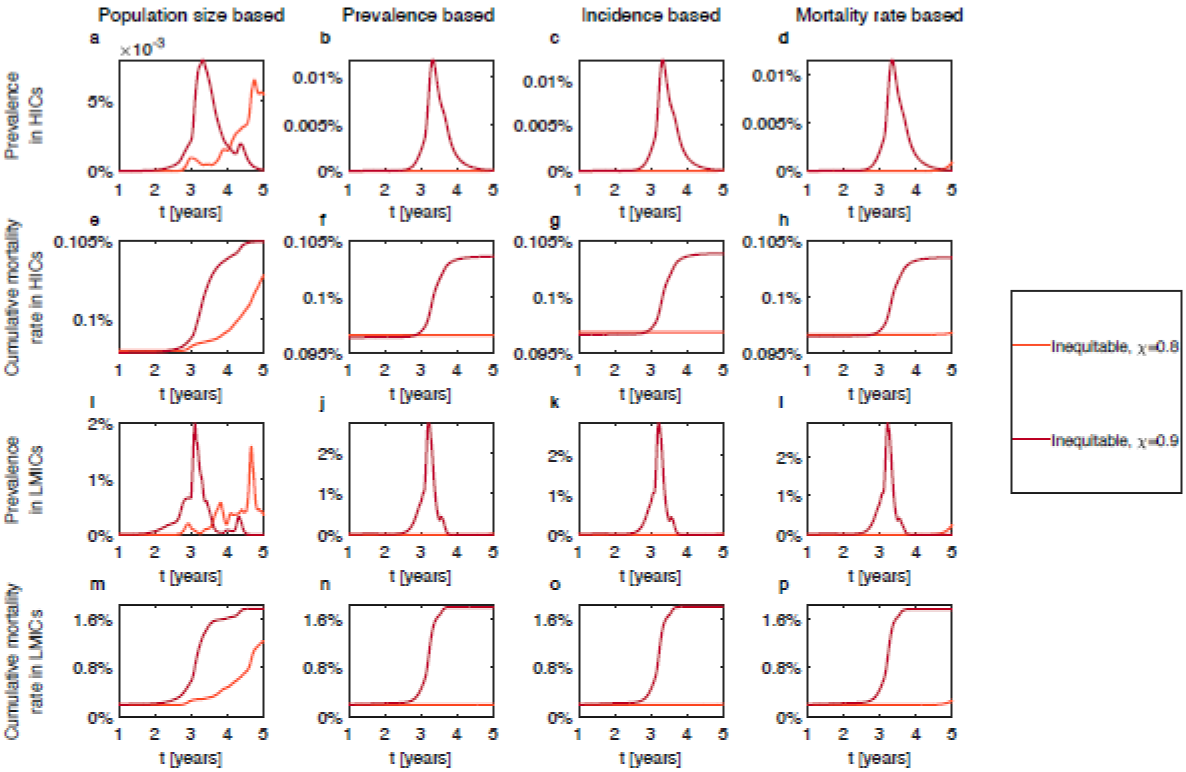

Figure S4: a-h, Time series of the prevalence (a-d) and the cumulative mortality rate (e-h) in HICs under different global vaccine allocation strategies. i-p, Time series of the prevalence (i-l) and the cumulative mortality rate (m-p) in LMICs under different global vaccine allocation strategies. Four prioritization criteria for allocation are adopted: the population size (the left panel), prevalence (second left panel), incidence (second right panel), and mortality rate (the right panel). Results from the second year are presented. Parameter values M=5, μ_1=5.6e-3, θ=0.2, and λ=500.

b. *I would suggest adding a picture summarizing the cumulative number of deaths under the different vaccination strategies shown in Figure a-f. I find surprising that no information about COVID-19 burden of deaths is reported in the manuscript.*

RESPONSE 1-9b. Thank you for the valuable suggestion. In the revised manuscript, we present the prevalence and the cumulative mortality rate in HICs and LMICs under different global vaccine allocation strategies in Figure 2. To provide more information about the COVID-19 burden of deaths, we also replace results about cumulative cases in Figure 3 and Figure 4 (in the initial submission) with the cumulative mortality rate (Figure 5 in the revised manuscript). The revised Figure 2 in the main text is copied below:

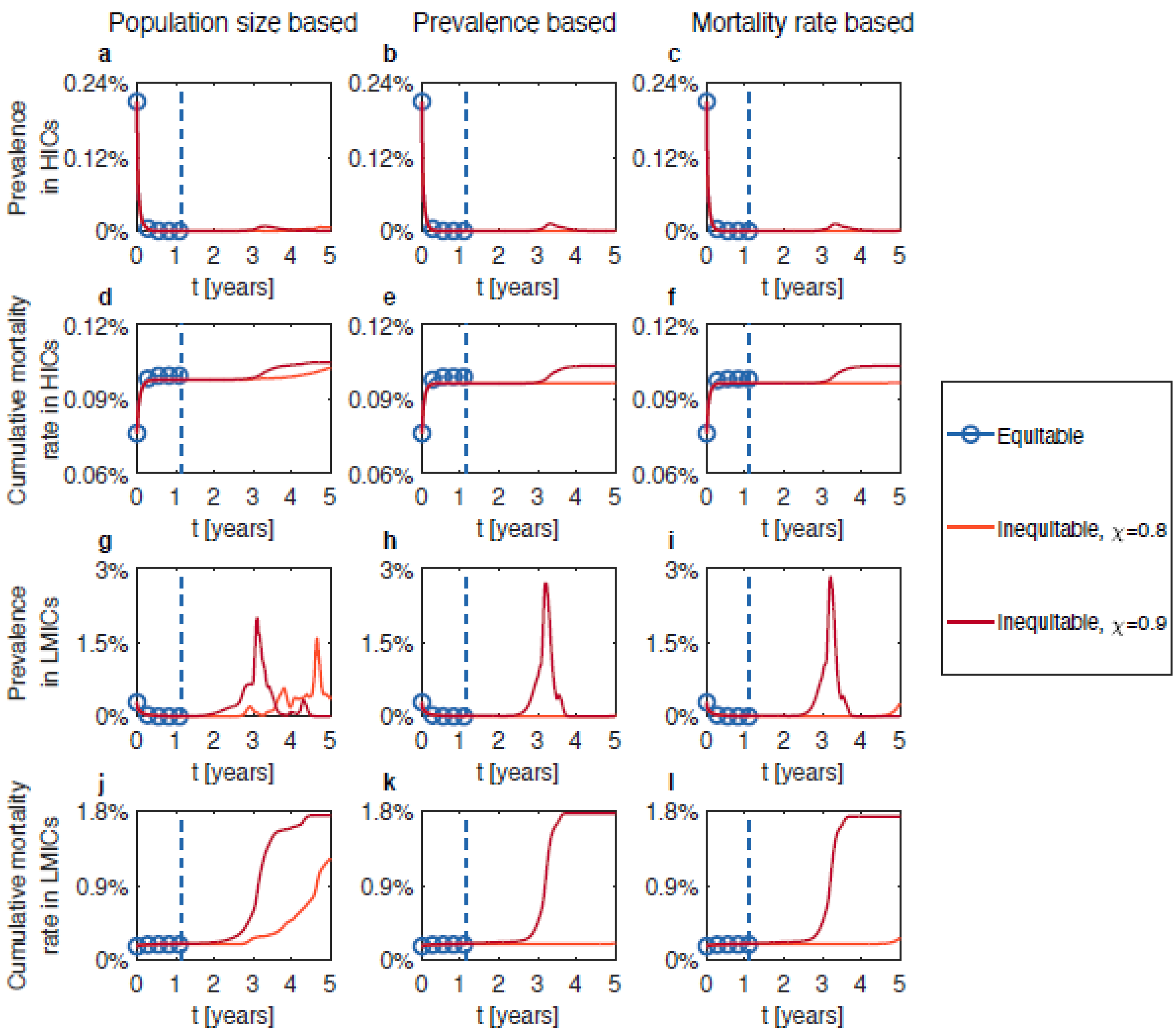

Figure 2: a-f, Time series of the prevalence (a-c) and the cumulative mortality rate (d-f) in HICs under different global vaccine allocation strategies. g-l, Time series of the prevalence (g-i) and the cumulative mortality rate (j-l) in LMICs under different global vaccine allocation strategies. Three prioritization criteria for allocation are adopted: the population size (the left panel), prevalence (the middle panel), and mortality rate (the right panel). Dash lines indicate the time when the pandemic ends (time exceeding five years is not presented). The transmissibility, severity of each strain, and the vaccine efficacy against each strain are shown in Fig. S2. Parameter values M=5, μ_1=5.6e-3, θ=0.2, and λ=500.

c. *What are panels k-o showing? Is this the fraction of new daily infections caused by strain m divided by the world population? I find it could be more interesting to show the share (%) of new daily cases due to the different strains (possibly on the same plot). I expect the curves to sum to 100% at each time step.*

RESPONSE 1-9c. Thank you for the suggestion. In the revised manuscript, we modified the labels of the y-axis for panels d-f (k-o in the initial submission) to "the ratio between the number of new cases and the world population." We added area plots showing the fraction of daily new cases produced by different strains (the cumulative sum of all areas is 100% at each time). Besides, for clearer presentation, panels k-

o in Figure 2 in the initial submission are now presented separately in Figure 3 in the revised manuscript. We copied the revised Figure 3 below.

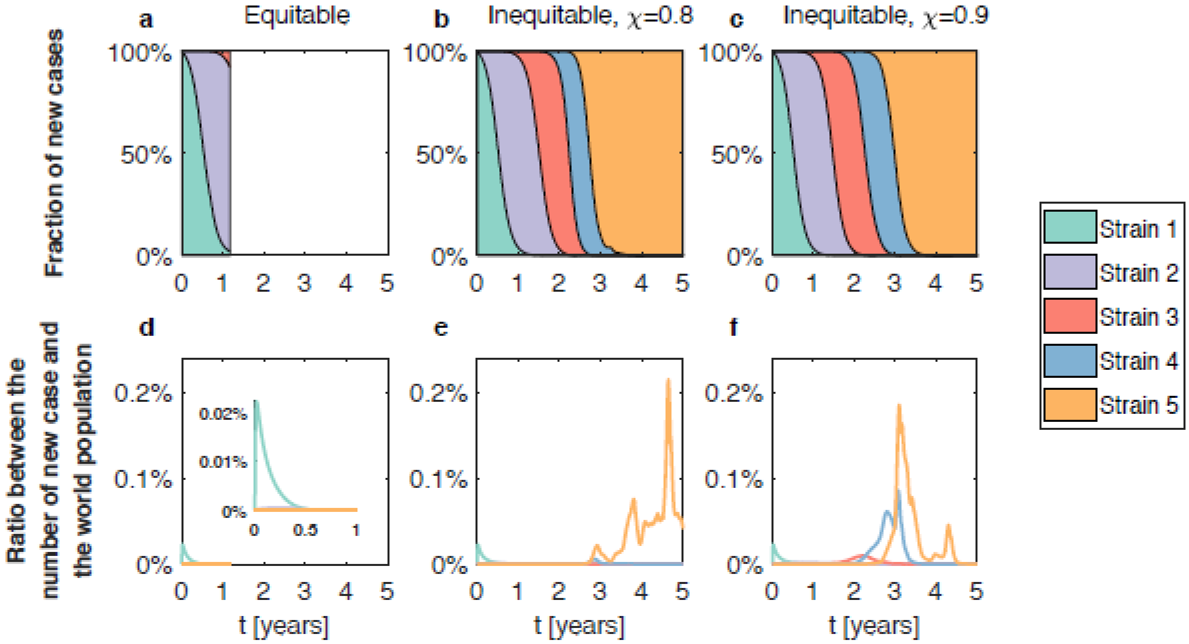

Figure 3: a-c, Area plots of the fraction of daily new cases produced by different strains. d-f, The ratio between the number of new cases produced by different strains and the world population. Figures in the left column, the middle column, and the right column are based on the equitable, inequitable and $\chi = 0.8$, and inequitable and $\chi = 0.9$ vaccine allocation strategies, respectively. All results are based on the prioritization criteria of the population size. The inset in subfigure d is the zoomed version of subfigure d. Parameter values M=5, μ_1=5.6e-3, θ=0.2, and λ=500.

d. *Figure 3 Panel b,c,e,f. How is this reduction/increase computed? Do the numbers in the right legend represent net increases or percentages?*

RESPONSE 1-9d. Thank you for the comment. In the initial submission, the numbers in the right legend represent the difference of cumulative cases (caused by different vaccine allocation strategies) as the share of the national population. In the revised manuscript, we provided a detailed description of how they are computed.

The added part is copied below:

Methods Section, **Calculation of the average lives saved by vaccine donations** Part: "Denote $\overline{D_{i,ineq}}$, $\overline{D_{i,eq}}$, and $\overline{D_{i,don}}$ as the cumulative mortality in country i at the end of the simulation under inequitable, equitable, and allow-donation vaccine allocation strategies, respectively. The average lives saved by vaccine donations (the difference between the cumulative mortality under allow-donation allocation strategy and that under inequitable allocation strategy) as the share of the national population in HICs and LMICs are denoted by r_H and r_L, respectively.

$$r_H = \frac{1}{|H|}\sum_{i\in H}\frac{\overline{D_{i,ineq}} - \overline{D_{i,don}}}{N_i},$$

$$r_L = \frac{1}{|L|}\sum_{i\in L}\frac{\overline{D}_{i,ineq} - \overline{D}_{i,don}}{N_i}.$$

Here, H and L denote the set of HICs and LMICs, respectively. $|H|$ and $|L|$ represent the number of HICs and LMICs, respectively."

The revised Figure 4 (Figure 3 in the initial submission) is copied below:

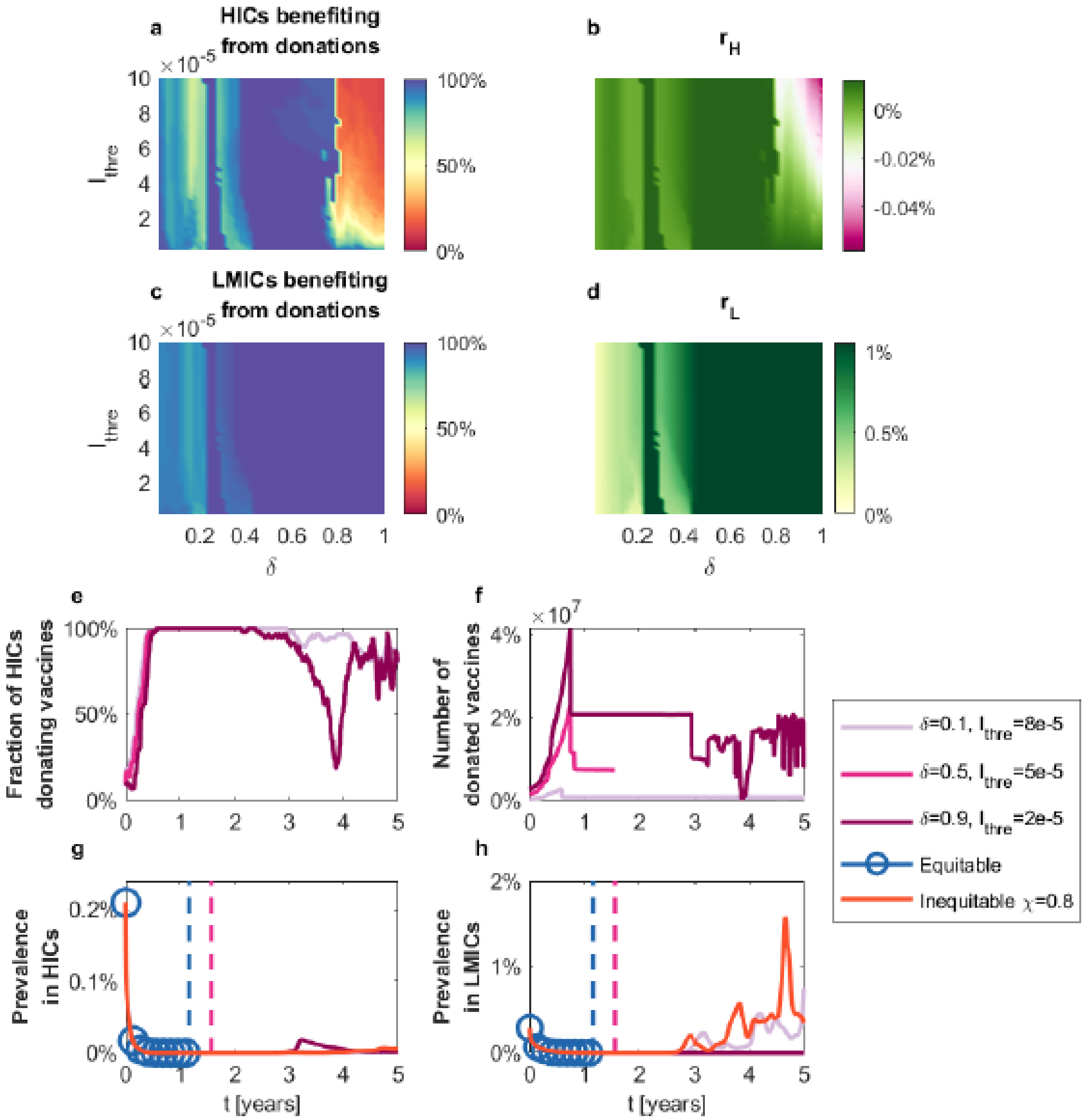

Figure 4: Impacts of different allow-donation vaccine allocation strategies on epidemic dynamics. a and c, Fraction of HICs and LMICs benefiting from donations. b and d, Average lives saved by vaccine donations as the share of the national population in HICs (r_H) and LMICs (r_L). Please refer to Methods

for details of r_H and r_L. e, Fraction of HICs donating vaccines. f, Total number of donated vaccines. g and h, Prevalence in HICs and LMICs under different vaccine allocation strategies. Countries with larger population sizes are prioritized for vaccination. Parameter values M=5, μ_1=5.6e-3, θ=0.2, and λ=500.

COMMENT 1-10.

a. A reference for the average case fatality ratio worldwide (0.02) should be added.

RESPONSE 1-10a. Thank you for the suggestion. The average case fatality ratio worldwide in the initial submission was the average of the case fatality ratios of all countries as of June 15, 2021. The data were obtained from the Johns Hopkins Coronavirus Resource Center [1]. As suggested by Reviewer 3, we set a country-specific severity (the case fatality rate) matrix in the revised manuscript to account for the heterogeneity in the healthcare burden of COVID-19 and the age structure in different countries. Then, we recalculated the country-specific case fatality rates using the Bayesian average instead of the global average. Denote the actual infection fatality rate for country i as IFR_i, then,

$$\mathcal{F}_{i,1} = IFR_i = \sum_a \mathcal{P}_i(deceased|infected, a)\frac{\mathcal{P}_i(infected|a)\mathcal{P}_i(a)}{\mathcal{P}_i(infected)}$$
$$\approx \sum_a CFR_{i,a}\frac{\mathcal{P}_i(infected|a)\mathcal{P}_i(a)}{\mathcal{P}_i(infected)},$$

where a denotes a specific age group, $\mathcal{P}_i(deceased|infected, a)$ represents the probability of dying from the disease for infected individuals at age group a, $CFR_{i,a}$ denotes the age-specific case fatality rate for age group a in country i, $\mathcal{P}_i(infected|a)$ represents the probability of getting infected for individuals in age group a in country i, $\mathcal{P}_i(infected)$ represents the probability of getting infected in country i, and $\mathcal{P}_i(a)$ represents the proportion of individuals in age group a, as a share of the whole population in country i. Due to limited data for age-specific case morbidity and fatality rates for a specific age group in each country, we set the same values of $CFR_{i,a}$ and $\mathcal{P}_i(infected|a)/\mathcal{P}_i(infected)$ among HICs and LMICs, i.e.,

$$CFR_{i,a} = \begin{cases} CFR_{H,a} & i \in H, \\ CFR_{L,a} & i \in L, \end{cases}$$

and

$$\frac{\mathcal{P}_i(infected|a)}{\mathcal{P}_i(infected)} = \begin{cases} \frac{\mathcal{P}_H(infected|a)}{\mathcal{P}_H(infected)} & i \in H, \\ \frac{\mathcal{P}_L(infected|a)}{\mathcal{P}_L(infected)} & i \in L. \end{cases}$$

Here, the values of $CFR_{H,a}$, $CFR_{L,a}$, $\frac{\mathcal{P}_H(infected|a)}{\mathcal{P}_H(infected)}$, and $\frac{\mathcal{P}_L(infected|a)}{\mathcal{P}_L(infected)}$ are computed based on data from India and the United States [1]. Specific values are shown in Tables S3 and S4. $\mathcal{P}_i(a)$ is adopted from the latest version of World Population Prospects [2]. Overall, the average case fatality rates among HICs and LMICs in the revised manuscript are 0.03 and 0.01, respectively. We added the following description in the main text to illustrate the variability in the case fatality rate (severity) across countries:

Method Section, **Multi-strain model** Part: "For country i, such dynamics are captured by the transmissibility matrix $\mathcal{T}$, the severity matrix $\mathcal{F}_i$, and the mutation matrix $\mathcal{U}$, all with dimensions $M \times M$…We set a country-specific severity matrix to account for the heterogeneity in the healthcare burden of COVID-19 and the age structure in different countries [3-5]."

Method Section, **Deterministic, discrete-time SVEIRD based metapopulation model** Part: "For individuals without vaccinal immunity, the transition rates from infectious (caused by strain m) to recovered and deceased are $(1 - \mathcal{F}_{i,m})\alpha$ and $\mathcal{F}_{i,m}\alpha$, respectively; for individuals with vaccinal immunity, the transition rates from infectious (caused by strain m) to recovered and deceased are $[1 - (1 - \epsilon_m)\mathcal{F}_{i,m})]\alpha$ and $(1 - \epsilon_m)\mathcal{F}_{i,m}\alpha$, respectively.

$$\partial_t R_i(t) = \sum_m (1 - \mathcal{F}_{i,m})\alpha I_{i,m}^S(t) + \sum_m [1 - (1 - \epsilon_m)\mathcal{F}_{i,m}]\alpha I_{i,m}^V(t) + \sum_j G_{ij}(t)[\frac{R_j(t)}{A_j(t)} - \frac{R_i(t)}{A_i(t)}],$$

$$\partial_t D_i(t) = \sum_m \mathcal{F}_{i,m}\alpha I_{i,m}^S(t) + \sum_m (1 - \epsilon_m)\mathcal{F}_{i,m}\alpha I_{i,m}^V(t).$$

"

A detailed description of the estimation of country-specific severity matrices could be found in Section 3 **Estimation of country-specific severity matrices** in the Supplementary Information.

References:

[1] Laxminarayan, R., Wahl, B., Dudala, S. R., Gopal, K., Neelima, S., Reddy, K. J., ... & Lewnard, J. A. (2020). Epidemiology and transmission dynamics of COVID-19 in two Indian states. Science, 370(6517), 691-697.

[2] The United Nations. United Nations World Population Prospects. (2020). https://population.un.org/wpp/

[3] Miller, I. F., Becker, A. D., Grenfell, B. T., & Metcalf, C. J. E. (2020). Disease and healthcare burden of COVID-19 in the United States. Nature Medicine, 26(8), 1212-1217.

[4] O'Driscoll, M., Dos Santos, G. R., Wang, L., Cummings, D. A., Azman, A. S., Paireau, J., ... & Salje, H. (2021). Age-specific mortality and immunity patterns of SARS-CoV-2. Nature, 590(7844), 140-145.

[5] Zhang Qingpeng. (2022). Data science approaches to infectious disease surveillance. Phil. Trans. R. Soc. A. 380(2214) 20210115.

b. I do not see significant differences in results obtained using prioritization based on incidence and prevalence. This is somehow expected. I would simplify figures in the main text and place results on one of the two in the Supplementary Information.

RESPONSE 1-10b. Thank you for the suggestion. In the revised manuscript, we placed all results using prioritization based on incidence in the Supplementary Information. We added a mortality rate-based strategy in the main text according to the suggestion by Reviewer 3. The complete version of Figure 2 in the main text (containing results based on the prioritization criteria of the incidence) is Figure S3 in the Supplementary Information. Figure S3 is copied below:

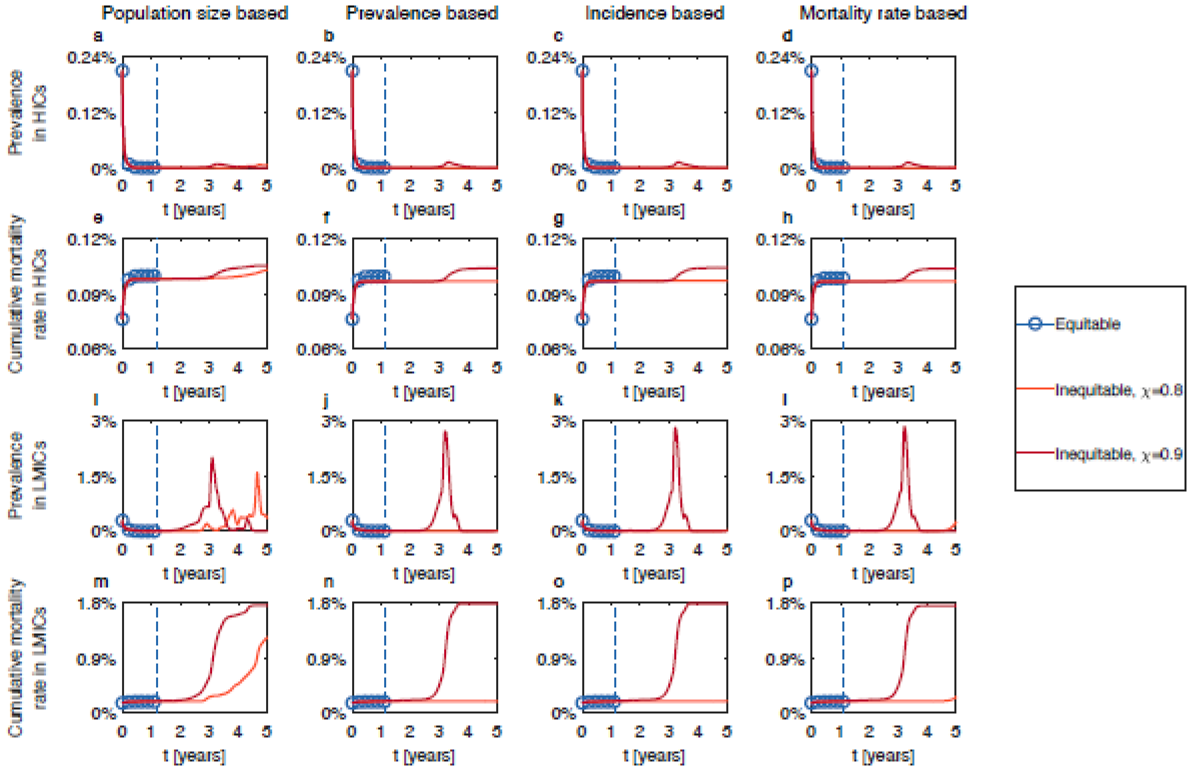

Figure S3: a-h, Time series of the prevalence (a-d) and the cumulative mortality rate (e-h) in HICs under different global vaccine allocation strategies. i-p, Time series of the prevalence (i-l) and the cumulative mortality rate (m-p) in LMICs under different global vaccine allocation strategies. Four prioritization criteria for allocation are adopted: the population size (the left panel), prevalence (second left panel), incidence (second right panel), and mortality rate (the right panel). Dash lines indicate the time when the pandemic ends (time exceeding five years is not presented). Parameter values M=5, μ_1=5.6e-3, θ=0.2, and λ=500.

c. It is known that COVID-19 severity strongly increases with ages. This could be one of the reasons why HICs countries, characterized by older populations, have been strongly hit by the pandemic, even in the presence of advanced and efficient health care systems, while in some other LMICs COVID-19 burden appears relatively low (see e.g. Trentini et al, BMC Medicine, 2021). Please acknowledge that one of the main limitations of your approach is that your model is not stratified by age.

RESPONSE 1-10c. Thank you for the suggestion. We agree with the reviewer that COVID-19 deaths have been concentrated at older ages. The model would be more realistic by considering the differences in age structure across countries. To address this issue, in the revised manuscript, we set a country-specific severity matrix to characterize the heterogeneity in the healthcare burden of COVID-19 and the age structure worldwide (see **RESPONSE 1-10a**). Due to the lack of real-world data, we did not incorporate the differences in the age-specific contact patterns across the world. Following the reviewer's suggestion, we added the following statement in the revised manuscript to acknowledge this limitation:

Conclusion Section: "First, although we derive a country-specific severity matrix to model the heterogeneous age structures across different countries, the model is not stratified by age within each country. The difference in age structures results in heterogeneous infection fatality rates and heterogeneous susceptibility to infection [1-4]. Due to limited data of the susceptibility to infection

among different age groups, and the lack of age-mixing patterns for different countries, we do not parameterize an age-stratified model for each country. The model can be easily calibrated if such data was available."

References:

[1] Davies, N. G., Klepac, P., Liu, Y., Prem, K., Jit, M., & Eggo, R. M. (2020). Age-dependent effects in the transmission and control of COVID-19 epidemics. Nature medicine, 26(8), 1205-1211.

[2] Trentini, F., Guzzetta, G., Galli, M., Zardini, A., Manenti, F., Putoto, G., ... & Poletti, P. (2021). Modeling the interplay between demography, social contact patterns, and SARS-CoV-2 transmission in the South West Shewa Zone of Oromia Region, Ethiopia. BMC medicine, 19(1), 1-13.

[3] Zhang Qingpeng. (2022). Data science approaches to infectious disease surveillance. Phil. Trans. R. Soc. A. 380(2214) 20210115.

[4] Burghardt Keith, Guo Siyi, & Lerman Kristina. (2022). Unequal impact and spatial aggregation distort COVID-19 growth rates Phil. Trans. R. Soc. A. 380(2214) 20210122.

d. Pag.9: reference to Figure 3b in the text. Do you mean 3d?

RESPONSE 1-10d. Thank you for pointing out the typo. We have corrected it in the revised manuscript. Figure 4 in the revised manuscript corresponds to Figure 3 in the initial submission.

Results Section, **Vaccine donation is a practical pathway to global vaccine equity** Part: "Unsurprisingly, almost all LMICs benefit from vaccine donations regardless of when and how many vaccines are donated by HICs (Fig. 4**c**)."

e. Pag.9. "Either a larger δ or a larger I_thre results in a larger reduction in cumulative cases in LMICs (Fig. 3e and f), which means the larger proportion of vaccines they share, the fewer people in LMICs will be infected.". Looking at the Figure, I_thre apparently play no role (or a very limited role). I would modify to: "Larger δ results in a larger reduction in cumulative cases in LMICs (Fig. 3e and f), which means the larger proportion of vaccines they share, the fewer people in LMICs will be infected."

RESPONSE 1-10e. Thank you for the suggestion. We have modified this statement as suggested.

The revised part is copied below.

Results Section, **Vaccine donation is a practical pathway to global vaccine equity** Part: "More vaccines donated by HICs result in a larger reduction in the cumulative mortality in LMICs (Fig. 4**d**)."

f. Pag 15: "We have proposed a mathematical model to investigate both the short-term and long-term impacts of vaccine equity taking account of immune escape and global transportation.". Apparently, the authors are not including immune escape (hosts recovered from either strain are immune to all other strains). Please specify.

RESPONSE 1-10f. Thank you for the suggestion. We have modified this statement as follows:

Conclusion Section: "We propose a multi-strain metapopulation model to investigate the short-term and long-term impacts of vaccine equity taking account of viral mutations and global transportation."

g. Results (pag.7): "In these new waves, infections in HICs are largely due to imported cases from LMICs.". Could you clarify if this assumption is based on your model outcome? Can the model separately keep track of secondary cases generated by imported infections?

RESPONSE 1-10g. Thank you for the comment. In the first four years, LMICs account for the majority of new cases (Fig. S5). Since each infection represents a chance of viral mutation, the probability of emerging new strains in LMICs is much higher than that in HICs. Thus, the onset of new waves of the disease in HICs is mainly caused by the higher probability of emerging new strains in LMICs. Since our model is not an agent-based model, accurately tracking secondary cases generated by individual imported infections is infeasible. To avoid misunderstanding, we revised this statement to the following:

Results Section, **Global vaccine inequity only provides limited and short-term benefits to HICs** Part: "The onset of new waves of the disease in HICs is mainly caused by the higher probability of emerging new strains in LMICs. In the first four years, LMICs account for the majority of cases (Fig.~S5). Since each infection represents a chance of viral mutation, the probability of emerging new strains in LMICs is much higher than that in HICs."

Figure S5 is copied below:

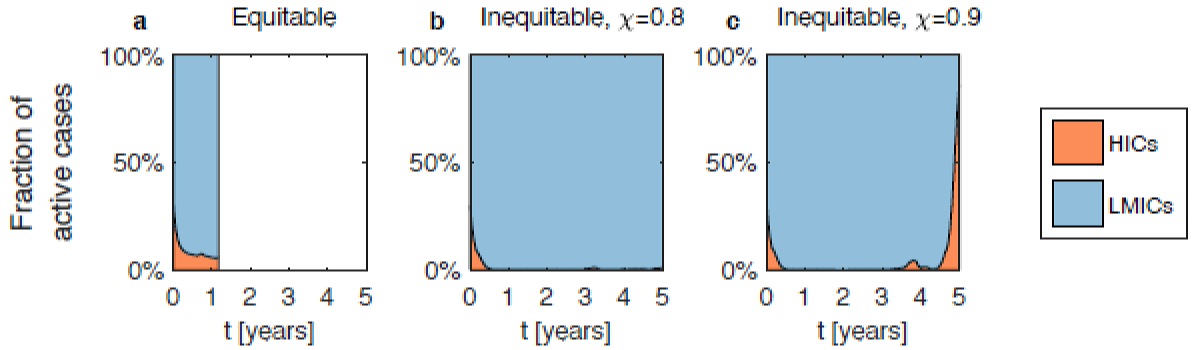

Figure S5: Area plots of the fraction of active cases in HICs and LMICs. Figures in the left column, the middle column, and the right column are based on the equitable, inequitable and χ=0.8, and inequitable and χ=0.9 vaccine allocation strategies, respectively. All results are based on the prioritization criteria of the population size. Parameter values M=5, μ_1=5.6e-3, θ=0.2, and λ=500.

REVIEWER 2'S COMMENTS

This paper aims to understand, through a multi-strain metapopulation mathematical model, how vaccine equity for COVID-19 can impact its global epidemiology. Briefly, it shows that vaccine unequity can only provide short term benefits to the HICs and that vaccine donations is the best strategy to decrease COVID-19 burden. I think the paper is interesting, timely, and deserves to be published when my comments would have been included.

COMMENT 2-1. *My main concern is about the connectivity between LMICs and HICs which is not explicitly mentioned. However, this connectivity network is far from random and change impact dramatically their conclusion. I can understand that is not addressed explicitly, but it needs to be carefully discussed.*

RESPONSE 2-1. Thank you for the comment. The connectivity (γ and P_{ij}) is obtained based on the real-world air traffic data in 2020 from OAG (https://www.oag.com/). We added a detailed description of generating these two values in the revised manuscript to avoid confusion. A visualization of this global mobility network is added to the Supplementary Information (and Github page). The added parts are copied below:

In the main text:

Method Section, **Deterministic, discrete-time SVEIRD based metapopulation model** Part: "Denote F_{ij} as the number of passengers traveling from country i to country j per day, and $F_i = \sum_j F_{ij}$. Then $P_{ij} = F_{ij}/F_i$. We obtain F_{ij} by averaging the aggregated number of seats on scheduled commercial flights between country i to country j per day in 2020 (Fig. S27)."

In the Supplementary Information:

Section 4 **Model parameter settings**:

"P_{ij} and γ are computed based on the air traffic data in 2020 from OAG [1]. γ=0.00015 is the average (inflow/outflow) mobility rate per person per day in 2020 [2]."

The added figure is copied below:

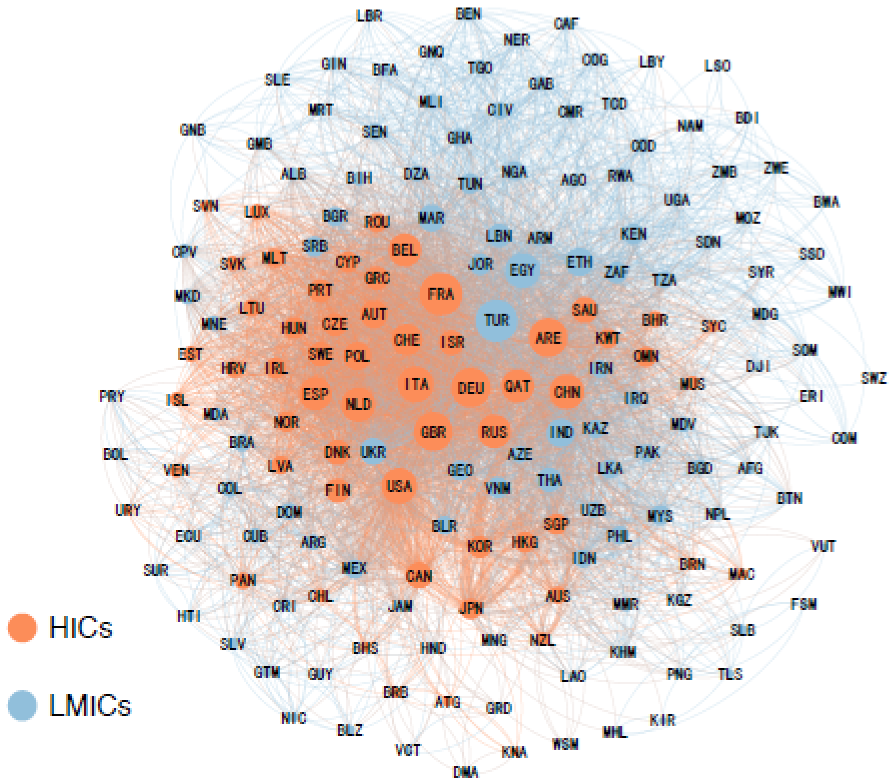

Figure S27: The global mobility network. Nodes represent countries. Edges represent the aggregated number of seats on scheduled commercial flights between countries per day. The size of a node is proportional to the number of neighboring countries (countries that are reachable via direct flights). Only 3-letter ISO codes for countries are presented for a clear illustration.

References:

[1] OAG. OAG(2020). https://www.oag.com/.https://www.oag.com/

[2] Yang Ye, Qingpeng Zhang, Zhidong Cao, Frank Youhua Chen, Houmin Yan, H Eugene Stanley, and Daniel Dajun Zeng. Impacts of export restrictions on the global personal protective equipment trade netw ork during COVID-19. Advanced Theory and Simulations. https://www.doi.org/10.1002/adts.202100352

COMMENT 2-2. *The second concern is about the lack of references to other works on that topic. Indeed, there are several papers discussing this topic (on other pathogens) and they have to clearly cited and discussed.*

RESPONSE 2-2. Thanks for your suggestion. We have added and discussed several papers about this topic on other pathogens in the introduction part. The added part is copied below:

Introduction Section:

"Such game-theoretic approach has been applied to the control of other pathogens [1-6]. Numerical and analytical results based on hypothetical networks show that the optimal drug/vaccine coordination can

reduce the epidemic size and overall financial burden of infection for all countries. However, data-driven research on global vaccine coordination in real-world human mobility networks is rare, particularly in the context of the COVID-19 pandemic with viral mutations."

References:

[1] Mamani, H., Chick, S. E., & Simchi-Levi, D. (2013). A game-theoretic model of international influenza vaccination coordination. Management Science, 59(7), 1650-1670.

[2] Sun, P., Yang, L., & De Véricourt, F. (2009). Selfish drug allocation for containing an international influenza pandemic at the onset. Operations Research, 57(6), 1320-1332.

[3] Aswani, A., Shen, Z. J. M., & Siddiq, A. (2019). Data-driven incentive design in the medicare shared savings program. Operations Research, 67(4), 1002-1026.

[4] Klepac, P., Laxminarayan, R., & Grenfell, B. T. (2011). Synthesizing epidemiological and economic optima for control of immunizing infections. Proceedings of the National Academy of Sciences, 108(34), 14366-14370.

[5] Matrajt, L., Halloran, M. E., & Longini Jr, I. M. (2013). Optimal vaccine allocation for the early mitigation of pandemic influenza. PLoS Computational Biology, 9(3), e1002964.

[6] Barrett, S. (2013). Economic considerations for the eradication endgame. Philosophical Transactions of the Royal Society B: Biological Sciences, 368(1623), 20120149.

COMMENT 2-3. *Finally, my last major concern is about the initialisation of the simulation, especially regarding the number of strains. From what I've understood, it starts with 5 strains but different initial conditions can produce very different outcomes.*

RESPONSE 2-3. Thanks for your valuable comment. We agree with the reviewer that different settings on the number of strains can produce different outcomes. Reviewer 1 and editors also suggested that we perform sensitivity analysis of the mutation-related parameters, M, θ, μ_1, and λ. Following all these suggestions, we used the historical data for the first 1.5 years of the pandemic (from December 31, 2019, to June 15, 2021) to inform the values of these parameters. We assumed the virus would follow a similar mutation process in the future and performed sensitivity analysis on these parameters. Results of the sensitivity analysis were similar to those reported in the main manuscript. Please refer to the description below and Fig. S6-Fig. S14 and Fig. S20-Fig. S24 in the Supplementary Information for details.

The number of "Variants of Concern" (M) emerged in the next five years: Most of the viral mutations have little impact on the virus' ability to transmit and cause severe infections. Variants meeting specific criteria (e.g., increase in transmissibility, increase in virulence, decrease in the effectiveness of public health measures.) are designated as "Variants of Concern" by the World Health Organization [1]. Although there are thousands of genetic variants of SARS-CoV-2 [2], only four of them are designated as "Variants of Concern" as of June 15, 2021, i.e., the Alpha, Beta, Gamma, and Delta strain. Thus, the value of M for the first 1.5 years of the pandemic is 5. In the revised manuscript, we assumed M ranges from 3 to 10 in the next five years.

The increase in the transmissibility of each new strain (θ): The most transmissible strain, Delta, demonstrates 2.5 times higher transmissibility than the original strain. Therefore, here we assume a linear strain space and local movement by a one-direction stepwise mutation [3], then the transmissibility of

each new strain is 26% ($1.26^4 \approx 2.5$) higher than the immediate previous strain, i.e., θ=0.26 for the first 1.5 years of the pandemic. In the revised manuscript, we assumed θ ranges from 0.15 to 0.5 in the next five years.

The mutation probability per infection of the strain at time 0 (μ_1), and decrease rate of the mutation probability per infection (λ): There is evidence that mutations in the genome region encoding the spike protein (3822 nucleotides in length from site 21563 to 25384 [4]) may result in increased transmissibility [5] and viral load [6] of the virus. It is estimated that the mutation rate per virus replication cycle per site is 3×10^{-6}, and the entire course of infection will take approximately five viral replication cycles [7]. Thus, the probability that the spike protein region changes per infection is $1-((1-3\times10^{-6})^5)^{3822}\approx0.056$. Most of such mutations are neutral. Moreover, the virus cannot evolve indefinitely, primarily because each nucleotide can only mutate to three others (e.g., adenine (A) can only mutate to thymine (T) or guanine (G) or cytosine (C)), and we have limited number of nucleotides [8]. As the virus evolves in the strain space, the probability of major and new changes per infection decreases because fewer possible genome sequences remain. It means that the probability of emerging new and more dangerous strains per infection decreases over time [9]. Based on these facts, we assume that (a) for strain 1, only 1‱~10% of such mutations can significantly affect the virus' ability to cause infections, i.e., μ_1 ranges from 5.6×10^{-6} to 5.6×10^{-3} in the next five years; (b) $\mu_{m+1} = \mu_m/\lambda$, where λ quantifies the decrease rate of the probability of emerging new and more dangerous strains per infection. λ ranges from 10^2 to 10^4 in the next five years.

The revised parts are copied below:

Method Section, **Multi-strain model** Part: "The virus in strain m can either remains as strain m with probability $1-\mu_m$ or mutates to strain $m+1$ (one-direction stepwise mutation) with probability μ_m while adapting to a new host (please refer to the Supplementary Information for details of the spreading process), thus, we construct $\mathcal{U}$ as

$$\mathcal{U} = \begin{bmatrix} 1-\mu_1 & \mu_1 & \cdots & 0 & 0 \\ 0 & 1-\mu_2 & \mu_2 & \cdots & 0 \\ \vdots & \vdots & \vdots & \ddots & \vdots \\ 0 & 0 & 0 & \cdots & 1 \end{bmatrix}.$$

The virus cannot evolve indefinitely, primarily because each nucleotide can only mutate to three others (e.g., adenine can only mutate to thymine or guanine or cytosine), and the genome of SARS-CoV-2 has limited nucleotides in length [7, 8]. As the virus evolves in the strain space, the probability of major and new changes per infection decreases because fewer possible genome sequences remain. Thus, we assume $\mu_{m+1} = \mu_m/\lambda$, where λ decrease rate of the probability of emerging new and more dangerous strains…specifically, we assume $\mathcal{T}_{n+1} = (1+\theta)\mathcal{T}_n$."

Section 4 **Model parameter settings** in the Supplementary Information: "Because of limited data to quantify the viral mutation parameters, M, θ, μ_1, and λ, we use the historical data for the first 1.5 years of the pandemic (from December 31, 2019, to June 15, 2021) to inform the values of these parameters. We assume the virus follows a similar mutation process in the future and perform sensitivity analysis on these parameters (Fig. S6-S14, Fig. S20-S24)."

References:

[1] WHO. Tracking SARS-CoV-2 variants (2021). https://www.who.int/en/activities/tracking-SARS-CoV-2-variants/

[2] Rambaut, A., Holmes, E. C., O'Toole, Á., Hill, V., McCrone, J. T., Ruis, C., ... & Pybus, O. G. (2020). A dynamic nomenclature proposal for SARS-CoV-2 lineages to assist genomic epidemiology. Nature microbiology, 5(11), 1403-1407.

[3] Gog, J. R., & Grenfell, B. T. (2002). Dynamics and selection of many-strain pathogens. Proceedings of the National Academy of Sciences, 99(26), 17209-17214.

[4] Yang, H. C., Chen, C. H., Wang, J. H., Liao, H. C., Yang, C. T., Chen, C. W., ... & Liao, J. C. (2020). Analysis of genomic distributions of SARS-CoV-2 reveals a dominant strain type with strong allelic associations. Proceedings of the National Academy of Sciences, 117(48), 30679-30686.

[5] Li, Q., Wu, J., Nie, J., Zhang, L., Hao, H., Liu, S., ... & Wang, Y. (2020). The impact of mutations in SARS-CoV-2 spike on viral infectivity and antigenicity. Cell, 182(5), 1284-1294.

[6] Frampton, D., Rampling, T., Cross, A., Bailey, H., Heaney, J., Byott, M., ... & Nastouli, E. (2021). Genomic characteristics and clinical effect of the emergent SARS-CoV-2 B. 1.1. 7 lineage in London, UK: a whole-genome sequencing and hospital-based cohort study. The Lancet Infectious Diseases, 21(9), 1246-1256.

[7] Sender, R., Bar-On, Y. M., Gleizer, S., Bernshtein, B., Flamholz, A., Phillips, R., & Milo, R. (2021). The total number and mass of SARS-CoV-2 virions. Proceedings of the National Academy of Sciences, 118(25).

[8] Burioni, R., & Topol, E. J. (2021). Has SARS-CoV-2 reached peak fitness?. Nature Medicine, 27 (8), 1323–1324.

[9] Kupferschmidt, K. (2021). Evolving threat. Science, 373(6557) 844-849. https://doi.org/10.1126/science.373.6557.844.

COMMENT 2-4. *Figure 2 is complicated to read. Moreover, axes are not consistent. A summary Figure would be better.*

RESPONSE 2-4. Thanks for your valuable comment. For clearer visualization, we split Figure 2 into four figures (Figure 2 and Figure 3 in the main text; Figure S2 and Figure S4 in the Supplementary Information) in the revised manuscript for better understanding. According to the suggestion from Reviewer 1, we present both the prevalence and the cumulative mortality rate in HICs and LMICs under different global vaccine allocation strategies in Figure 2 to provide more information about the COVID-19 burden of deaths. The modified Figure 2 is copied below:

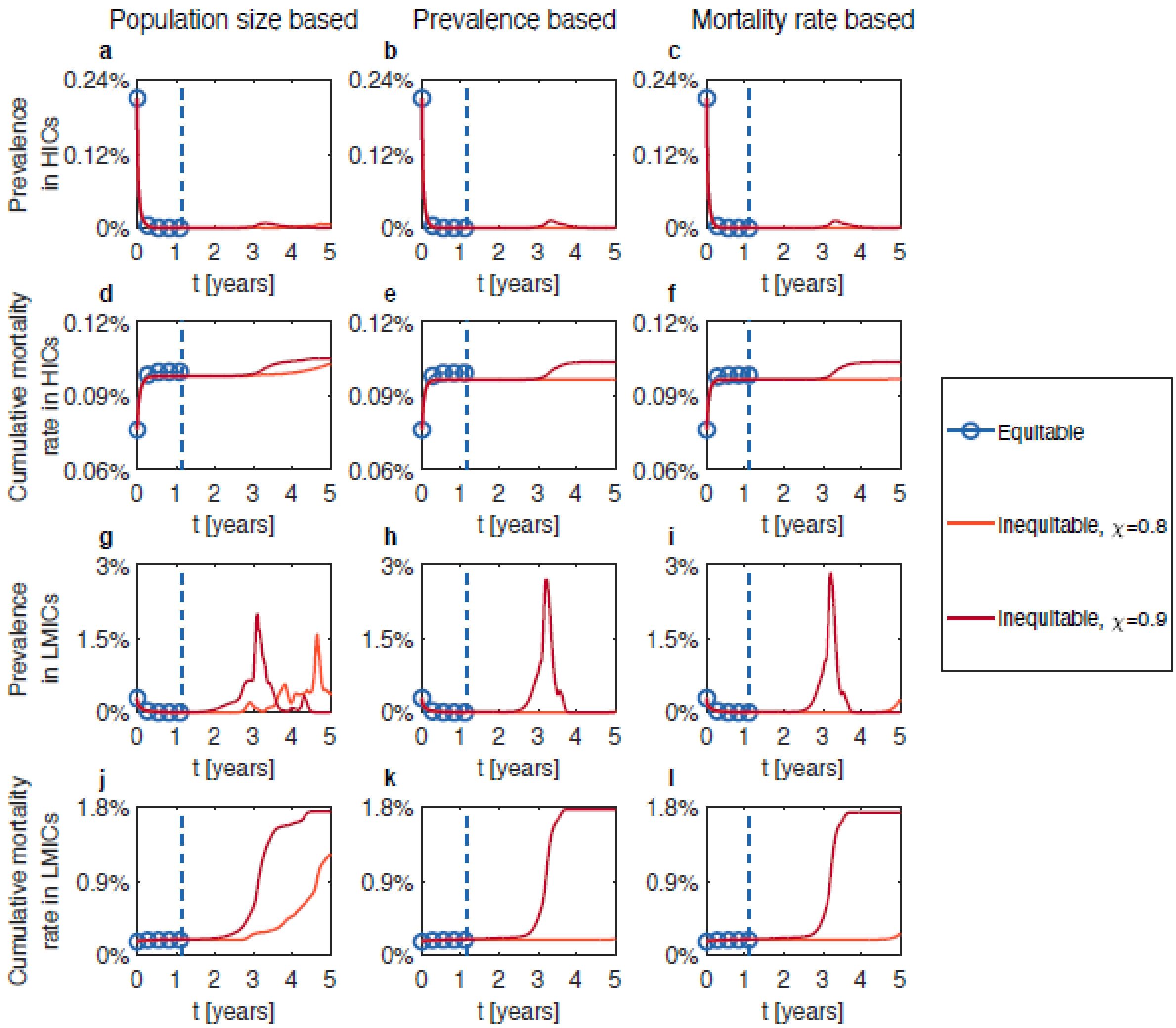

Figure 2: a-f, Time series of the prevalence (a-c) and the cumulative mortality rate (d-f) in HICs under different global vaccine allocation strategies. g-l, Time series of the prevalence (g-i) and the cumulative mortality rate (j-l) in LMICs under different global vaccine allocation strategies. Three prioritization criteria for allocation are adopted: the population size (the left panel), prevalence (the middle panel), and mortality rate (the right panel). Dash lines indicate the time when the pandemic ends (time exceeding five years is not presented). The transmissibility, severity of each strain, and the vaccine efficacy against each strain are shown in Fig. S2. Parameter values M=5, μ_1=5.6e-3, θ=0.2, and λ=500.

COMMENT 2-5. *Axes labels on Figure 3 need to be clearer.*

RESPONSE 2-5. Thanks for the suggestion. Figure 3 in the initial submission is now Figure 4 in the revised manuscript. In the revised manuscript, we provided a detailed description of the axes labels on Figure 4.

The added part is copied below:

Methods Section, **Calculation of the average lives saved by vaccine donations** Part: "Denote $\overline{D}_{i,ineq}$, $\overline{D}_{i,eq}$, and $\overline{D}_{i,don}$ as the cumulative mortality in country i at the end of the simulation under inequitable, equitable, and allow-donation vaccine allocation strategies, respectively. The average lives saved by vaccine donations (the difference between the cumulative mortality under allow-donation allocation strategy and that under inequitable allocation strategy) as the share of the national population in HICs and LMICs are denoted by r_H and r_L, respectively.

$$r_H = \frac{1}{|H|}\sum_{i\in H}\frac{\overline{D}_{i,ineq} - \overline{D}_{i,don}}{N_i},$$

$$r_L = \frac{1}{|L|}\sum_{i\in L}\frac{\overline{D}_{i,ineq} - \overline{D}_{i,don}}{N_i}.$$

Here, H and L denote the set of HICs and LMICs, respectively. $|H|$ and $|L|$ represent the number of HICs and LMICs, respectively."

COMMENT 2-6. *Frequently, parameter symbols are mentioned inside the text. It is better to avoid that since it adds confusion.*

RESPONSE 2-6. Thanks for the suggestion. We have tried to delete parameter symbols inside the text to avoid confusion.

REVIEWER 3'S COMMENTS

The study is a very important and well-written paper aiming to measure inequity in COVID-19 vaccination distribution. The authors developed a mathematical model that explicitly considers 1) the inequity in vaccine distribution and 2) the viral evolutionary dynamics and their effects on vaccine efficacy. Their key finding suggests that vaccine inequity only provides limited and short-term benefits to HICs, leading to a moderate increase in infections and deaths in LMICs. The work is timely and is of particular interest nowadays, with the initiation of the third booster dose in several countries. I find their mathematical model clear, transparent, and elegant.

COMMENT 3-1. *It is clear that there is a waning of both natural and vaccine immunity. It is not being considered in their model and might affect their results.*

RESPONSE 3-1. Thank you very much for the comment. In the initial submission, our model takes into account the waning of vaccine immunity, but not the natural immunity, because reinfections are rare in the general population [3-6]. In the revised manuscript, we added the sensitive analysis of the potential natural immunity.

Specifically, the vaccinal immunity is described in the **Methods** section:

"We assume vaccinated individuals gradually lose vaccinal immunity and become fully susceptible again at the rate ε."

And Section 4 **Model parameter settings** in the Supplementary Information:

"Currently, the duration of vaccinal immunity remains unclear. We set $1/\varepsilon$ =365 (days) based on publicly available clinical trial data [1, 2]."

According to the recent literature [3-6], reinfections are uncommon in the general population:

[3] (published on May 24, 2021) "Overall, our results indicate that mild infection with SARS-CoV-2 induces robust antigen-specific, long-lived humoral immune memory in humans."

[4] (published on May 28, 2021) "The study results suggest that reinfections are rare events and patients who have recovered from COVID-19 have a lower risk of reinfection."

[5] (published on August 26, 2021) "The newly released data show people who once had a SARS-CoV-2 infection were much less likely than never-infected, vaccinated people to get Delta, develop symptoms from it, or become hospitalized with serious COVID-19."

[6] (published on September 13, 2021) "Prior infection decreased risk of symptomatic reinfection by 93%......This implies a prolonged (perhaps years) capacity to respond to new infections with new antibodies."

So in the initial submission, we did not consider the waning of natural immunity. In the revised manuscript, we added a sensitivity analysis to explore how the waning of natural immunity may affect our results and added this as one of the main limitations of our model in the **Conclusion** section in the main text.

The added part is copied below:

Conclusion section in the main text:

"Second, according to the findings that reinfections are uncommon in the general population [3-6], we investigated the life-long and different short-lived natural immunity settings. Sensitivity analysis (Fig. S25 and S26) shows that, if natural immunity is short-lived, global vaccine inequity provides even smaller benefits to HICs. Future research should incorporate the more realistic natural immunity duration data for different strains (if available)."

The added figures are copied below:

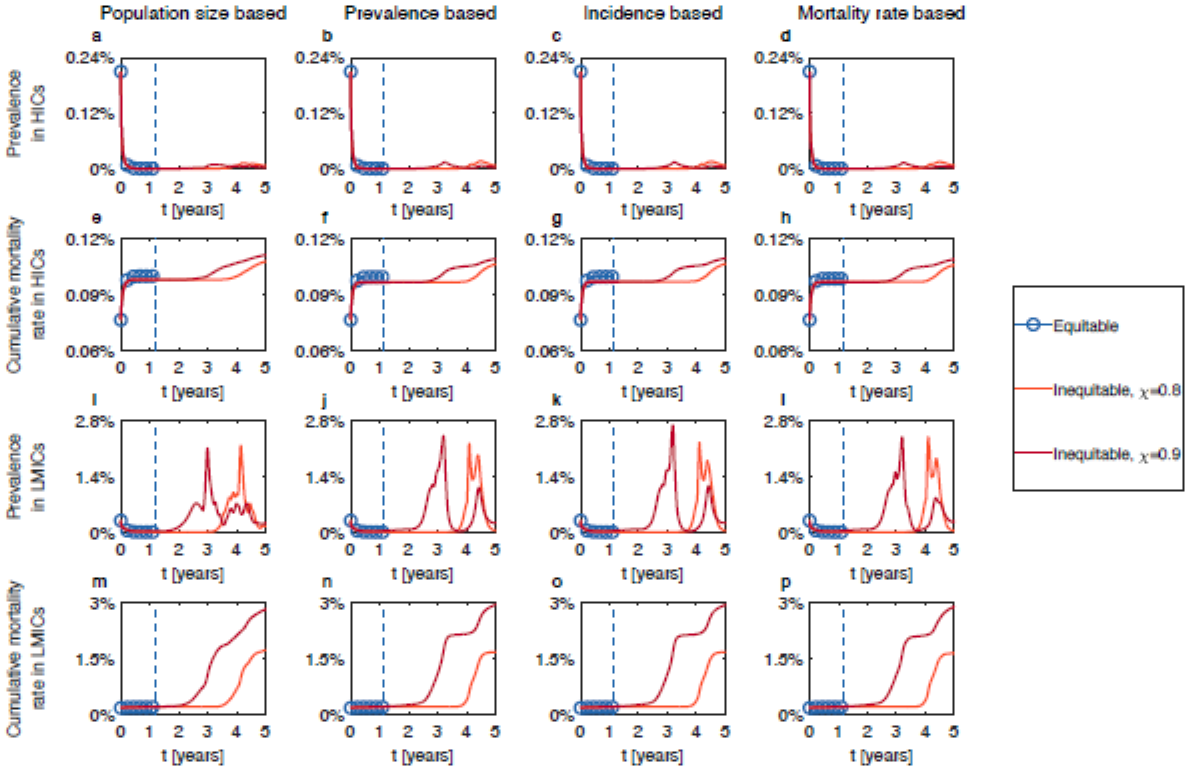

Figure S25: a-h, Time series of the prevalence (a-d) and the cumulative mortality rate (e-h) in HICs under different global vaccine allocation strategies. i-p, Time series of the prevalence (i-l) and the cumulative mortality rate (m-p) in LMICs under different global vaccine allocation strategies. Four prioritization criteria for allocation are adopted: the population size (the left panel), prevalence (second left panel), incidence (second right panel), and mortality rate (the right panel). Dash lines indicate the time when the pandemic ends (time exceeding five years is not presented). The duration of natural immunity is two years. Parameter values M=5, μ_1=5.6e-3, θ=0.2, and λ=500.

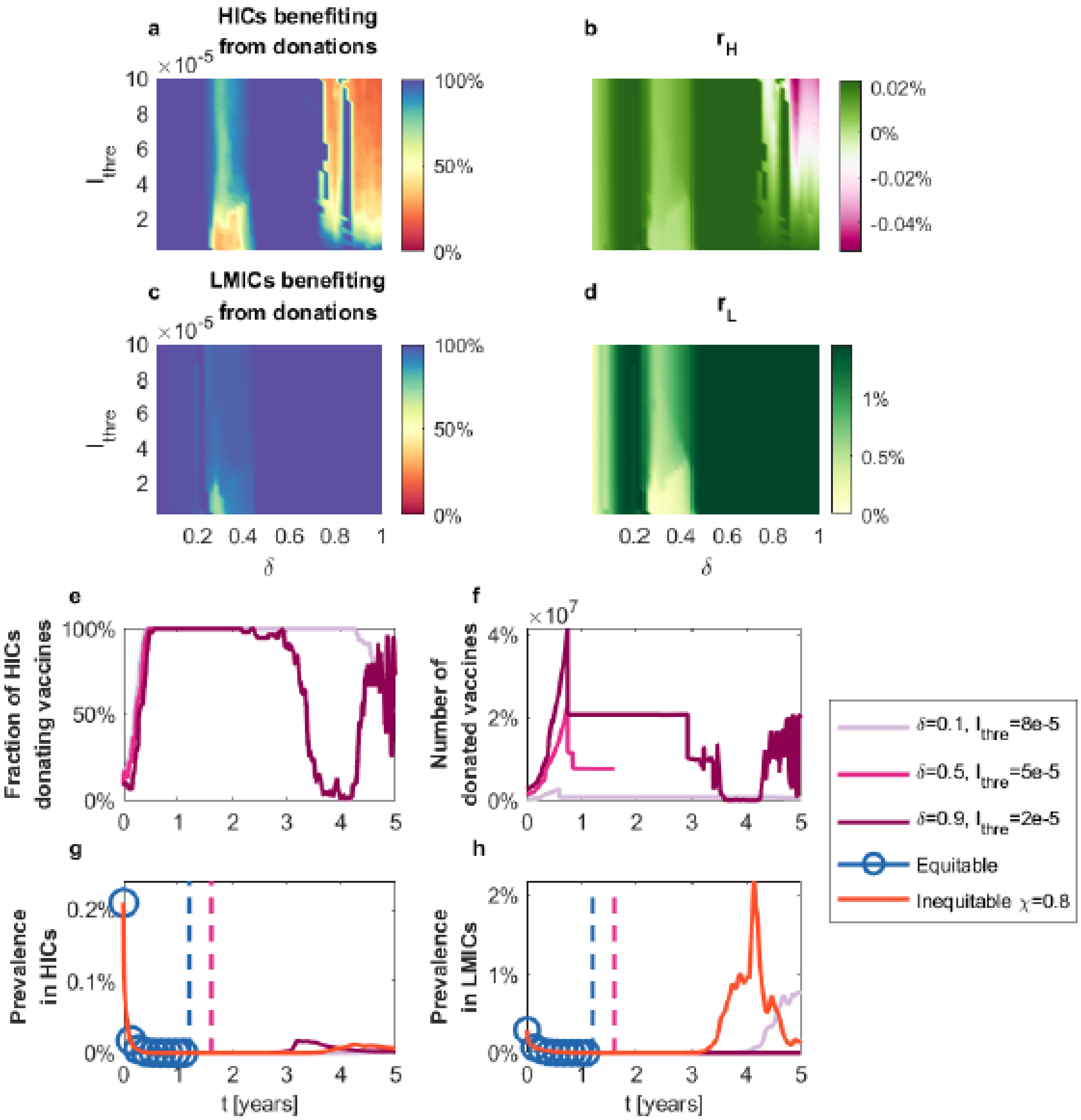

Figure S26: Impacts of different allow-donation vaccine allocation strategies on epidemic dynamics. a and c, Fraction of HICs and LMICs benefiting from donations. b and d, Average lives saved by vaccine donations as the share of the national population in HICs (r_H) and LMICs (r_L). e, Fraction of HICs donating vaccines. f, Total number of donated vaccines. g and h, Prevalence in HICs and LMICs under different vaccine allocation strategies. Countries with larger population sizes are prioritized for vaccination. The duration of natural immunity is two years. Parameter values M=5, μ_1=5.6e-3, θ=0.2, and λ=500.

References:

[1] Anderson, R. M., Vegvari, C., Truscott, J., & Collyer, B. S. (2020). Challenges in creating herd immunity to SARS-CoV-2 infection by mass vaccination. The Lancet, 396(10263), 1614-1616.

[2] Dan, J. M., Mateus, J., Kato, Y., Hastie, K. M., Yu, E. D., Faliti, C. E., ... & Crotty, S. (2021). Immunological memory to SARS-CoV-2 assessed for up to 8 months after infection. Science, 371(6529).

[3] Turner, J. S., Kim, W., Kalaidina, E., Goss, C. W., Rauseo, A. M., Schmitz, A. J., ... & Ellebedy, A. H. (2021). SARS-CoV-2 infection induces long-lived bone marrow plasma cells in humans. Nature, 1-5.

[4] Vitale, J., Mumoli, N., Clerici, P., De Paschale, M., Evangelista, I., Cei, M., & Mazzone, A. (2021). Assessment of SARS-CoV-2 Reinfection 1 Year After Primary Infection in a Population in Lombardy, Italy. JAMA internal medicine.

[5] Wadman, M. (2021). Having SARS-CoV-2 once confers much greater immunity than a vaccine—but vaccination remains vital. Science, 373(6559), 1067-8.

[6] Block, J. (2021). Vaccinating people who have had covid-19: why doesn't natural immunity count in the US?. BMJ, 374.

COMMENT 3-2. *COVID-19 is mild in younger age groups and but may cause severe infection in the elderly. Population from HIC, in that sense, are more susceptible to a severe outcome. Given that the model is not age-structured, it is essential to have a different mortality rate in HIC and LIC. I might have missed it, but I did not see that the authors accounted for the difference in death rates in HIC and LIC.*

RESPONSE 3-2. Thanks for the valuable comments. We agree with the reviewer that it is essential to have a different mortality rate in HICs and LMICs. In the revised manuscript, we set a country-specific severity (the case fatality rate) matrix in the revised manuscript to account for the heterogeneity in the healthcare burden of COVID-19 and the age structure in different countries. We calculated the country-specific case fatality rates using the Bayesian average. Denote the actual infection fatality rate for country i as IFR_i, then,

$$\mathcal{F}_{i,1} = IFR_i = \sum_a \mathcal{P}_i(deceased|infected, a)\frac{\mathcal{P}_i(infected|a)\mathcal{P}_i(a)}{\mathcal{P}_i(infected)}$$
$$\approx \sum_a CFR_{i,a}\frac{\mathcal{P}_i(infected|a)\mathcal{P}_i(a)}{\mathcal{P}_i(infected)},$$

where a denotes a specific age group, $\mathcal{P}_i(deceased|infected, a)$ represents the probability of dying from the disease for infected individuals at age group a, $CFR_{i,a}$ denotes the age-specific case fatality rate for age group a in country i, $\mathcal{P}_i(infected|a)$ represents the probability of getting infected for individuals in age group a in country i, $\mathcal{P}_i(infected)$ represents the probability of getting infected in country i, and $\mathcal{P}_i(a)$ represents the proportion of individuals in age group a, as a share of the whole population in country i. Due to limited data for age-specific case morbidity and fatality rates for a specific age group in each country, we set the same values of $CFR_{i,a}$ and $\mathcal{P}_i(infected|a)/\mathcal{P}_i(infected)$ among HICs and LMICs, i.e.,

$$CFR_{i,a} = \begin{cases} CFR_{H,a} & i \in H, \\ CFR_{L,a} & i \in L, \end{cases}$$

and

$$\frac{\mathcal{P}_i(infected|a)}{\mathcal{P}_i(infected)} = \begin{cases} \frac{\mathcal{P}_H(infected|a)}{\mathcal{P}_H(infected)} & i \in H, \\ \frac{\mathcal{P}_L(infected|a)}{\mathcal{P}_L(infected)} & i \in L. \end{cases}$$

Here, the values of $CFR_{H,a}$, $CFR_{L,a}$, $\frac{\mathcal{P}_H(infected|a)}{\mathcal{P}_H(infected)}$, and $\frac{\mathcal{P}_L(infected|a)}{\mathcal{P}_L(infected)}$ are computed based on data from India and the United States [1]. Specific values are shown in Tables S3 and S4. $\mathcal{P}_i(a)$ is adopted from the latest version of World Population Prospects [2]. Overall, the average case fatality rates among HICs and LMICs in the revised manuscript are 0.03 and 0.01, respectively. We added the following description in the main text to illustrate the variability in the case fatality rate (severity) across countries:

Method Section, **Multi-strain model** Part: "For country i, such dynamics are captured by the transmissibility matrix $\mathcal{T}$, the severity matrix $\mathcal{F}_i$, and the mutation matrix $\mathcal{U}$, all with dimensions $M \times M$…We set a country-specific severity matrix to account for the heterogeneity in the healthcare burden of COVID-19 and the age structure in different countries [3-5]."

Method Section, **Deterministic, discrete-time SVEIRD based metapopulation model** Part: "For individuals without vaccinal immunity, the transition rates from infectious (caused by strain m) to recovered and deceased are $(1-\mathcal{F}_{i,m})\alpha$ and $\mathcal{F}_{i,m}\alpha$, respectively; for individuals with vaccinal immunity, the transition rates from infectious (caused by strain m) to recovered and deceased are $[1-(1-\epsilon_m)\mathcal{F}_{i,m})]\alpha$ and $(1-\epsilon_m)\mathcal{F}_{i,m}\alpha$, respectively.

$$\partial_t R_i(t) = \sum_m (1-\mathcal{F}_{i,m})\alpha I_{i,m}^S(t) + \sum_m [1-(1-\epsilon_m)\mathcal{F}_{i,m}]\alpha I_{i,m}^V(t) + \sum_j G_{ij}(t)[\frac{R_j(t)}{A_j(t)} - \frac{R_i(t)}{A_i(t)}],$$

$$\partial_t D_i(t) = \sum_m \mathcal{F}_{i,m}\alpha I_{i,m}^S(t) + \sum_m (1-\epsilon_m)\mathcal{F}_{i,m}\alpha I_{i,m}^V(t).$$

"

A detailed description of the estimation of country-specific severity matrices could be found in Section 3 **Estimation of country-specific severity matrices** in the Supplementary Information.

References:

[1] Laxminarayan, R., Wahl, B., Dudala, S. R., Gopal, K., Neelima, S., Reddy, K. J., ... & Lewnard, J. A. (2020). Epidemiology and transmission dynamics of COVID-19 in two Indian states. Science, 370(6517), 691-697.

[2] The United Nations. United Nations World Population Prospects. (2020). https://population.un.org/wpp/

[3] Miller, I. F., Becker, A. D., Grenfell, B. T., & Metcalf, C. J. E. (2020). Disease and healthcare burden of COVID-19 in the United States. Nature Medicine, 26(8), 1212-1217.

[4] O'Driscoll, M., Dos Santos, G. R., Wang, L., Cummings, D. A., Azman, A. S., Paireau, J., ... & Salje, H. (2021). Age-specific mortality and immunity patterns of SARS-CoV-2. Nature, 590(7844), 140-145.

[5] Zhang Qingpeng. (2022). Data science approaches to infectious disease surveillance. Phil. Trans. R. Soc. A. 380(2214) 20210115.

COMMENT 3-3. *It might be more beneficial to add policy papers (i.e, advisory committees, FDA regulations etc.) that explicitly call for a vaccination with booster doses (e.g., Israel, US, UK).*

RESPONSE 3-3. Thank you for the suggestion. We have added several policy papers that call for a vaccination with booster doses in the introduction part.

The added part is copied below:

Introduction Section:

"…thus they are racing to vaccinate their entire population and expand booster-shot programs [1-4] rather than donate vaccines to LMICs to suppress the emergence of new strains."

References:

[1] (from US) U.S. Food and Drug Administration. Coronavirus (COVID-19) Update: FDA Takes Additional Actions on the Use of a Booster Dose for COVID-19 Vaccines (2021). *https://www.fda.gov/news-events/press-announcements/coronavirus-covid-19-update-fda-takes-additional-actions-use-booster-dose-covid-19-vaccines*

[2] (from UK) Mahase, E. (2021). Covid-19: Booster dose will be needed in autumn to avoid winter surge, says government adviser.

[3] (from Israel) Israel Ministry of Health. More than 4 Million Received the Booster Shot in Israel (2021). *https://www.gov.il/en/Departments/news/10112021-01.*

[4] (from Canada) Canada's National Advisory Committee on Immunization. Interim guidance on boosterCOVID-19 vaccine doses in Canada (2021). *https://www.canada.ca/en/public-health/services/immunization-vaccines.html.*

COMMENT 3-4. *The authors stated: "Thus, making COVID-19 vaccines distributed equitably is not only a moral obligation for high-income countries but also in their rational self-interest." If it has been previously found, add ref. If not, it sounds like a statement or an opinion and should not appear in the intro. I think it is part of their finding, so it should not be here. In the introduction, it might be useful to say that it has been previously shown for flu https://bmcpublichealth.biomedcentral.com/articles/10.1186/s12889-021-11601-2 , which provides a motivation for their study.*

RESPONSE 3-4. Thank you for your suggestion. Indeed, there were opinion/commentary papers but no quantitative study about this statement. This statement is part of our main conclusion. Following the reviewer's suggestion, we moved this statement to the Conclusion Section and added the following findings from previous flu research:

Introduction Section:

"It has been shown in the context of influenza that cross-border vaccination subsidies could provide substantial indirect protection to countries donating vaccines [1,2]. Offering influenza vaccines to neighboring countries can substantially reduce infections and deaths in both donating and receiving countries."

[1] Yamin, D., Kahana, D., Shahmoon, E., Fitzpatrick, M. C., & Galvani, A. P. (2021). Influenza vaccination should have no border: cost-effectiveness of cross-border subsidy. BMC public health, 21(1), 1-11.

[2] Fidler, D. P., & Gostin, L. O. (2011). The WHO pandemic influenza preparedness framework: a milestone in global governance for health. JAMA, 306(2), 200-201.

COMMENT 3-5. *The authors stated: "With these solutions, global vaccine distribution could no longer be a 'zero-sum game' but a 'cooperative game'" This has been previously considered in the context of game-theoretic model. You might want to consider this study https://pubsonline.informs.org/doi/abs/10.1287/mnsc.1120.1661*

RESPONSE 3-5. Thank you very much for the very important suggestion. We have cited and discussed this paper and other related papers in the revised manuscript.

Introduction Section:

"Such game-theoretic approach has been applied to the control of other pathogens [1-6]. Numerical and analytical results based on hypothetical networks show that the optimal drug/vaccine coordination can reduce the epidemic size and overall financial burden of infection for all countries. However, data-driven research on global vaccine coordination in real-world human mobility networks is rare, particularly in the context of the COVID-19 pandemic with viral mutations."

References:

[1] Mamani, H., Chick, S. E., & Simchi-Levi, D. (2013). A game-theoretic model of international influenza vaccination coordination. Management Science, 59(7), 1650-1670.

[2] Sun, P., Yang, L., & De Véricourt, F. (2009). Selfish drug allocation for containing an international influenza pandemic at the onset. Operations Research, 57(6), 1320-1332.

[3] Aswani, A., Shen, Z. J. M., & Siddiq, A. (2019). Data-driven incentive design in the medicare shared savings program. Operations Research, 67(4), 1002-1026.

[4] Klepac, P., Laxminarayan, R., & Grenfell, B. T. (2011). Synthesizing epidemiological and economic optima for control of immunizing infections. Proceedings of the National Academy of Sciences, 108(34), 14366-14370.

[5] Matrajt, L., Halloran, M. E., & Longini Jr, I. M. (2013). Optimal vaccine allocation for the early mitigation of pandemic influenza. PLoS Computational Biology, 9(3), e1002964.

[6] Barrett, S. (2013). Economic considerations for the eradication endgame. Philosophical Transactions of the Royal Society B: Biological Sciences, 368(1623), 20120149.

COMMENT 3-6. *The model is very clear, well written, and transparent. Please insert a clear table with the parameter values used in the model – at least the main parameters.*

RESPONSE 3-6. Thank you for your suggestion. Following the suggestion, we inserted Table S5 in the Supplementary Information to summarize the symbol, definitions, and values of all parameters we used in

the model. The table is too long to be presented in the response letter. Please see Table S5 in the Supplementary Information for details.

COMMENT 3-7. *The authors considered strategies as follows: "Population size. Priority to countries with larger population sizes. Prevalence. Priority to countries with a higher number of active cases (currently infectious cases) per capita. Incidence rate. Priority to countries with a higher incidence rate, which is defined as the number of new cases during two weeks as a share of the total population." Typically, strategies are considered in the scientific literature to work of such kind – 'morbidity based' and 'mortality based' are considered. I strongly suggest adding a mortality based strategy (i.e., prioritizing in regions of higher mortality)*

RESPONSE 3-7. Thank you very much for the valuable comments. Following the suggestion, in the revised manuscript, we added a mortality rate-based global vaccine allocation strategy in the main text and moved the incidence-based one in the Supplementary Information.

The added parts are copied below:

Results Section, **Global vaccine inequity only provides limited and short-term benefits to HICs** Part:

"Four prioritization criteria are considered: the population size, prevalence, the mortality rate, and incidence (please refer to Methods for details)."

Methods Section, **Global vaccine allocation mode** Part:

"

- Mortality rate. Priority to countries with a higher number of new deaths during two weeks as a share of the total population.

"

Section 6 **Details of global vaccine allocation strategies** in the Supplementary Information:

"

- **Equitable mortality rate-based allocation.** In the first step, available daily vaccines will be allocated to all countries according to the mortality rate, which is defined as the number of new deaths during two weeks as a share of the total population, i.e.,
$$\Omega_i^1(t) = \min\left\{dem_i(t), [\varphi(t+1) - \varphi(t)]\frac{D_i(t)/N_i - D_i(t-14)/N_i}{\sum_j D_j(t)/N_j - D_j(t-14)/N_j}\right\},$$
where $D_i(t)$ denotes the cumulative number of deaths for country i at time t.
- **Inequitable mortality rate-based allocation.** In the first step, available daily vaccines will be allocated to all countries according to the mortality rate, which is defined as the number of new deaths during two weeks as a share of the total population,
$$X(t) = \max\left\{\chi, \frac{\sum_{j\in H} D_j(t)/N_j - D_j(t-14)/N_j}{\sum_j D_j(t)/N_j - D_j(t-14)/N_j}\right\}.$$
Thus,

$$\Omega_i^1(t) = \begin{cases} \min\left\{dem_i(t), \frac{[D_i(t)/N_i - D_i(t-14)/N_i]X(t)[\varphi(t+1)-\varphi(t)]}{\sum_{j\in H} D_j(t)/N_j - D_j(t-14)/N_j}\right\} & i \in H, \\ \min\left\{dem_i(t), \frac{\left[\frac{D_i(t)}{N_i} - \frac{D_i(t-14)}{N_i}\right][1-X(t)][\varphi(t+1)-\varphi(t)]}{\sum_{j\in L} D_j(t)/N_j - D_j(t-14)/N_j}\right\} & i \in L. \end{cases}$$

"

The modified Figure 2 is copied below:

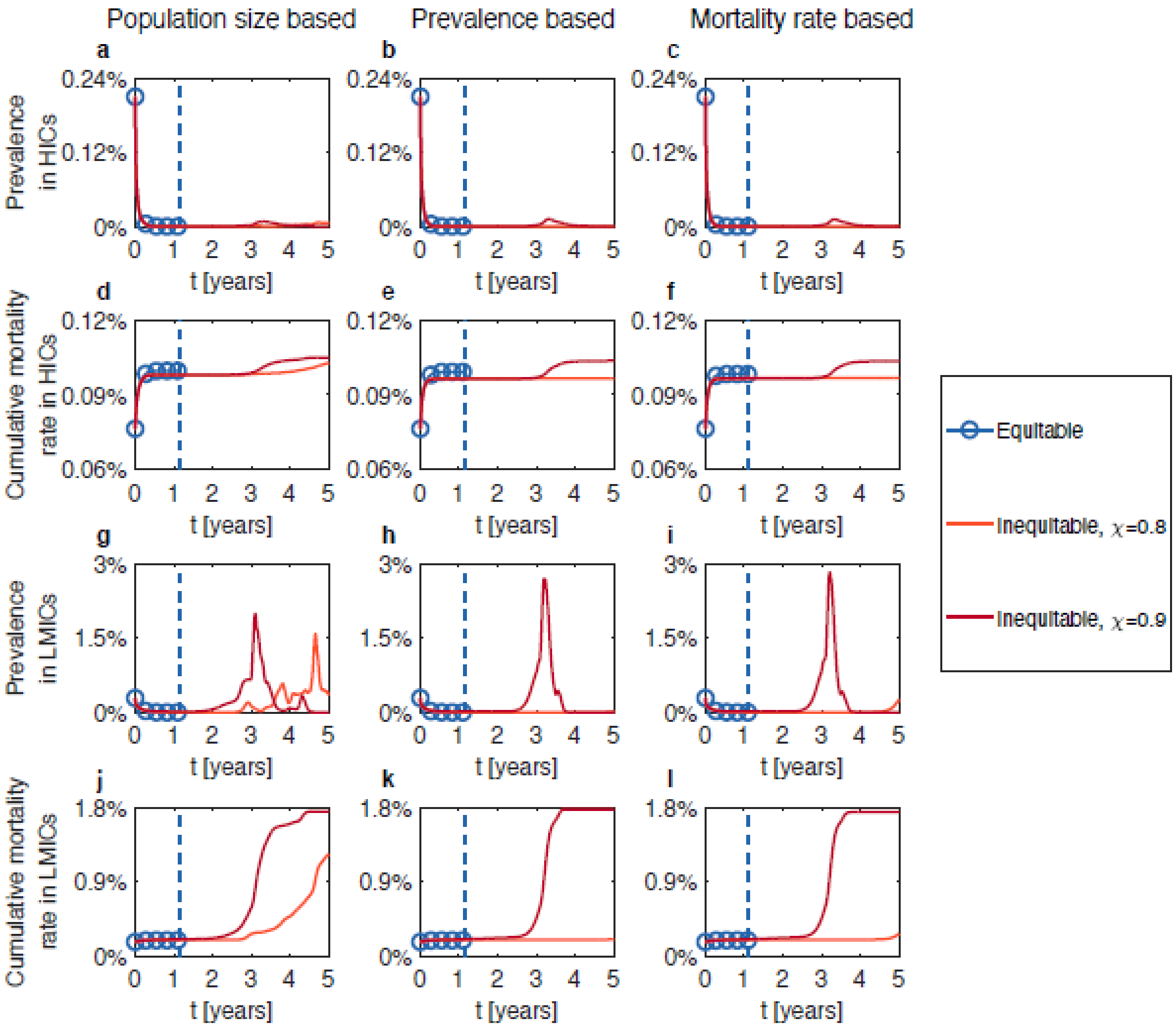

Figure 2: a-f, Time series of the prevalence (a-c) and the cumulative mortality rate (d-f) in HICs under different global vaccine allocation strategies. g-l, Time series of the prevalence (g-i) and the cumulative mortality rate (j-l) in LMICs under different global vaccine allocation strategies. Three prioritization criteria for allocation are adopted: the population size (the left panel), prevalence (the middle panel), and mortality rate (the right panel). Dash lines indicate the time when the pandemic ends (time exceeding five years is not presented). The transmissibility, severity of each strain, and the vaccine efficacy against each strain are shown in Fig. S2. Parameter values M=5, μ_1=5.6e-3, θ=0.2, and λ=500.

COMMENT 3-8. *Figures 2 and 3 present infections. Given that COVID-19 is typically mild or asymptomatic in young age groups, it is more important to present mortalities or severe outcomes.*

RESPONSE 3-8. Thank you for the valuable comments. Since we did not differentiate the severe, mild, and asymptomatic cases in our model, we presented more results about cumulative mortalities for HICs and LMICs in the revised manuscript. Specifically, we presented both the prevalence and the cumulative mortality rate in HICs and LMICs under different global vaccine allocation strategies in Figure 2 (please refer to **RESPONSE 3-7**); we used the cumulative morality rate (instead of the fraction of cumulative cases in the initial submission) as the indicator to compare the effectiveness of different vaccine donation strategies in Figure 5.

The modified figures are copied below:

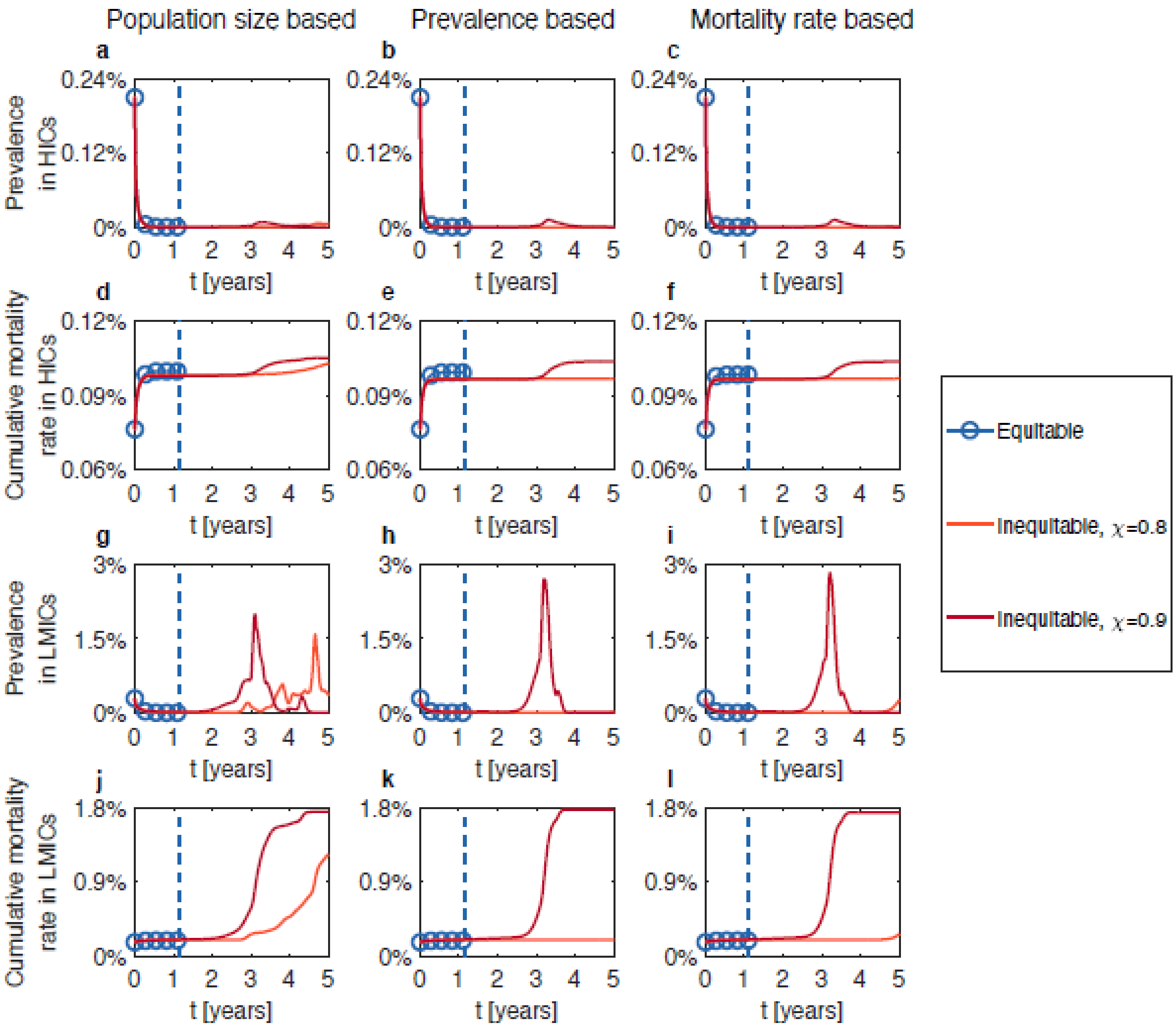

Figure 2: a-f, Time series of the prevalence (a-c) and the cumulative mortality rate (d-f) in HICs under different global vaccine allocation strategies. g-l, Time series of the prevalence (g-i) and the cumulative mortality rate (j-l) in LMICs under different global vaccine allocation strategies. Three prioritization

criteria for allocation are adopted: the population size (the left panel), prevalence (the middle panel), and mortality rate (the right panel). Dash lines indicate the time when the pandemic ends (time exceeding five years is not presented). The transmissibility, severity of each strain, and the vaccine efficacy against each strain are shown in Fig. S2. Parameter values M=5, μ_1=5.6e-3, θ=0.2, and λ=500.

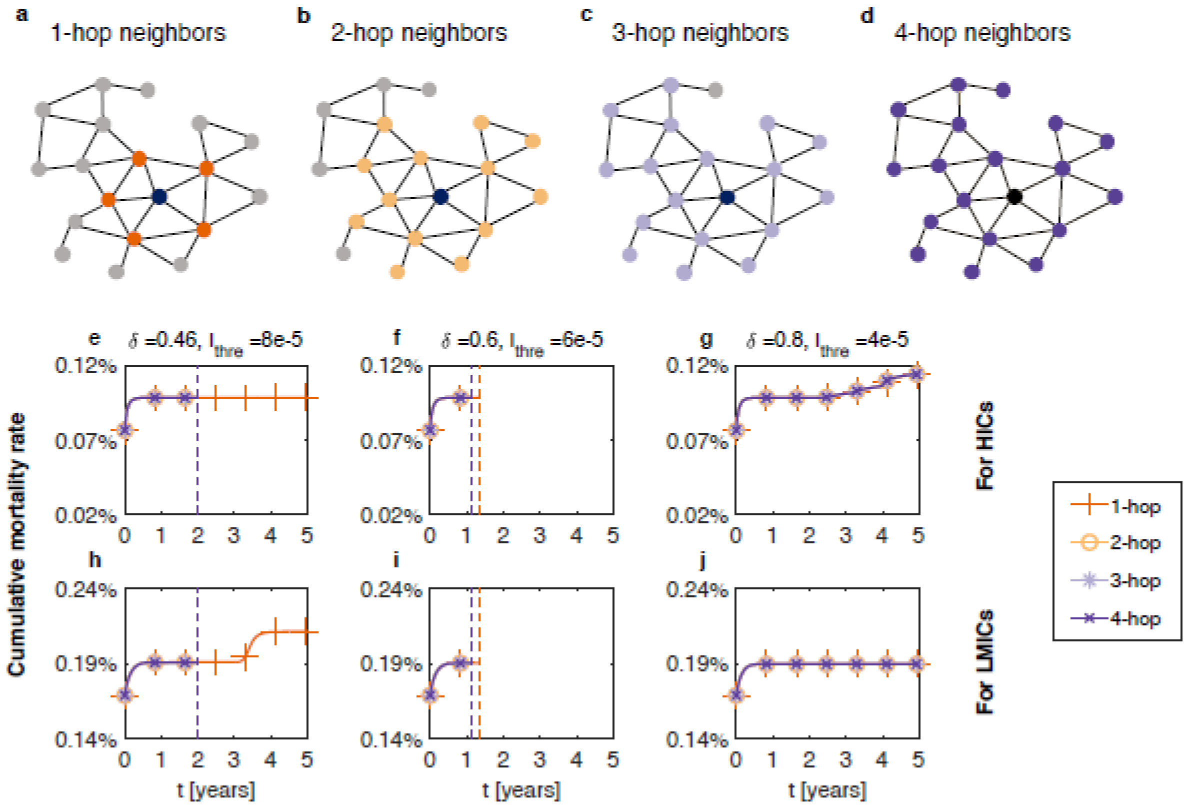

Figure 5: a-d, A country (the black node) and its 1-hop (a), 2-hop (b), 3-hop (c), and 4-hop (d) neighbors on the global mobility network (Fig. S27) constructed based on the air traffic data. e-j, Cumulative mortality rate in HICs (e-g) and LMICs (h-j) over time if HICs only donate vaccines to their 1-hop, 2-hop, 3-hop, and 4-hop LMIC neighbors under scenarios where δ=0.46 and I_{thre}=8e-5 (e and h), δ=0.6 and I_{thre}=6e-5 (f and i), δ=0.8 and I_{thre}=4e-5 (g and j). Dash lines indicate the time when the pandemic ends. Countries with larger population sizes are prioritized for vaccination. Parameter values M=5, μ_1=5.6e-3, θ=0.2, and λ=500.

COMMENT 3-9. *Discussion*

Please add two limitations and try to explain if they should affect your main outcomes:

1) waning immunity following infection (i.e., moving from recovered to or at least susceptible)

2) age-structured model

I would like to wish the authors the best of luck in addressing the review.

RESPONSE 3-9. Thanks for your suggestion. Although we did not present the results considering the waning of natural immunity in the main text, we have added the discussion of how this may affect our results in the **Conclusion** Section in the Supplementary Information (see **RESPONSE 3-1**). We also set a country-specific severity matrix in the revised manuscript to characterize the heterogeneity in the healthcare burden of COVID-19 and the age structure worldwide (see **RESPONSE 3-2**). The added part is copied below:

Conclusion section:

"Our research has limitations. First, although we derive a country-specific severity matrix to model the heterogeneous age structures across different countries, the model is not stratified by age within each country. The difference in age structures results in heterogeneous infection fatality rates and heterogeneous susceptibility to infection [1-4]. Due to limited data of the susceptibility to infection among different age groups, and the lack of age-mixing patterns for different countries, we do not parameterize an age-stratified model for each country. The model can be easily calibrated if such data was available. Second, according to the findings that reinfections are uncommon in the general population [5-8], we investigated the life-long and different short-lived natural immunity settings. Sensitivity analysis (Fig.~S25 and S26) shows that, if natural immunity is short-lived, global vaccine inequity provides even smaller benefits to HICs. Future research should incorporate the more realistic natural immunity duration data for different strains (if available)."

References:

[1] Davies, N. G., Klepac, P., Liu, Y., Prem, K., Jit, M., & Eggo, R. M. (2020). Age-dependent effects in the transmission and control of COVID-19 epidemics. Nature medicine, 26(8), 1205-1211.

[2] Trentini, F., Guzzetta, G., Galli, M., Zardini, A., Manenti, F., Putoto, G., ... & Poletti, P. (2021). Modeling the interplay between demography, social contact patterns, and SARS-CoV-2 transmission in the South West Shewa Zone of Oromia Region, Ethiopia. BMC medicine, 19(1), 1-13.

[3] Zhang Qingpeng. (2022). Data science approaches to infectious disease surveillance. Phil. Trans. R. Soc. A. 380(2214) 20210115.

[4] Burghardt Keith, Guo Siyi, & Lerman Kristina. (2022). Unequal impact and spatial aggregation distort COVID-19 growth rates Phil. Trans. R. Soc. A. 380(2214) 20210122.

[5] Turner, J. S., Kim, W., Kalaidina, E., Goss, C. W., Rauseo, A. M., Schmitz, A. J., ... & Ellebedy, A. H. (2021). SARS-CoV-2 infection induces long-lived bone marrow plasma cells in humans. Nature, 1-5.

[6] Vitale, J., Mumoli, N., Clerici, P., De Paschale, M., Evangelista, I., Cei, M., & Mazzone, A. (2021). Assessment of SARS-CoV-2 Reinfection 1 Year After Primary Infection in a Population in Lombardy, Italy. JAMA internal medicine.

[7] Wadman, M. (2021). Having SARS-CoV-2 once confers much greater immunity than a vaccine—but vaccination remains vital. Science, 373(6559), 1067-8.

[8] Block, J. (2021). Vaccinating people who have had covid-19: why doesn't natural immunity count in the US?. BMJ, 374.

Decision Letter, first revision:

22nd December 2021

Dear Dr. Zhang,

Thank you for submitting your revised manuscript "Promoting equitable access to COVID-19 vaccines makes a life-saving difference to all countries" (NATHUMBEHAV-210816308A). It has now been seen by the original referees and their comments are below. As you can see, the reviewers find that the paper has improved in revision. We will therefore be happy in principle to publish it in Nature Human Behaviour, pending minor revisions to satisfy the referees' final requests and to comply with our editorial and formatting guidelines. Please note that in addition to the reviews included below, Reviewer #3 has submitted confidential remarks to the editors, recommending publication of your work with no further requests.

We are now performing detailed checks on your paper and will send you a checklist detailing our editorial and formatting requirements by tomorrow, so that you could work on the final revisions within the next couple of weeks, aiming to resubmit in the first weeks of January. Given the timeliness of your findings, we are hoping to be able to publish your work by the end of January. **Please do not upload the final materials and make any revisions until you receive this additional information from us.**

Please do not hesitate to contact me if you have any questions.

Sincerely,

Arunas Radzvilavicius, PhD
Editor
Nature Human Behaviour

Reviewer #2 (Remarks to the Author):

I thank the authors for their very comprehensive response and revision of the paper. I believe that the changes made have significantly strengthened their study.

Minor comments:
1) In Figure 2 (and analogous figures in the Supplementary information) it is not clear to which allocation strategy the dashed line refers, since epidemic ends does not occur in all allocation strategies shown.

Please specify in the caption that dashed lines refers to the strategy defined by the same colour (or add an additional legend on the right).
2) In Figure 4f on the total number of doses. Are the y-axis labels correct? I find the presence of “%” on the y-axis labels and “x10^7” on the top of the plot confusing.

Reviewer #4 (Remarks to the Author):

Dear prof. Arunas Radzvilavicius,

I went over the revised paper and their reply to my comments. I think the authors made a wonderful job. They fully addressed all of my comments.

I also went over their code (please note, the file you shared with me had some error, but I searched and found this link-
https://github.com/jianan0099/VACEquity_initial). It is very well documented and highly transparent.

I think their key messages are of high interest and are timely. I, therefore, think the journal will greatly benefit from a fast publication (particularly now, with the Omicron...). Thus, I highly recommend accepting the paper.

I hereby declare no conflict of interest and would like to wish the authors the best of luck.

Author Rebuttal, first revision:

Dear editors and reviewers,

Thank you so much for taking the time to review this manuscript. We really appreciate all your comments and suggestions! We hope that the revised manuscript could meet the requirements for Nature Human Behaviour.

All revised portions are marked in red in the revised manuscript. The main comments and our specific responses are detailed below.

REVIEWER 2'S COMMENTS

COMMENT 2-1. *In Figure 2 (and analogous figures in the Supplementary information) it is not clear to which allocation strategy the dashed line refers, since epidemic ends does not occur in all allocation strategies shown. Please specify in the caption that dashed lines refers to the strategy defined by the same colour (or add an additional legend on the right).*

RESPONSE 2-1. Thank you for the comment. Following the suggestion, we specify in the caption of Figure 2 (and analogous figures in the Supplementary Information) that "dashed lines referring to the priority criterion are represented by the same colour". The revised Figure 2 is copied below:

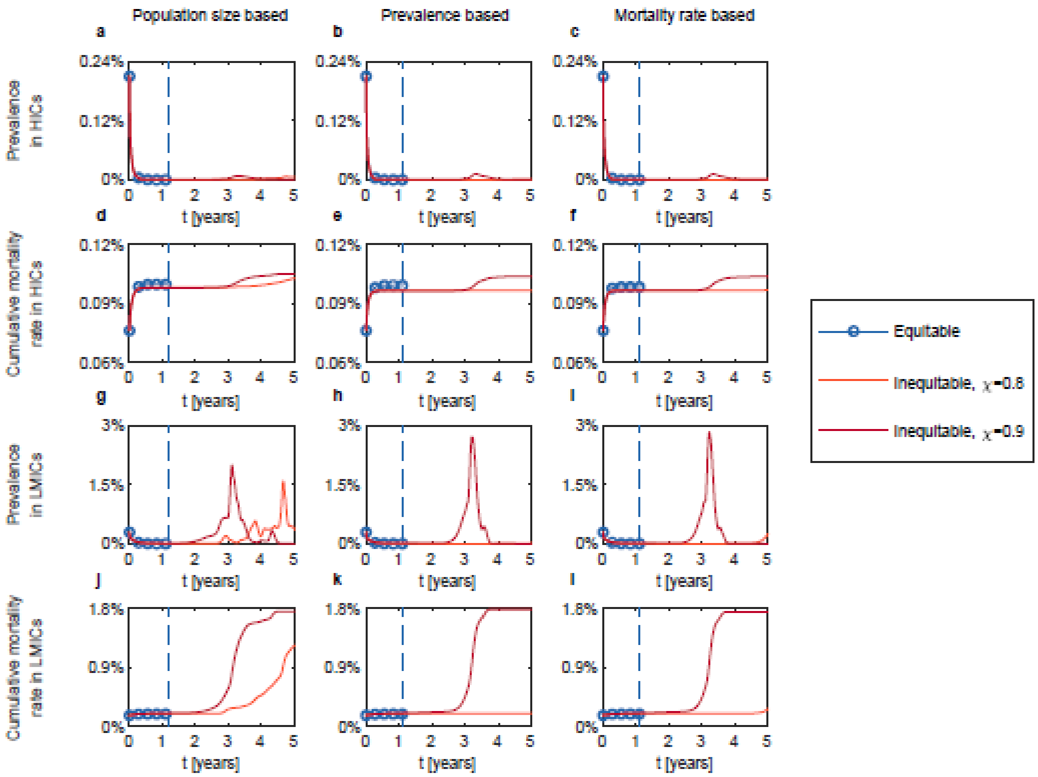

Figure 2: Impact of equitable and inequitable vaccine allocation strategies on epidemic dynamics. a-f, Time series of the prevalence (a-c) and the cumulative mortality rate (d-f) in HICs under different global

vaccine allocation strategies. g-l, Time series of the prevalence (g-i) and the cumulative mortality rate (j-l) in LMICs under different global vaccine allocation strategies. Three prioritization criteria for allocation are adopted: the population size (the left panel), prevalence (the middle panel), and mortality rate (the right panel). Dash lines indicate the time when the pandemic ends (time exceeding five years is not presented; dashed lines referring to the priority criterion are represented by the same colour). The transmissibility, severity of each strain, and the vaccine efficacy against each strain are shown in Supplementary Fig. 2. Parameter values M=5, μ_1=5.6×10^{-3}, θ=0.2, and λ=5×10^2.

COMMENT 2-2. *In Figure 4f on the total number of doses. Are the y-axis labels correct? I find the presence of "%" on the y-axis labels and "x10^7" on the top of the plot confusing.*

RESPONSE 2-2. Thank you for pointing out the typo. We have removed the "%" symbol in the y-axis labels of Figure 4f in the main text and analogous figures in the Supplementary Information. The revised Figure 4 is copied below:

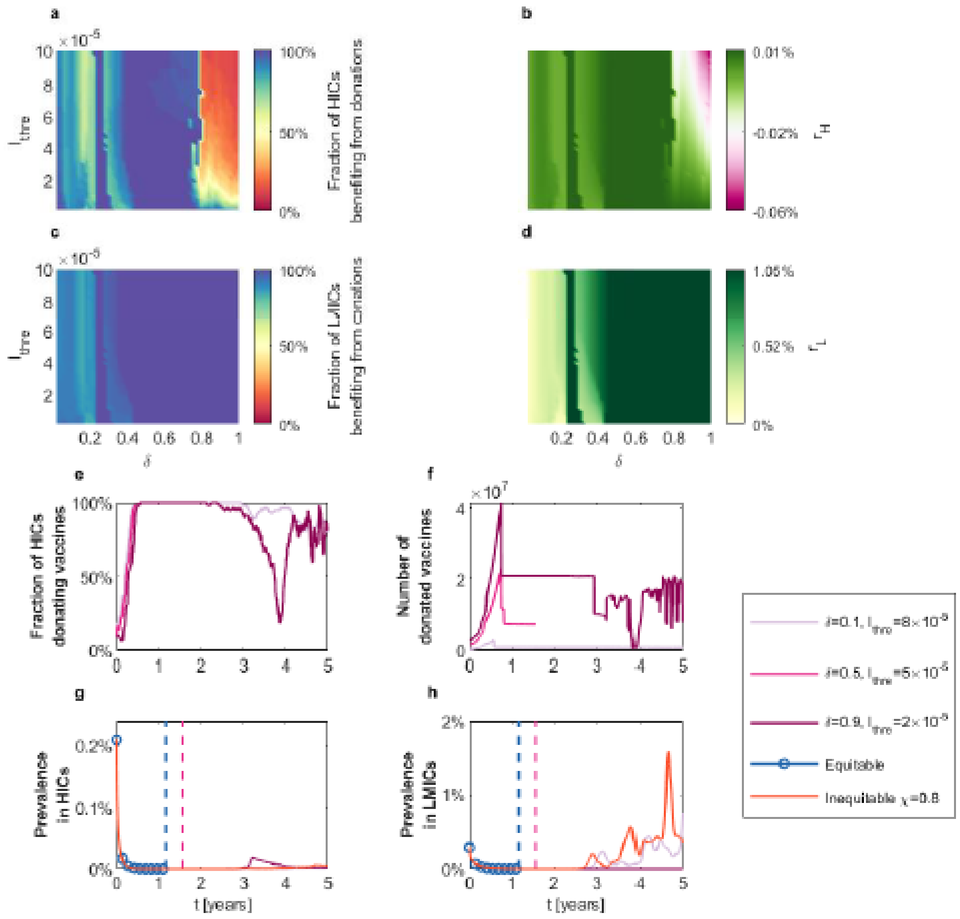

Figure 4: Impact of different allow-donation vaccine allocation strategies on epidemic dynamics. a and c, Fraction of HICs and LMICs benefiting from donations. b and d, Average lives saved by vaccine

donations as the share of the national population in HICs (r_H) and LMICs (r_L). Please refer to Methods section for details of r_H and r_L. e, Fraction of HICs donating vaccines. f, Total number of donated vaccines. g and h, Prevalence in HICs and LMICs under different vaccine allocation strategies. Dash lines indicate the time when the pandemic ends. Countries with larger population sizes are prioritized for vaccination. Parameter values M=5, μ_1=5.6×10^{-3}, θ=0.2, and λ=5×10^2.

Final Decision Letter:

Dear Professor Zhang,

We are pleased to inform you that your Article "Equitable access to COVID-19 vaccines makes a lifesaving difference to all countries", has now been accepted for publication in Nature Human Behaviour. Given how timely your article is, we will aim to fast track further processing/production.

Please note that *Nature Human Behaviour* is a Transformative Journal (TJ). Authors whose manuscript was submitted on or after January 1st, 2021, may publish their research with us through the traditional subscription access route or make their paper immediately open access through payment of an article-processing charge (APC). Authors will not be required to make a final decision about access to their article until it has been accepted. IMPORTANT NOTE: Articles submitted before January 1st, 2021, are not eligible for Open Access publication. Find out more about Transformative Journals

Authors may need to take specific actions to achieve compliance with funder and institutional open access mandates. For submissions from January 2021, if your research is supported by a funder that requires immediate open access (e.g. according to Plan S principles) then you should select the gold OA route, and we will direct you to the compliant route where possible. For authors selecting the subscription publication route our standard licensing terms will need to be accepted, including our self-archiving policies. Those standard licensing terms will supersede any other terms that the author or any third party may assert apply to any version of the manuscript.

Before your manuscript is typeset, we will edit the text to ensure it is intelligible to our wide readership and conforms to house style. We look particularly carefully at the titles of all papers to ensure that they are relatively brief and understandable.

Once your manuscript is typeset and you have completed the appropriate grant of rights, you will receive a link to your electronic proof via email with a request to make any corrections within 48 hours. If, when you receive your proof, you cannot meet this deadline, please inform us at rjsproduction@springernature.com immediately. Once your paper has been scheduled for online publication, the Nature press office will be in touch to confirm the details.

Acceptance of your manuscript is conditional on all authors' agreement with our publication policies (see http://www.nature.com/nathumbehav/info/gta). In particular your manuscript must not be published elsewhere and there must be no announcement of the work to any media outlet until the publication date (the day on which it is uploaded onto our web site).

If you have posted a preprint on any preprint server, please ensure that the preprint details are updated with a publication reference, including the DOI and a URL to the published version of the article on the journal website.

An online order form for reprints of your paper is available at https://www.nature.com/reprints/author-reprints.html. All co-authors, authors' institutions and authors' funding agencies can order reprints using the form appropriate to their geographical region.

You can now use a single sign-on for all your accounts, view the status of all your manuscript submissions and reviews, access usage statistics for your published articles and download a record of your refereeing activity for the Nature journals.

To assist our authors in disseminating their research to the broader community, our SharedIt initiative provides you with a unique shareable link that will allow anyone (with or without a subscription) to read the published article. Recipients of the link with a subscription will also be able to download and print the PDF.

As soon as your article is published, you will receive an automated email with your shareable link.

In approximately 10 business days you will receive an email with a link to choose the appropriate publishing options for your paper and our Author Services team will be in touch regarding any additional information that may be required.

You will not receive your proofs until the publishing agreement has been received through our system.

If you have any questions about our publishing options, costs, Open Access requirements, or our legal forms, please contact ASJournals@springernature.com

We look forward to publishing your paper.

With best regards,

Arunas Radzvilavicius, PhD
Editor
Nature Human Behaviour
